# Development of a Fluorescent Assay and Imidazole-Containing Inhibitors by Targeting SARS-CoV-2 Nsp13 Helicase

**DOI:** 10.3390/molecules29102301

**Published:** 2024-05-14

**Authors:** Chuang Zhang, Junhui Yu, Mingzhenlong Deng, Qingqing Zhang, Fei Jin, Lei Chen, Yan Li, Bin He

**Affiliations:** 1 State Key Laboratory of Functions and Applications of Medicinal Plants, Engineering Research Center for the Development and Application of Ethnic Medicine and TCM (Ministry of Education), Guizhou Provincial Key Laboratory of Pharmaceutics, School of Pharmacy, Guizhou Medical University, Guiyang 550004, China; 2 School of Basic Medical Science, Guizhou Medical University, Guiyang 550004, China

**Keywords:** coronary virus, Nsp13, activity assay, inhibitor, imidazole derivatives

## Abstract

Nsp13, a non-structural protein belonging to the coronavirus family 1B (SF1B) helicase, exhibits 5′–3′ polarity-dependent DNA or RNA unwinding using NTPs. Crucially, it serves as a key component of the viral replication–transcription complex (RTC), playing an indispensable role in the coronavirus life cycle and thereby making it a promising target for broad-spectrum antiviral therapies. The imidazole scaffold, known for its antiviral potential, has been proposed as a potential scaffold. In this study, a fluorescence-based assay was designed by labeling dsDNA substrates with a commercial fluorophore and monitoring signal changes upon Nsp13 helicase activity. Optimization and high-throughput screening validated the feasibility of this approach. In accordance with the structural characteristics of ADP, we employed a structural-based design strategy to synthesize three classes of imidazole-based compounds through substitution reaction. Through in vitro activity research, pharmacokinetic parameter analysis, and molecular docking simulation, we identified compounds A16 (IC_50_ = 1.25 μM) and B3 (IC_50_ = 0.98 μM) as potential lead antiviral compounds for further targeted drug research.

## 1. Introduction

Coronaviruses, enveloped RNA viruses, are notorious for their transmissible nature between humans and animals, capable of causing a wide range of illnesses from mild respiratory infections to gastrointestinal, hepatic, and neurological complications. By the start of the 21st century, two major outbreaks were attributed to coronaviruses: the Severe Acute Respiratory Syndrome (SARS) in 2002 and the Middle East Respiratory Syndrome (MERS) in 2012 [1,2]. In late 2019, a new coronavirus, designated as 2019-nCoV, erupted into a massive global pandemic of viral pneumonia. Its highly contagious nature facilitated rapid worldwide transmission, outpacing both SARS and MERS in terms of infection numbers and geographic reach and posing an unprecedented threat to global public health [3,4]. The swift spread of SARS-CoV-2 can be attributed to its high reproductive rate, transmission within close-knit communities, silent transmission, and airborne spread through respiratory droplets and aerosols. Affected individuals may experience severe low oxygen levels, viral pneumonia, acute respiratory distress syndrome, and gastrointestinal and neurological symptoms. Consequently, on 30 January 2020, the World Health Organization elevated the COVID-19 situation to a Public Health Emergency of International Concern [5]. On 11 February 2020, the International Committee on Taxonomy of Viruses officially named the virus SARS-CoV-2, while the WHO designated the illness as COVID-19, subsequently anticipating a pandemic on 11 March 2020.

SARS-CoV-2, a member of the Betacoronavirus genus, is an enveloped, positive-sense, single-stranded RNA virus that shares similarities with other coronaviruses. It circulates widely between humans and animals and is capable of causing a spectrum of illnesses ranging from mild to severe respiratory infections, as well as gastrointestinal, hepatic, and neurological diseases. A crucial aspect of the virus’s replication cycle involves five proteases, among which helicase (Helicase) presents a promising antiviral target. Nsp13, a Superfamily 1B helicase, exhibits DNA and RNA unwinding activity in a 5′-to-3′ direction [6,7]. Comprised of five domains, including a zinc-binding domain (ZBD), stalk, 1B domain, and two RecA-like ATPase domains, the NTPase active site is pinpointed at the base between the A1 and A2 domains [8]. This site predominantly consists of six amino acids: Lys288, Ser289, Asp374, Glu375, Gln404, and Arg567 [9]. Research indicates that suppressing helicase activity can be achieved by inhibiting ATPase activity, as helicase function relies on ATP hydrolysis for energy. Therefore, a series of compounds can be designed to compete with NTPs at the NTPase active site, thereby indirectly hindering Nsp13’s helicase functionality.

**Figure 1:**
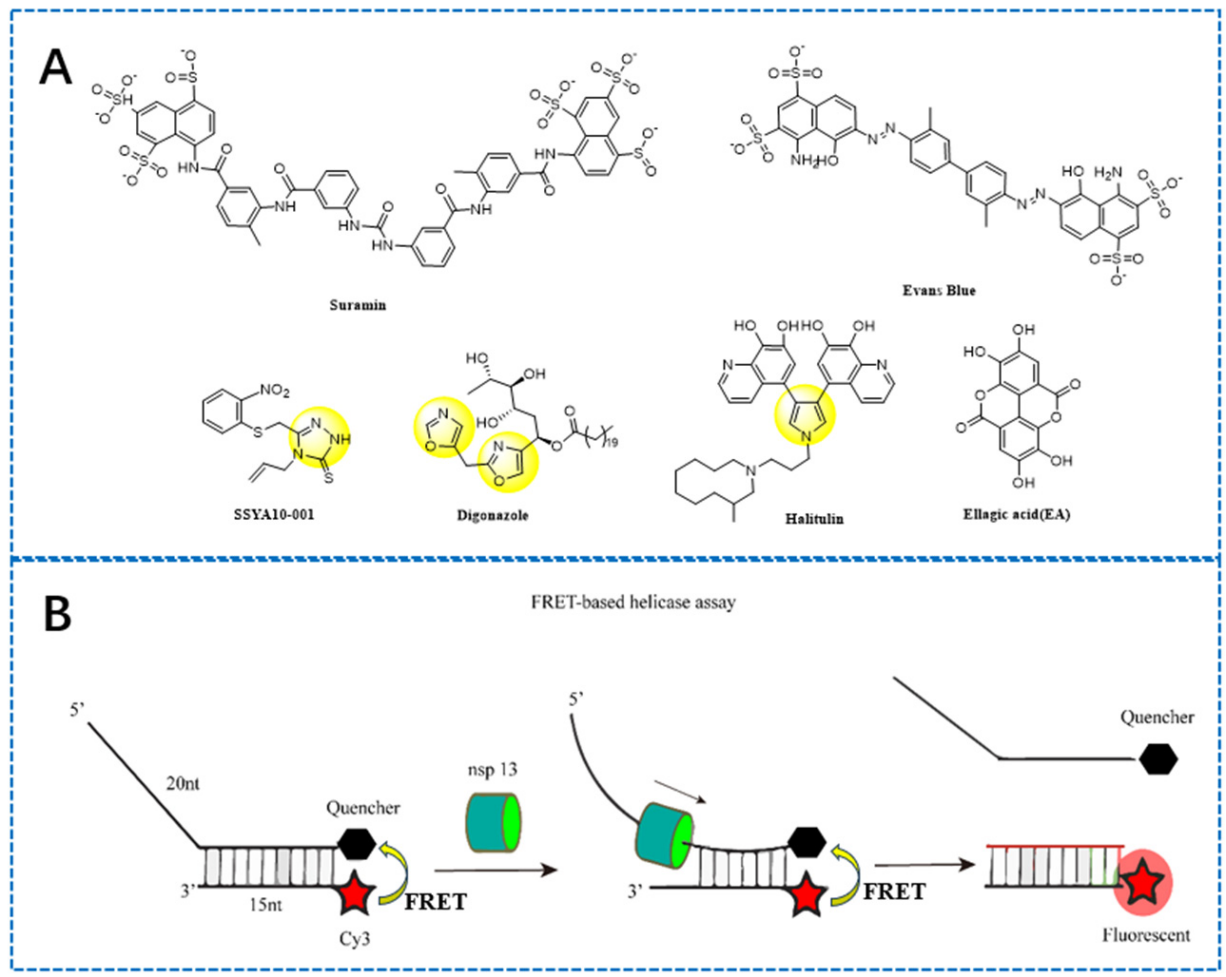
(**A**) Several known SARS-CoV-2 Nsp13 helicase inhibitors (the yellow color highlights the five-membered heterocycle moiety) (**B**) A fluorescence resonance energy transfer (FRET) for SARS-CoV-2 Nsp13 helicase (the red color highlights the fluorescence on).

Studies have identified several inhibitors of Nsp13 helicase, such as suramin, Evans blue [10], SSYA10-001 [11], digonazole, halitulin [8], and ellagic acid (EA) [12] (Figure 1A). However, many of these inhibitors exhibit complex structures and suboptimal inhibitory profiles. Intriguingly, some possess a scaffold similar to that of imidazole, suggesting that a structural transition involving the replacement of the five-membered ring with an imidazole moiety could potentially lead to the synthesis of novel Nsp13 helicase inhibitors, warranting extensive investigation.

The imidazole skeleton structure is a very practical and versatile structural/functional nuclear that can be considered a rich source of chemical diversity [13]. One of the key proteins in SARS-CoV-2, Nsp13 strandase, has been only reported with its in vitro activity detection method, which utilizes the fluorescence energy transfer resonance (FRET) principle [10] (Figure 1B). However, the synthesis of this method requires the preparation of complementary double strands containing two color groups, which is difficult to do and costly. Thus, there is still a great need to develop a simple and rapid method for detecting the in vitro activity of Nsp13 strandase. Most Nsp13 strandases have complex structures, and there are also many simple and easy-to-obtain Nsp13 strandases awaiting discovery. Therefore, in this study, the authors developed an efficient and rapid in vitro fluorescent detection method for SARS-CoV-2 Nsp13 strandase using commercially available dsDNA fluorescence dye (AccuBlue^®^ High Sensitivity dsDNA Quantitation). Compared with current detection methods, the method devised in this study does not require the preparation of complementary double strands containing two color groups; ordinary complementary doubles were used, reducing costs. In addition, based on the structural characteristics of ADP, a strategy was developed to design and synthesize three types of imidazole skeleton-containing compounds through in vitro activity research with the aim of obtaining inhibitory compounds against Nsp13 strandase activity.

## 2. Results

### 2.1. The Design of a Fluorescent Assay of SARS-CoV-2 Nsp13 Helicase

**Figure 2:**
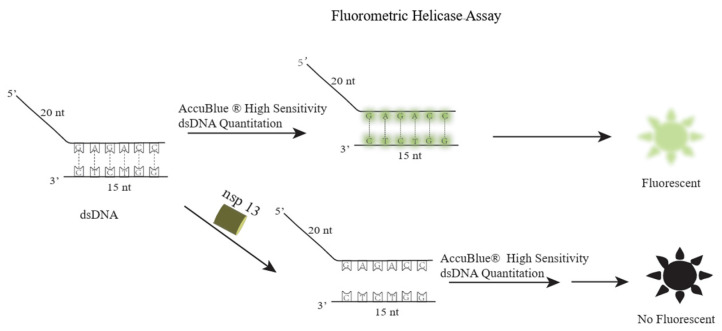
The principle of a fluorescent assay of SARS-CoV-2 Nsp13 helicase.

Short-strand DNA and overhang-strand DNA were annealed in specific proportions to yield the dsDNA substrate, which was successfully synthesized as confirmed by agarose gel electrophoresis (Figure S1). Commercial fluorescent dyes were employed to stain the dsDNA substrate, resulting in detectable fluorescent signals. Leveraging the mechanism of Nsp13 helicase, an innovative fluorescence-based assay was devised, schematically illustrated in Figure 2. The dye effectively labels the dsDNA substrate, producing fluorescence, which then fades away when the substrate is unwound into single strands by the action of Nsp13 helicase. Short-strand DNA and overhang-strand DNA sequences used in this context are described as follows:

The short-strand DNA is 5′-GGTAGTAATCCGCTC-3′
The overhang-strand DNA is 5′-TTTTTTTTTTTTTTTTTTTTGAGCGGATTACTACC-3′

### 2.2. Condition Optimization

**Figure 3:**
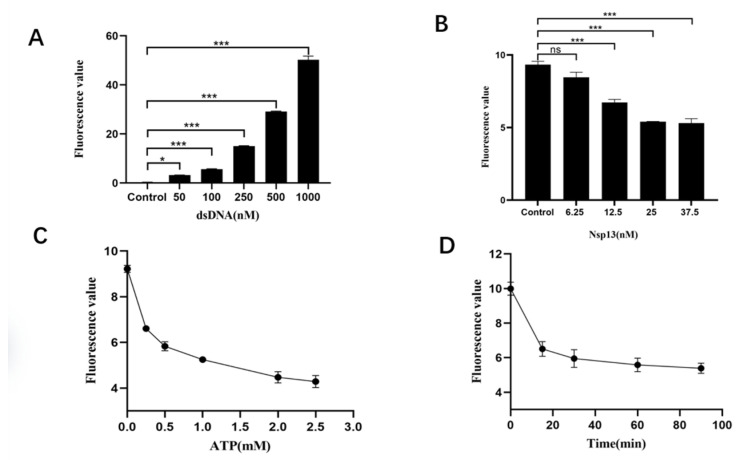
Fluorescence detection method condition optimization includes (**A**) dsDNA substrate concentration; (**B**) Nsp13 helicase concentration; (**C**) ATP concentration; and (**D**) incubation time. dsDNA concentrations were assessed using AccuBlue^®^ High Sensitivity dsDNA Quantitation dye. Data are presented as mean ± SEM. The single-piece Student *t*-test was used to analyze the statistical significance. * *p* < 0.05; *** *p* < 0.001; ns, no significance. These data represent at least three separate experiments.

To enhance the sensitivity of the Nsp13 activity assays for 96-well plate high-throughput analysis, we systematically optimized parameters such as dsDNA substrate, Nsp13 concentration, ATP levels, and incubation duration. Initially, we investigated dsDNA substrate concentrations, diluting them to 50, 100, 250, 500, and 1000 nM. Fluorescent-dye staining followed by plate-reader measurements revealed a dose-dependent increase in fluorescence intensity (Figure 3A). Considering the costs and quantities of the dye, we proceeded with a 250 nM dsDNA substrate for further optimization. Next, we examined the protein concentration by varying the Nsp13 helicase in a gradient of 6.25, 12.5, 25, and 37.5 nM. Notably, the fluorescence signal remained virtually unchanged at 25 nM Nsp13 helicase, indicating this as the optimal concentration (Figure 3B). Using the same approach, we determined the optimal ATP concentration to be 2.0 mM (Figure 3C) and the optimal incubation time to be 60 min (Figure 3D). Ultimately, the optimal reaction conditions for high-throughput screening and subsequent experiments were established at a 250 nM dsDNA substrate, 25 nM Nsp13 helicase, 2.0 mM ATP, and 60 min incubation period. These settings ensure efficient and sensitive assay performance in a high-throughput format.

### 2.3. Development of a High-Throughput Screening Method Using Fluorescent Assay of SARS-CoV-2 Nsp13 Helicase

**Figure 4:**
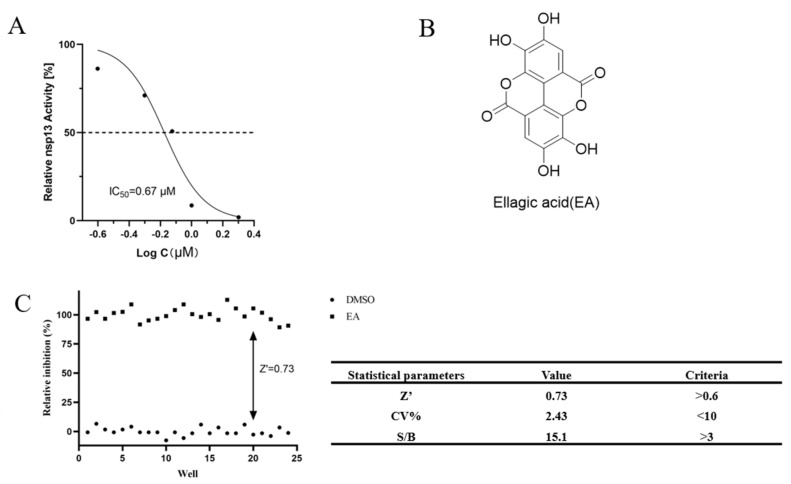
Reliability and repeatability of the high-throughput screening system for SARS-CoV-2 Nsp13 helicase activity assay: (**A**) IC_50_ value of Nsp13 helicase inhibitor EA and (**B**) structural formula of Nsp13 helicase inhibitor EA. (**C**) Determination of high-throughput parameters Z′ value and CV%. Statistical significance analysis and nonlinear regression analysis of IC_50_ were performed using GraphPad Prism^®^ Software V.9.0 for Windows (GraphPad Software Inc., San Diego, CA, USA).

Employing the optimized assay conditions, the IC_50_ value of ellagic acid (EA) for inhibiting Nsp13 helicase activity was determined to be 0.67 µM (Figure 4A,B), confirming that the developed method is suitable for assessing this enzyme’s function. The assay was validated using EA as a positive control and DMSO as a negative control to evaluate its discriminatory power for Nsp13 helicase activity. The Z′ score, coefficient of variation (CV%), and signal-to-background ratio (S/B) met the criteria for high-throughput screening (Figure 4C). These findings collectively demonstrated the reliability of the SARS-CoV-2 Nsp13 helicase activity fluorescence assay system, making it an effective tool for screening Nsp13 helicase inhibitors.

### 2.4. Design

**Figure 5:**
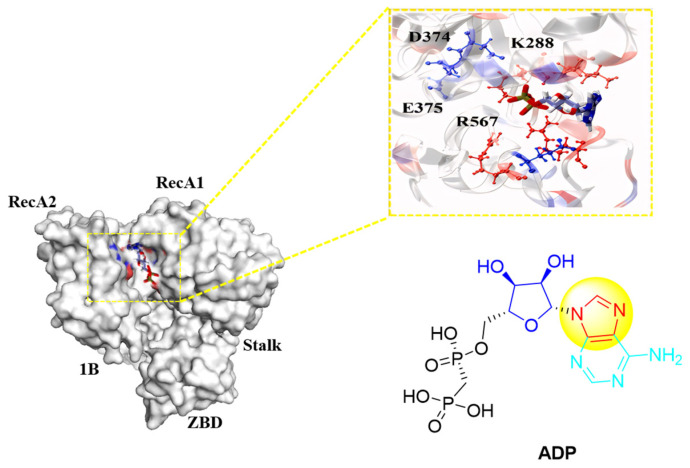
Simulation of docking between SARS-CoV-2 Nsp13 and ADP molecules (the yellow color highlights the imidazole moiety).

Research indicates that the activity of Nsp13 helicase can be indirectly reduced by inhibiting its ATPase (NTPase) function, since the helicase’s operation relies on ATP hydrolysis for an energy supply. The NTPase active site is primarily composed of six amino acids: Lys288, Ser289, Asp374, Glu375, Gln404, and Arg567 [9]. Can a series of compounds be designed to competitively bind to the NTPase active site with ATP, thereby indirectly suppressing Nsp13 helicase activity? Given that ATP converts to ADP upon hydrolysis, a molecular docking simulation with ADP (PDB ID: 6ZSL) [14] revealed that ADP interacts with residues such as Lys288, Asp374, Glu375, and Arg567, suggesting potential competition for the NTPase active site (Figure 5).

**Figure 6:**
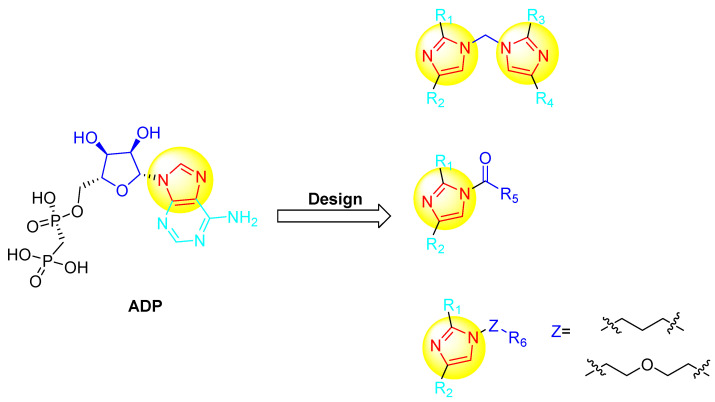
The design of imidazole derivatives as a novel Nsp13 helicase inhibitor (the yellow color highlights the imidazole moiety).

The structure of ADP consists of two phosphate groups, a ribose moiety, and an adenine base, with the adenine itself composed of a pyrimidine and an imidazole ring, which is pivotal for its interaction with the NTPase active site. Harnessing the structural characteristics of ADP, three series of compounds containing an imidazole scaffold were designed using a structure-guided approach (Figure 6). The first set involved linking two imidazole groups to create a dibenzimidazole framework, aiming to increase the number of imidazole units for enhanced activity. In the second series, a single imidazole moiety was attached through a carbonyl chain to various substituents, featuring one imidazole core; the strategy was to enhance activity by incorporating either hydrophilic or hydrophobic groups. Lastly, the third type of compound featured an imidazole moiety connected via a carbon chain and a PEG linker to different substituents, with the objective of investigating the effect of distinct linker chains on compound efficacy (Table S1).

### 2.5. Chemistry

The structural elucidation of the synthesized compound was carried out through a rigorous series of spectral analyses employing techniques like infrared spectroscopy (IR), proton nuclear magnetic resonance (^1^H NMR), carbon-13 nuclear magnetic resonance (^13^C NMR), and mass spectrometry, providing a definitive fingerprint of its molecular constitution.

#### 2.5.1. Synthesis of Compounds **A1**–**17** (General Program)

**Scheme 1:**
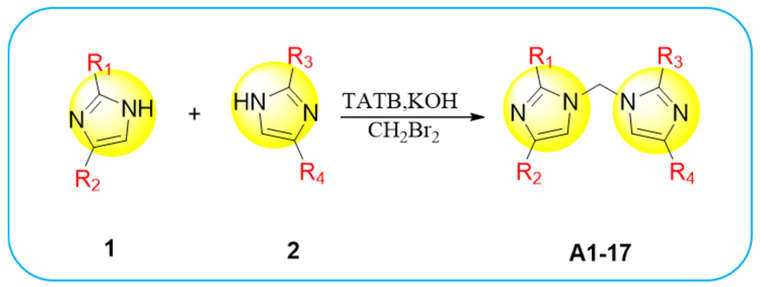
Synthesis plan containing diemole skeleton derivatives **A1**–**17** (the yellow color highlights the imidazole moiety).

Compounds **A1**–**17** are synthesized following the general procedure of Scheme 1. A mixture of compound **1** (0.71 mmol, 1 equiv) and compound **2** (0.71 mmol, 1 equiv) is initially prepared. Potassium hydroxide (100 mg, 1.8 mmol, 2.5 equiv) and TBAB (64.5mg, 0.14 mmol, 0.2 equiv) are then added, followed by dissolution in 10 mL of CH_2_Br_2_ at 60 °C. The reaction progress is monitored using thin-layer chromatography (TLC). Upon completion, the mixture is extracted with dichloromethane, washed with a saturated sodium chloride solution, and the solvent is removed under vacuum. Finally, the compounds **A1**–**17** are isolated through column chromatography.

#### 2.5.2. Synthesis of Compounds **B1**–**16** (General Program)

**Scheme 2:**
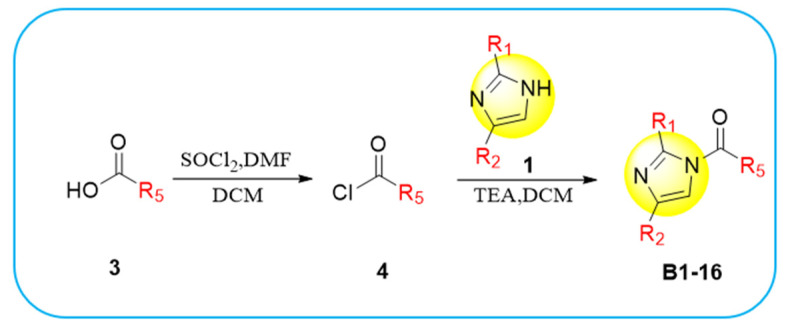
Synthesis plan for diimidazole skeleton derivatives **B1**–**16** (the yellow color highlights the imidazole moiety).

The synthesis of Compounds **B1**–**16** follows the general procedure outlined in Scheme 2. Compound **3** (0.58 mmol, 1 equiv) and SOCl_2_ (0.35 mL, 4.64 mmol, 8 equiv) are initially combined in a flask and dissolved in DCM. Subsequently, 3–5 drops of DMF are added dropwise at room temperature. The reaction is monitored using TLC. Once complete, the mixture is allowed to evaporate to dryness to yield Compound **4**.

Next, Compound **1** (0.50 mmol, 1 equiv) and Compound **4** (1.0 mmol, 2 equiv) are mixed in another flask and dissolved in DCM, followed by the addition of TEA (0.35 mL, 2.5 mmol, 5 equiv) at room temperature. The reaction is again monitored using TLC. After the reaction, the mixture is extracted with dichloromethane, washed with saturated sodium chloride solution, and the solvent is removed under vacuum. The desired compounds **B1**–**16** are then purified through column chromatography.

#### 2.5.3. Synthesis of Compounds **C1**–**7** (General Program)

**Scheme 3:**
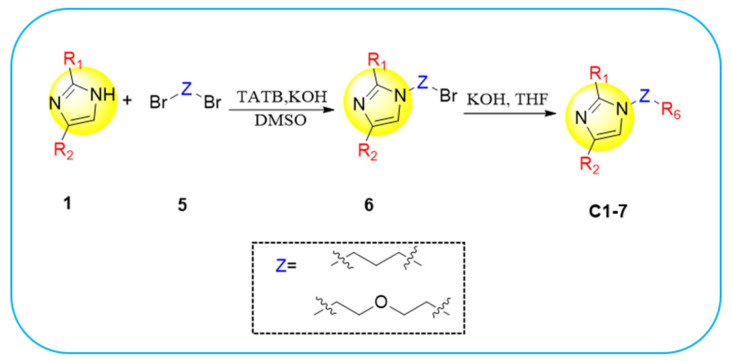
Synthesis plan for diimidazole skeleton derivatives **C1**–**7** (the yellow color highlights the imidazole moiety).

The synthesis of Compounds **C1**–**7** proceeds according to the general procedure outlined in Scheme 3. In a vial, Compound 1 (1.0 mmol, 1 equiv), Compound **5** (0.45 mmol, 0.45 equiv), and TABA (23.5 mg, 0.08 mmol, 0.08 equiv) are first dissolved in DMSO. Next, potassium hydroxide (56.0 mg, 1.0 mmol, 1 equiv) is added. The reaction mixture is heated to 70 °C and monitored using TLC for progress. Following completion, the mixture is extracted with dichloromethane, washed with saturated sodium chloride solution, and the solvent is removed under vacuum. The resulting compound, Compound **6**, is purified through column chromatography.

Compound **6** (0.30 mmol, 1 equiv) and pyrrolidine (0.30 mmol, 1 equiv) are introduced into a reaction vessel, dissolved in THF, and potassium hydroxide (16.8 mg, 0.30 mmol, 1 equiv) is added. The reaction is conducted at room temperature, with TLC used to monitor its progress. Following the reaction, the mixture is subjected to extraction with dichloromethane, washed with saturated sodium chloride solution, and the solvent is removed under vacuum. Finally, the compounds **C1**–**7** are isolated through column chromatography.

### 2.6. Anti-SARS-CoV-2 Nsp13 Helicase Activity

In an initial screening assay for the Nsp13 helicase inhibitory activity at a final concentration of 1 μM (Table S2), compounds **A16** and **B3** emerged with notable findings; they demonstrated an inhibition rate surpassing 50% against Nsp13. Further investigation led to the determination of their IC_50_ values, specifically for compound A16, which was found to be 1.25 μM, For compound **B3**, the IC_50_ was recorded at 0.98 μM (Table 1).

### 2.7. ADME Prediction

Compounds **A16** and **B3** were subjected to further evaluation for their drug-likeness and ADME properties. A comprehensive assessment was conducted on various physicochemical characteristics, including a molecular weight of approximately 500 Da, a partition coefficient (aQPlogPo/w) of five, exhibiting 10 hydrogen bond donors and acceptors, lipophilicity, solubility based on topological polar surface area (TPSA), consensus octanol–water partition coefficient (cLogP), and ESOL prediction of logD. The pharmacokinetic profile analysis revealed that both compounds adhered to Lipinski’s rule, suggesting acceptable gastrointestinal absorption within a physicochemically and pharmacokinetically reasonable range (Table 2). Consequently, these findings classify A16 and B3 as promising lead compounds with drug-like properties.

### 2.8. Molecular Docking Analysis

**Figure 7:**
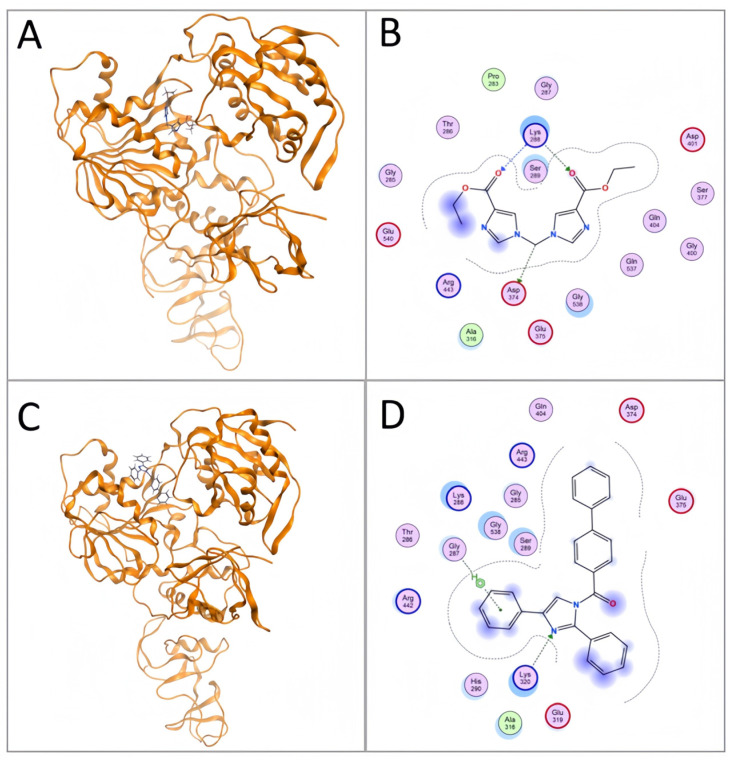
2D and 3D representations of compounds **A16** and **B3** at the SARS-CoV-2 Nsp13 binding site. (**A**,**B**) Docking model depicting simulated compound **A16** binding to Nsp13 (PDB: 6ZSL); (**C**,**D**) Docking model illustrating simulated compound **B3** binding to Nsp13 (PDB: 6ZSL).

Molecular docking studies demonstrated that Compounds **A16** and **B3** exhibited significant interactions with the amino acid residues at the SARS-CoV-2 Nsp13 helicase NTPase binding site (Figure 7 and Table 3). Compound **A16** formed electrostatic or hydrogen bonds with the carboxyl group at Lys288 and Asp374, with an affinity score of −7.1 kcal/mol. In contrast, **B3** interacted with the carboxyl groups at Gly287, Lys288, and Asp374, as well as with Arg442 and Arg443 through π–sigma interactions, with an affinity score of −8.7 kcal/mol. Notably, both **A16** and **B3** exhibited lower affinity scores than ADP, indicating a stronger affinity of these compounds towards the NTPase binding-site residues.

## 3. Discussion

This study employed a commercial fluorescent dye to label dsDNA substrates, generating fluorescence signals, and, based on the mechanism of Nsp13 helicase, developed a high-throughput fluorescence activity assay. Optimization of conditions determined the optimal dsDNA substrate concentration at 250 nM, Nsp13 enzyme at 25 nM, ATP at 2 mM, and a 60 min incubation period. Employing refined assay conditions, we conducted an IC_50_ assay for the reported Nsp13 helicase inhibitor, EA, which yielded an IC_50_ value of 0.67 µM. This result closely aligns with the previously reported FRET-measured IC_50_ of 1.50 µM for EA’s Nsp13 helicase inhibitory activity, substantiating the validity of our developed technique. The stability assessment demonstrated excellent performance, with a coefficient of variation (CV) of 2.43% (<10%) and a Z′ factor of 0.73 (>0.60), fulfilling the stringent requirements for high-throughput screening. Consequently, we have successfully established a robust high-throughput platform specifically designed for the study of Nsp13 helicase.

Utilizing the structural attributes of ADP, a structure-based design strategy was employed to synthesize three series of 40 imidazole-containing compounds: **A1**–**17** (yield 10–40%), **B1**–**B16** (yield 20–60%), and **C1**–**7** (yield 10–50%). All compounds were characterized by ^1^H NMR, ^13^C NMR, and HRMS (see Supplementary NMR spectra). The data confirmed the identity of the synthesized compounds with the target compound. The fluorescence assay revealed IC50 values of 1.25 μM and 0.98 μM for compounds **A16** and **B3**, respectively. According to Lipinski’s rule and the pharmacokinetic parameters, **A16** and **B3** showed potential drug-like properties. Molecular docking results showed that **A16** interacted with the carboxyl groups of Lys288 and Asp374, forming electrostatic or hydrogen bonds, while **B3** engaged with Gly287, Lys288, and Asp374 via similar interactions and formed π–sigma contacts with Arg442 and Arg443, enhancing their binding affinity with RecA1 and RecA2. Moreover, compared to ADP, **A16** and **B3** displayed stronger affinities with the NTPase binding-site residues, fulfilling the anticipated design goals.

## 4. Materials and Methods

### 4.1. SARS-CoV-2 Nsp13 Activity Detection

A fluorescence-based assay was ingeniously developed using the AccuBlue^®^ High Sensitivity dsDNA Quantitation Kit dye (Biotium, Fremont, CA, USA) for staining the dsDNA substrates and detecting fluorescence signals. This assay employs the principle of Nsp13 helicase activity to assess the in vitro functionality of SARS-CoV-2 Nsp13. To prepare the enzyme–substrate system, Short-strand DNA and overhang-strand DNA (Shanghai Sangon Bio, Shanghai, China) were mixed at a 1:1.1 ratio for dsDNA synthesis. Nsp13 was then introduced to interact with the dsDNA substrate, enabling it to unwind upon the enzyme’s action. Following this unwinding process, a fluorescent-dye mixture was added, and the subsequent fluorescence-intensity measurements served as a direct indicator of Nsp13’s enzymatic activity.

### 4.2. Condition Optimization

This study investigated the impact of varying conditions on dsDNA substrates, Nsp13 helicase, ATP, and incubation times, focusing on fluorescence-intensity changes under the dye. The optimal conditions were determined through methodological evaluation. A 5 μL of Nsp13 was incubated with 1 μL of compound (dissolved in DMSO) at room temperature for 10 min. Subsequently, 10 μL of dsDNA substrate and 5 μL of ATP were combined and incubated at 30 °C for 60 min. Following the reaction, 100 μL of the AccuBlue^®^ High Sensitivity dsDNA Quantitation Kit dye mixture was added, followed by a 10 min dark incubation at room temperature as per the manufacturer’s instructions. A volume of 115 μL was transferred to a black 96-well plate, and the fluorescence intensity was measured using a microplate reader (Thermo Fisher Scientific, Waltham, MA, USA, Varioskan LUX) with excitation at 485 nm and emission at 530 nm. Data analysis was performed using GraphPad Prism 9.0 software.

### 4.3. High-Throughput Screening Method

This research employed the optimized detection conditions to determine the IC_50_ of the reported Nsp13 helicase EA, aiming to validate the feasibility of the assay. Building upon the preliminary methodological assessment, high-throughput parameter optimization was conducted using dsDNA substrates, focusing on stability, coefficient of variation (CV), and Z′ factor. Rigorous criteria were set with a requirement for CV < 10% and Z′ > 0.60. EA served as the positive control, while DMSO acted as the negative control. Each experiment was repeated 24 times to measure the fluorescence intensity of the reaction mixture at various time points, ensuring stability within a defined time range. The CV and Z′ factor were calculated based on this data.

### 4.4. Chemistry

All the required chemicals and solvents were purchased from various commercial distributors and were pure. With thin-layer chromatography (DC-Fertigfolien Alugram (20 × 20 cm^2^) Kieselgel 60 F254 chromato plates, Guizhou Jiukai Technology Co., Ltd, Guizhou, China) the completion of each reaction was monitored and viewed under UV light. Impurities were purified using column chromatography and recrystallization techniques with suitable solvent systems. The structures of the synthesized compounds were characterized using various spectral techniques, like IR, ^1^H NMR, ^13^C NMR, and mass spectrometry.

### 4.5. ADME Prediction

Pharmacokinetic parameters are critical to ensuring the anticipated pharmacological properties of various biotransformation isomers. To forecast the pharmacokinetic parameters of the target compounds, we employed SWISS ADME, a web-based tool [15,16], which provides an extensive library of fast and dependable predictive models for physicochemical properties, pharmacokinetics, and drug likeness [17]. ADME stands for absorption (absorption), metabolism (metabolism), distribution (distribution), and excretion (excretion). Using the SWISS ADME software, the pharmacokinetic characteristics of the compound **A16** and **B3** were assessed (http://www.swissadme.ch) on 14 April 2024. Their wide molecular profiles, including their physicochemical characteristics, pharmacokinetics, solubility, lipophilicity, and drug-likeness, were examined.

According to Lipinski’s rule, a molecule should have a molecular weight (MW) below 500, a partition coefficient (clogP) less than 5, not more than 5 hydrogen bond donors (HBD), and no more than 10 hydrogen bond acceptors (HBA) for favorable oral absorption; approximately 90% of orally bioavailable compounds adhere to these guidelines. Conversely, compounds violating two or more of these criteria generally have a lower likelihood of becoming therapeutically viable.

### 4.6. Molecular Docking

The 6ZSL protein crystal structure was obtained from the Protein Data Bank (PDB) with identifier 6ZSL, and the 3D structure of the small molecule was downloaded from the PUBCHEM database, followed by energy minimization under the MMFF94 force field. This study employed AutoDock Vina 1.1.2 software [18] for molecular docking purposes. Prior to docking, PyMol 2.5.5 [19] was used to preprocess the receptor protein by eliminating water molecules, salt ions, and the small molecule. A docking box was then established to encompass the complete protein structure. ADFRsuite 1.0 [20] was utilized to convert both the prepared ligands and receptor into the necessary PDBQT format that is compatible with AutoDock Vina 1.1.2. The docking run employed a global search resolution of 32 while keeping all other parameters at their default values. The highest-scoring docked complex was deemed the binding conformation, and finally, the results from Moe 2019 were analyzed visually.

## 5. Conclusions

Given the severe health implications and mortality risks posed by SARS-CoV-2, current research efforts focus on designing novel imidazole-containing compounds as potential antiviral agents against the virus. This work initially employed a commercial fluorescent dye to stain dsDNA substrates, generating fluorescence signals. Leveraging the mechanism of Nsp13 helicase, a fluorescence-based detection method was designed. The dye stains dsDNA, producing fluorescence, which disappears upon unwinding by the enzyme. An optimized and high-throughput screening method was established through parameter optimization and high-throughput measurements. Compounds **A16** and **B3** demonstrated IC_50_ values of 1.25 μM and 0.98 μM against Nsp13 helicase using the developed in vitro fluorescence assay. According to Lipinski’s rule and the pharmacokinetic parameters, **A16** and **B3** exhibit drug-like properties. Molecular docking revealed that **A16** forms electrostatic interactions or hydrogen bonds with carboxyl groups at Lys288 and Asp374, while **B3** interacts with Gly287, Lys288, and Asp374, and engages in π–sigma interactions with Arg442 and Arg443, enhancing binding to the RecA1 and RecA2 key regions. Additionally, compounds A16 and B3 exhibit stronger affinity for the NTPase binding site compared to ADP, fulfilling the intended design. In conclusion, compounds A16 and B3 hold promise as antiviral lead compounds, suitable for further targeted drug-development studies.

## Figures and Tables

**Table 1:** IC_50_ values of compounds **A16** and **B3**.

Compound	Nsp13 IC_50_ ± SD (μM)
**A16**	1.25 ± 0.03
**B3**	0.98 ± 0.1
**Ellagic acid**	0.67 ± 0.23

**Table 2:** Pharmacokinetics (ADME) spectrum of predictive compounds **A16** and **B3**.

Molecule	MW	HBA	HBD	TPSA	Consensus Log P	Silicos-IT LogSw	GI Absorption	BBB Permeant	Lipinski #Violations
**A16**	292.29	6	0	88.24	0.84	−2.30	High	No	0
**B3**	400.47	2	0	34.89	5.64	−10.35	High	No	1
**EA**	302.19	8	4	141.34	0.95	−3.35	High	No	0

**Table 3:** Affinity scores (Kcal/Mol) and interaction with amino acids in the SARS-CoV-2 Nsp13 ATPase pocket.

Compounds	Affinity Score (kcal/mol)	Interacted with
**A16**	−7.1	Lys288, Asp374
**B3**	−8.7	Gly287, Lys288, Asp374, Arg442, Arg442
**ADP**	−6.5	Lys288, Asp374, Glu375, Arg567

## Data Availability

The data presented in this study are available in article and Supplementary Materials.

## Supplementary Materials

The following supporting information can be downloaded at: https://www.mdpi.com/article/10.3390/molecules29102301/s1, Figure S1: DNA agar sugar gel electrophoresis; Table S1: Synthesized imidazole-containing scaffolding compounds; Table S2: Preliminarily screening against Nsp13 helicase activity for 40 imidazole-containing compounds; Characterization of synthesized compounds, Spectrums of ^13^C NMR and ^1^H NMR for synthesized compounds.
