# Supplementary material for: Development of a Fluorescent Assay and Imidazole-Containing Inhibitors by Targeting SARS-CoV-2 Nsp13 Helicase
Source: Molecules. 2024 May 14;29(10):2301. doi: 10.3390/molecules29102301 (PMC11124022; doi:10.3390/molecules29102301)

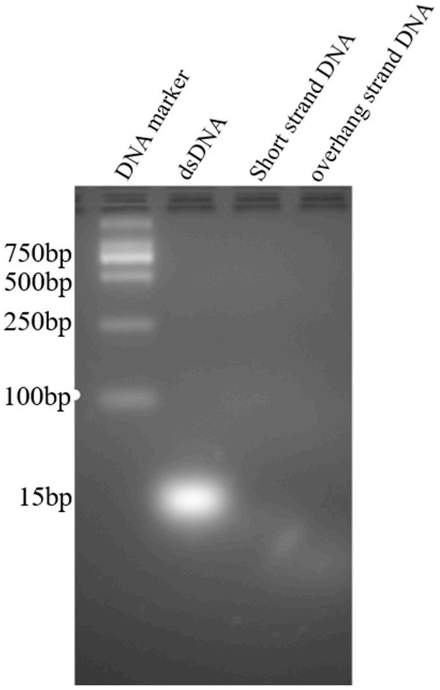

Figure S1. DNA agar sugar gel electrophoresis

Table S1.40 Synthesized imidazole-containing scaffolding compounds

S. No.	Molecule ID	Structure	S. No.	Molecule ID	Structure
1	A1		21	B4	
2	A2		22	B5	
3	A3		23	B6	
4	A4		24	B7	
5	A5		25	B8	

**6**	A6	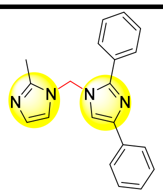	**26**	**B9**	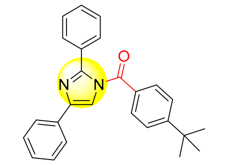
**7**	A7	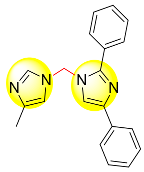	**27**	**B10**	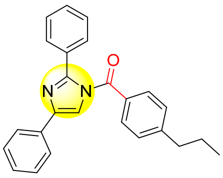
**8**	A8	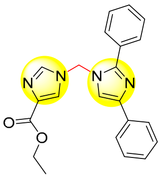	**28**	**B11**	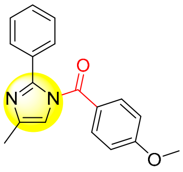
**9**	A9	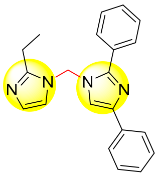	**29**	**B12**	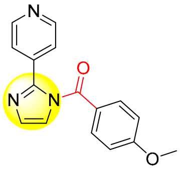
**10**	A10	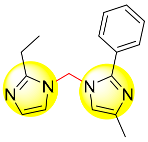	**30**	**B13**	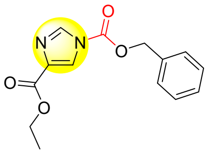
**11**	A11	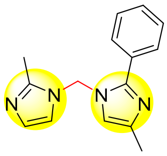	**31**	**B14**	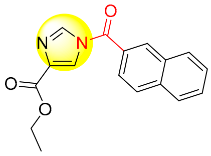
**12**	A12	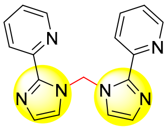	**32**	**B15**	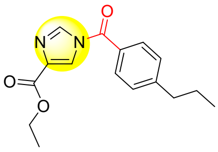
**13**	A13	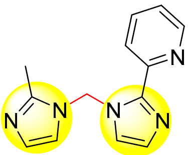	**33**	**B16**	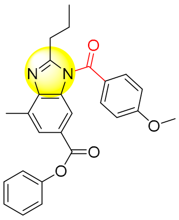
**14**	A14	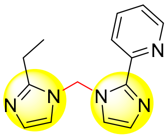	**34**	**C1**	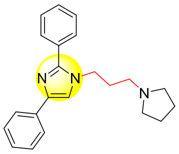
**15**	A15	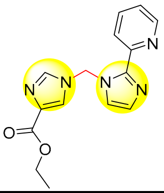	**35**	**C2**	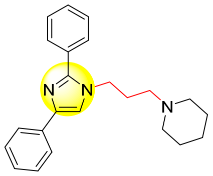

**16**	A16	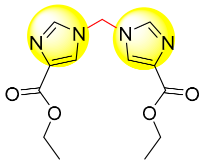	**36**	**C3**	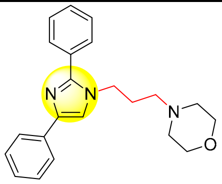
**17**	**A17**	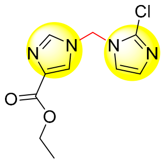	**37**	**C4**	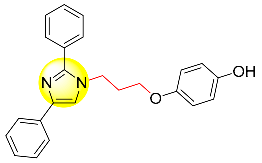
**18**	**B1**	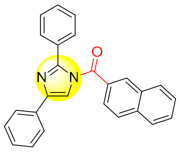	**38**	**C5**	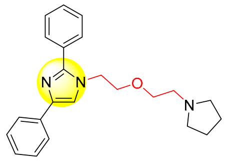
**19**	**B2**	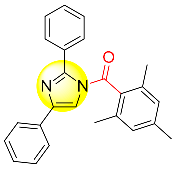	**39**	**C6**	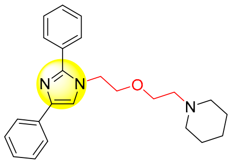
**20**	**B3**	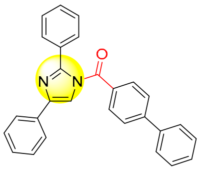	**40**	**C7**	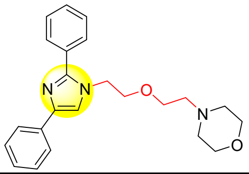

Table S2. preliminarily screened 40 imidazole-containing compounds for in vitro Nsp13 helicase inhibitory activity

Compound	Nsp13 (inhib%)±SD	Compound	Nsp13 (inhib%)±SD
	1 μM		1 μM
**A1**	29.13±0.16	**B5**	4.80±0.04
**A2**	22.00±0.01	**B6**	23.17±0.07
**A3**	12.78±0.05	**B7**	36.67±0.06
**A4**	13.94±0.06	**B8**	21.36±0.02
**A5**	35.08±0.05	**B9**	28.54±0.02
**A6**	22.27±0.01	**B10**	23.21±0.01
**A7**	27.86±0.03	**B11**	43.18±0.01
**A8**	26.53±0.03	**B12**	5.31±0.03
**A9**	9.33±0.05	**B13**	19.27±0.06
**A10**	13.19±0.09	**B14**	28.12±0.05
**A11**	4.84±0.03	**B15**	18.37±0.04
**A12**	6.22±0.05	**B16**	28.09±0.09
**A13**	25.73±0.01	**C1**	16.73±0.1
**A14**	22.73±0.01	**C2**	23.00±0.01
**A15**	19.62±0.07	**C3**	24.39±0.02
**A16**	58.86±0.08	**C4**	22.53±0.12
**A17**	18.63±0.06	**C5**	7.97±0.05
**B1**	21.33±0.07	**C6**	32.16±0.13
**B2**	34.80±0.17	**C7**	22.92±0.09
**B3**	51.16±0.10	**EA**	95.44±0.01
**B4**	22.19±0.16		

Characterization of Synthesized Compounds
All synthetic imidazole-containing backbone derivatives were characterized by NMR spectroscopy and mass spectrometry.

bis(2,4-diphenyl-1H-imidazol-1-yl)methane（A1）： White solid; Yield: 19.5%;^{1}H NMR (400 MHz, $CDCl_3$) δ 8.02 (d, *J* = 7.3 Hz, 4H), 7.74 – 7.56 (m, 16H), 7.51 (t, *J* = 7.4 Hz, 2H), 6.22 (s, 2H). ^{13}C NMR (101 MHz, $CDCl_3$) δ 148.26, 142.40, 133.26, 129.86, 129.50, 129.19, 129.15, 128.75, 127.49, 125.14, 114.83, 56.73. HRMS (ESI) *m/z* calculated for $C_{31}H_{25}N_4$ $[M+H]^+$ 453.2074, found 453.2060.

bis(2-phenyl-1H-imidazol-1-yl)methane（A2）： White solid; Yield:15.5%;^{1}H NMR (400 MHz, $CDCl_3$) δ 7.43 – 7.36 (m, 10H), 7.11 (d, *J* = 1.5 Hz, 2H), 6.80 (d, *J* = 1.4 Hz, 2H), 6.10 (s, 2H). ^{13}C NMR (101 MHz, $CDCl_3$) δ 129.91, 129.64, 129.59, 129.01, 128.97, 119.43. HRMS(ESI)m/z calculated for C19H17N4 $[M+H]^+$ 301.1447, found 301.1439. HRMS (ESI) *m/z* calculated for $C_{19}H_{17}N_4$ $[M+H]^+$ 301.1448, found 301.1441.

bis(4-methyl-2-phenyl-1H-imidazol-1-yl)methane(A3): White solid; Yield:21.5%; ^{1}H NMR (400 MHz, $CDCl_3$) δ 7.39 (d, *J* = 15.5 Hz, 10H), 6.55 (s, 2H), 5.96 (s, 2H), 2.21 (s, 6H).^{13}C NMR (101 MHz, $CDCl_3$) δ 147.28, 138.91, 129.43, 128.94, 128.88, 115.76, 56.34, 13.74. HRMS (ESI) *m/z* calculated for $C_{21}H_{22}N_4$ $[M+H]^+$ 329.1760, found 329.1753.

1-((2,4-diphenyl-1H-imidazol-1-yl)methyl)-4-methyl-2-phenyl-1H-imidazole (A4): White solid; Yield: 12.7%;^{1}H NMR (400 MHz, $CDCl_3$) δ 7.75 – 7.72 (m, 2H), 7.50 – 7.48 (m, 2H), 7.45 (dd, *J* = 7.1, 2.8 Hz, 4H), 7.32 (d, *J* = 7.5 Hz, 3H), 7.19 (s, 1H), 6.45 (s, 1H), 5.88 (s, 2H), 2.09 (s, 3H). ^{13}C NMR (101 MHz, $CDCl_3$) δ 148.43, 142.56, 135.76, 133.27, 129.98, 129.41, 129.21, 128.73, 127.49, 125.19, 115.16, 114.31, 56.44, 29.78, 13.59. HRMS (ESI) *m/z* calculated for $C_{26}H_{23}N_4$ $[M+H]^+$ 391.1917, found 391.1910.

2,4-diphenyl-1-((2-phenyl-1H-imidazol-1-yl)methyl)-1H-imidazole(A5): White solid; Yield: 18.5%; ^{1}H NMR (400 MHz, $CDCl_3$) δ 7.77 (d, *J* = 7.1 Hz, 2H), 7.48 – 7.42 (m, 10H), 7.39 (t, *J* = 7.6 Hz, 2H), 7.30 – 7.27 (m, 1H), 7.03 (s, 1H), 6.87 (s, 1H), 6.11 (s, 2H).^{13}C NMR (101 MHz, $CDCl_3$) δ 148.25, 148.02, 142.42, 133.30, 129.88, 129.78, 129.69, 129.58, 129.49, 129.13, 129.06, 129.02, 128.71, 127.45, 125.13, 119.

56, 114.72, 56.66, 29.78, 1.12. HRMS (ESI) *m/z* calculated for $C_{25}H_{20}N_4$ $[M+H]^+$ 377.1761, found 377.1756.

1-((2-methyl-1H-imidazol-1-yl)methyl)-2,4-diphenyl-1H-imidazole(A6): White solid; Yield: 12.2%; ^1H NMR (400 MHz, $CDCl_3$) δ 7.84 (d, *J* = 6.9 Hz, 2H), 7.63 – 7.53 (m, 6H), 7.41 (t, *J* = 7.7 Hz, 2H), 7.33 – 7.30 (m, 1H), 6.94 (d, *J* = 1.5 Hz, 1H), 6.77 (d, *J* = 1.4 Hz, 1H), 5.99 (s, 2H), 2.21 (s, 3H). ^{13}C NMR (101 MHz, $CDCl_3$) δ 148.40, 144.50, 142.62, 133.28, 129.97, 129.70, 129.39, 129.21, 128.73, 128.50, 127.50, 125.18, 118.58, 114.63, 55.88, 29.78, 12.89. HRMS (ESI) *m/z* calculated for $C_{20}H_{19}N_4$ $[M+H]^+$ 315.1604, found 315.1600.

1-((4-methyl-1H-imidazol-1-yl)methyl)-2,4-diphenyl-1H-imidazole(A7): White solid; Yield: 14.3%; ^1H NMR (400 MHz, $CDCl_3$) δ 7.74 (d, *J* = 7.0 Hz, 2H), 7.50 – 7.48 (m, 2H), 7.45 (dd, *J* = 7.1, 2.8 Hz, 4H), 7.32 (d, *J* = 7.5 Hz, 3H), 7.19 (s, 1H), 6.45 (s, 1H), 5.88 (s, 2H), 2.09 (s, 3H). ^{13}C NMR (101 MHz, $CDCl_3$) δ 148.43, 142.56, 135.76, 133.27, 129.98, 129.41, 129.21, 128.73, 127.49, 125.19, 115.16, 114.31, 29.78, 13.59. HRMS (ESI) *m/z* calculated for $C_{20}H_{18}N_4$ $[M+H]^+$ 315.1604, found 315.1599.

ethyl1-((2,4-diphenyl-1H-imidazol-1-yl)methyl)-1H-imidazole-4-carboxylate (A8): White solid; Yield: 8.0%; ^1H NMR (400 MHz, $CDCl_3$) δ 7.82 – 7.79 (m, 2H), 7.68 (s, 1H), 7.58 – 7.52 (m, 6H), 7.37 (t, *J* = 7.6 Hz, 3H), 6.96 (s, 1H), 6.51 (s, 2H), 4.39 – 4.32 (m, 2H), 1.38 (t, *J* = 7.1 Hz, 3H). ^{13}C NMR (101 MHz, $CDCl_3$) δ 160.63, 148.36, 141.94, 141.31, 138.16, 133.37, 130.12, 129.78, 129.53, 129.40, 128.72, 127.39, 125.17, 121.95, 116.19, 61.25, 54.92, 29.81, 14.38. HRMS (ESI) *m/z* calculated for $C_{22}H_{21}N_4O_2$ $[M+H]^+$ 373.1659, found 373.1650.

1-((2-ethyl-1H-imidazol-1-yl)methyl)-2,4-diphenyl-1H-imidazole(A9): White solid; Yield: 17.3%; ^1H NMR (400 MHz, $CDCl_3$) δ 7.80 (d, *J* = 7.8 Hz, 2H), 7.62 – 7.48 (m, 6H), 7.38 (t, *J* = 7.4 Hz, 2H), 7.27 (d, *J* = 7.3 Hz, 1H), 6.95 (s, 1H), 6.75 (s, 1H), 5.95 (s, 2H), 2.42 (q, *J* = 7.4 Hz, 2H), 1.19 (t, *J* = 7.5 Hz, 3H). ^{13}C NMR (101 MHz, $CDCl_3$) δ 149.19, 148.37, 142.62, 133.30, 129.95, 129.69, 129.34, 129.19, 128.73, 128.41, 127.49, 125.18, 118.55, 114.58, 55.59, 29.78, 20.01, 11.82. HRMS (ESI) *m/z* calculated for $C_{21}H_{21}N_4$ $[M+H]^+$ 329.1761, found 329.1758.

1-((2-ethyl-1H-imidazol-1-yl)methyl)-4-methyl-2-phenyl-1H-imidazole(A10): White solid; Yield: 9.0%; ^{1}H NMR (400 MHz, $CDCl_3$) δ 7.46 (d, *J* = 1.9 Hz, 5H), 6.91 (d, *J* = 1.7 Hz, 1H), 6.70 – 6.64 (m, 2H), 5.88 (s, 2H), 2.39 (q, *J* = 7.6 Hz, 2H), 2.22 (s, 3H), 1.16 (t, *J* = 7.5 Hz, 3H). ^{13}C NMR (101 MHz, $CDCl_3$) δ 149.10, 147.46, 139.09, 129.86, 129.63, 129.09, 129.06, 128.35, 118.49, 115.70, 55.30, 20.00, 13.68, 11.81. HRMS (ESI) *m/z* calculated for $C_{16}H_{19}N_4$ [M+H]$^+$ 267.1604,found 267.1600.

4-methyl-1-((2-methyl-1H-imidazol-1-yl)methyl)-2-phenyl-1H-imidazole(A11): White solid; Yield: 11.3%; ^{1}H NMR (400 MHz, $CDCl_3$) δ 7.48 (d, J = 1.9 Hz, 5H), 6.88 (d, J = 1.3 Hz, 1H), 6.68 (t, J = 1.1 Hz, 2H), 5.88 (s, 2H), 2.24 (s, 3H), 2.14 (s, 3H).^{13}C NMR (101 MHz, $CDCl_3$) δ 139.14, 129.71, 129.17, 129.12, 128.99, 128.35, 118.58, 115.77, 55.61, 29.81, 13.74, 12.94. HRMS (ESI) *m/z* calculated for $C_{15}H_{17}N_4$ [M+H]$^+$ 253.1448, found 253.1446.

bis(2-(pyridin-2-yl)-1H-imidazol-1-yl)methane（A12）: White solid; Yield: 10%; ^{1}H NMR (400 MHz, $CDCl_3$) δ 8.55 (d, *J* = 4.9 Hz, 2H), 8.24 (d, *J* = 8.0 Hz, 2H), 7.80 (td, *J* = 7.8, 1.8 Hz, 2H), 7.68 (s, 2H), 7.37 (d, *J* = 1.3 Hz, 2H), 7.31 – 7.27 (m, 2H), 7.06 (s, 2H). ^{13}C NMR (101 MHz, $CDCl_3$) δ 150.42, 148.23, 144.12, 137.25, 129.23, 123.19, 122.67, 56.81. HRMS (ESI) *m/z* calculated for $C_{17}H_{15}N_6$ [M+H]$^+$ 303.1353, found 303.1346.

2-(1-((2-methyl-1H-imidazol-1-yl)methyl)-1H-imidazol-2-yl)pyridine(A13): White solid; Yield: 13.3%;^{1}H NMR (400 MHz, $CDCl_3$) δ 8.58 (dd, *J* = 5.0, 1.1 Hz, 1H), 8.22 (d, *J* = 7.8 Hz, 1H), 7.80 (td, *J* = 7.9, 1.9 Hz, 1H), 7.28 (ddd, *J* = 7.8, 5.0, 1.4 Hz, 1H), 7.14 (d, *J* = 1.3 Hz, 1H), 6.99 (d, *J* = 1.5 Hz, 1H), 6.93 (d, *J* = 1.3 Hz, 1H), 6.89 (d, *J* = 1.5 Hz, 1H), 6.80 (s, 2H), 2.44 (s, 3H). 13C NMR (101 MHz, $CDCl_3$) δ 150.04, 148.25, 144.93, 144.18, 137.39, 129.78, 127.35, 123.48, 123.07, 121.62, 119.32, 56.81, 29.81. HRMS (ESI) *m/z* calculated for $C_{13}H_{14}N_5$ [M+H]$^+$ 240.1244, found 240.1244.

2-(1-((2-ethyl-1H-imidazol-1-yl)methyl)-1H-imidazol-2-yl)pyridine(A14): White solid; Yield: 11.4%; ^{1}H NMR (400 MHz, $CDCl_3$) δ 8.59 (ddd, *J* = 4.9, 1.8, 0.9 Hz, 1H), 8.24 (dt, *J* = 8.1, 1.1 Hz, 1H), 7.80 (td, *J* = 7.8, 1.8 Hz, 1H), 7.29 (ddd, *J* = 7.5, 4.9, 1.2 Hz, 1H), 7.14 (d, *J* = 1.3 Hz, 1H), 6.98 (d, *J* = 1.5 Hz, 1H), 6.95 (d, *J* = 1.5 Hz,

1H), 6.88 (d, *J* = 1.3 Hz, 1H), 6.80 (s, 2H), 2.73 (q, *J* = 7.5 Hz, 2H), 1.28 – 1.26 (m, 3 H). ^{13}C NMR (101 MHz, $CDCl_3$) δ 150.10, 149.67, 148.26, 144.19, 137.38, 129.77, 12 7.67, 123.44, 123.07, 121.41, 119.25, 56.55, 29.82, 20.14, 12.10. HRMS (ESI) *m/z* cal culated for $C_{14}H_{16}N_5$ $[M+H]^+$ 254.1400, found 254.1398.

ethyl1-((2-(pyridin-2-yl)-1H-imidazol-1-yl)methyl)-1H-imidazole-4-carboxyl ate(A15): White solid; Yield: 7.5%; ^{1}H NMR (400 MHz, $CDCl_3$) δ 8.61 (d, *J* = 5.0 H z, 1H), 8.24 (dq, *J* = 8.1, 1.2 Hz, 1H), 7.82 (d, *J* = 1.4 Hz, 2H), 7.79 (dd, *J* = 7.9, 1.8 H z, 1H), 7.30 (ddd, *J* = 7.6, 4.9, 1.2 Hz, 1H), 7.18 (d, *J* = 1.3 Hz, 1H), 7.15 (d, *J* = 1.2 H z, 1H), 6.86 (s, 2H), 4.32 (q, *J* = 7.1 Hz, 2H), 1.33 (t, *J* = 7.1 Hz, 3H).^{13}C NMR (101 MHz, $CDCl_3$) δ 162.65, 149.71, 148.19, 143.96, 137.85, 137.42, 134.79, 130.04, 124. 45, 123.61, 122.99, 122.51, 60.76, 57.21, 14.40. HRMS (ESI) *m/z* calculated for $C_{15}H_{16}N_5O_2$ $[M+H]^+$ 298.1299, found 298.1292.

diethyl 1,1'-methylenebis(1H-imidazole-4-carboxylate)(A16): White solid; Y ield: 43.6%;^{1}H NMR (400 MHz, $CDCl_3$) δ 8.17 (d, *J* = 0.8 Hz, 2H), 7.65 (d, *J* = 0.9 H z, 2H), 6.82 (s, 2H), 4.30 (q, *J* = 7.1 Hz, 4H), 1.32 (t, *J* = 7.1 Hz, 6H).^{13}C NMR (101 MHz, $CDCl_3$) δ 160.86, 143.46, 138.24, 121.66, 61.12, 52.79, 14.26. IR(KBr): 3132.4 0 cm^{-1}(=C-H str, m); 1716.65 cm^{-1} (C=O, s); 1543.05 cm^{-1} (Ar C=C str, m); and 112 6.43 cm^{-1} (C-O-C). HRMS (ESI) *m/z* calculated for $C_{13}H_{17}N_4O_4$ $[M+H]^+$ 293.1244, fo und 293.1237.

ethyl1-((2-chloro-1H-imidazol-1-yl)methyl)-1H-imidazole-4-carboxylate（A 17）: White solid; Yield: 10%; ^{1}H NMR (400 MHz, $CDCl_3$) δ 7.88 (s, 1H), 7.69 (s, 1 H), 7.30 (d, *J* = 1.6 Hz, 1H), 6.87 (d, *J* = 1.7 Hz, 1H), 6.38 (s, 2H), 4.32 (q, *J* = 7.1 Hz, 2H), 1.34 (t, *J* = 7.1 Hz, 3H).^{13}C NMR (101 MHz, $CDCl_3$) δ 160.61, 142.18, 138.29, 128.95, 121.89, 61.27, 53.80, 14.30. HRMS (ESI) *m/z* calculated for $C_{10}H_{12}ClN_4O_2$ $[M+H]^+$ 255.0643, found 255.0639.

(2,4-diphenyl-1H-imidazol-1-yl)(naphthalen-2-yl)methanone(B1): White soli d; Yield: 93%; ^{1}H NMR (400 MHz, $CDCl_3$) δ 8.36 (s, 1H), 7.94 (d, *J* = 7.7 Hz, 2H), 7. 89 (d, *J* = 7.6 Hz, 4H), 7.67 (d, *J* = 8.7 Hz, 4H), 7.58 (t, *J* = 7.4 Hz, 1H), 7.44 (t, *J* = 7. 5 Hz, 2H), 7.32 (dd, *J* = 17.1, 7.7 Hz, 4H).^{13}C NMR (101 MHz, $CDCl_3$) δ 168.08, 150. 13, 141.64, 135.91, 133.03, 132.88, 132.14, 130.74, 129.65, 129.56, 129.34, 129.26, 1

29.10, 128.98, 128.89, 128.43, 128.06, 127.94, 127.54, 125.61, 116.55. HRMS (ESI) *m/z* calculated for $C_{26}H_{19}N_2O$ [M+H]$^+$ 375.1492, found 375.1487.

(2,4-diphenyl-1H-imidazol-1-yl)(mesityl)methanone(B2): White solid; Yield: 74.2%; ^{1}H NMR (400 MHz, $CDCl_3$) δ 7.69 (dd, *J* = 6.2, 2.7 Hz, 2H), 7.44 – 7.38 (m, 6 H), 7.32 (t, *J* = 7.4 Hz, 1H), 6.87 (s, 2H), 2.32 (s, 3H), 2.28 (s, 6H). ^{13}C NMR (101 M Hz, $CDCl_3$) δ 168.33, 149.56, 142.01, 140.79, 135.25, 132.55, 131.48, 131.30, 129.44, 129.36, 128.97, 128.81, 128.07, 128.03, 125.59, 114.36, 21.37, 19.65. HRMS (ESI) *m /z* calculated for $C_{25}H_{23}N_2O$ [M+H]$^+$ 367.1805, found 367.1801.

[1,1'-biphenyl]-4-yl(2,4-diphenyl-1H-imidazol-1-yl)methanone(B3): White so lid; Yield: 25.6%; ^{1}H NMR (400 MHz, $CDCl_3$) δ 8.01 – 7.87 (m, 4H), 7.68 (d, *J* = 1.4 Hz, 2H), 7.66 – 7.59 (m, 5H), 7.54 – 7.48 (m, 2H), 7.48 – 7.42 (m, 3H), 7.35 (dd, *J* = 6.4, 2.1 Hz, 4H).^{13}C NMR (101 MHz, $CDCl_3$) δ 167.71, 150.02, 147.04, 141.64, 139.3 9, 132.85, 131.46, 130.69, 130.63, 129.37, 129.26, 129.05, 128.87, 128.44, 127.93, 12 7.50, 127.46, 125.60, 116.35. IR(KBr): 3363.86 cm^{-1}(=C-H str, m); 1720.50 cm^{-1} (C= O, s); and 1604.77 cm^{-1} (Ar C=C str, m). HRMS (ESI) *m/z* calculated for $C_{28}H_{21}N_2O$ [M+H]$^+$ 401.1648, found 401.1645.

(2,4-diphenyl-1H-imidazol-1-yl)(4-methoxyphenyl)methanone(B4): White sol id; Yield: 52%; ^{1}H NMR (400 MHz, $CDCl_3$) δ 7.91 (d, *J* = 7.6 Hz, 2H), 7.80 (d, *J* = 8. 6 Hz, 2H), 7.61 (d, *J* = 7.7 Hz, 3H), 7.42 (t, *J* = 7.5 Hz, 2H), 7.31 (d, *J* = 6.7 Hz, 4H), 6.90 (d, *J* = 8.6 Hz, 2H), 3.80 (s, 3H).^{13}C NMR (101 MHz, $CDCl_3$) δ 167.25, 164.64, 149.71, 141.42, 133.40, 133.01, 130.69, 129.25, 128.86, 128.83, 128.43, 127.78, 125. 50, 124.04, 116.54, 114.32, 55.80. HRMS (ESI) *m/z* calculated for $C_{23}H_{19}N_2O_2$[M+H]$^+$ 355.1441, found 355.1440.

(2,4-diphenyl-1H-imidazol-1-yl)(furan-2-yl)methanone(B5): White solid; Yiel d: 16.2%; ^{1}H NMR (400 MHz, $CDCl_3$) δ 7.91 (d, *J* = 7.8 Hz, 2H), 7.84 (s, 1H), 7.61 (d, *J* = 5.9 Hz, 3H), 7.43 (t, *J* = 7.6 Hz, 2H), 7.39 – 7.32 (m, 5H), 6.56 (dd, *J* = 3.3, 1.5 Hz, 1H).^{13}C NMR (101 MHz, $CDCl_3$) δ 156.33, 149.61, 148.48, 145.87, 141.93, 132. 75, 130.83, 129.32, 128.82, 128.72, 128.46, 127.92, 125.57, 123.26, 115.54, 113.20. H RMS (ESI) *m/z* calculated for $C_{20}H_{15}N_2O_2$[M+H]$^+$ 315.1128, found 315.1125.

(2,4-diphenyl-1H-imidazol-1-yl)(3,4,5-trimethoxyphenyl)methanone(B6): White solid; Yield: 7.0%; ^{1}H NMR (400 MHz, $CDCl_3$) δ 7.92 – 7.87 (m, 2H), 7.68 (s, 1H), 7.59 – 7.54 (m, 2H), 7.42 (t, *J* = 7.6 Hz, 2H), 7.32 (dq, *J* = 4.6, 2.7, 1.8 Hz, 4H), 7.03 (s, 2H), 3.90 (s, 3H), 3.83 (s, 6H).^{13}C NMR (101 MHz, $CDCl_3$) δ 167.44, 153.11, 149.80, 143.36, 141.66, 132.75, 130.66, 129.35, 128.84, 128.80, 128.40, 127.91, 126.66, 125.50, 116.32, 108.26, 61.15, 56.47. HRMS (ESI) *m/z* calculated for $C_{25}H_{23}N_2O_4$ $[M+H]^+$ 415.1652, found 415.1651.

cyclopropyl(2,4-diphenyl-1H-imidazol-1-yl)methanone(B7): White solid; Yield: 46%; ^{1}H NMR (400 MHz, $CDCl_3$) δ 7.79 (d, *J* = 6.4 Hz, 2H), 7.54 – 7.46 (m, 2H), 7.27 – 7.07 (m, 6H), 6.97 (s, 1H), 1.59 (tt, *J* = 8.3, 4.6 Hz, 1H), 0.99 – 0.94 (m, 2H), 0.80 – 0.74 (m, 2H). ^{13}C NMR (101 MHz, $CDCl_3$) δ 180.83, 146.14, 135.88, 129.81, 129.43, 128.72, 128.70, 127.87, 126.52, 126.33, 125.33, 117.37, 14.13, 8.28. HRMS (ESI) *m/z* calculated for $C_{19}H_{17}N_2O$ $[M+H]^+$ 289.1335, found 289.1335.

benzyl 2,4-diphenyl-1H-imidazole-1-carboxylate(B8): White solid; Yield: 5.1%; ^{1}H NMR (400 MHz, $CDCl_3$) δ 7.84 (d, *J* = 7.1 Hz, 2H), 7.79 (s, 1H), 7.64 – 7.56 (m, 2H), 7.46 – 7.32 (m, 8H), 7.30 – 7.21 (m, 3H), 5.28 (s, 2H). ^{13}C NMR (101 MHz, $CDCl_3$) δ 149.56, 149.11, 141.45, 133.92, 132.58, 131.29, 129.66, 129.43, 129.01, 128.76, 128.68, 128.66, 127.85, 127.81, 125.47, 114.27, 69.79.HRMS (ESI) *m/z* calculated for $C_{23}H_{19}N_2O_2$ $[M+H]^+$ 355.1441, found 355.1438.

(4-(tert-butyl)phenyl)(2,4-diphenyl-1H-imidazol-1-yl)methanone(B9): White solid; Yield: 24.5%; ^{1}H NMR (400 MHz, $CDCl_3$) δ 7.90 (d, *J* = 6.9 Hz, 2H), 7.78 (d, *J* = 8.5 Hz, 2H), 7.62 (s, 1H), 7.58 (dd, *J* = 6.6, 3.0 Hz, 2H), 7.43 (dd, *J* = 22.8, 8.1 Hz, 4H), 7.35 – 7.28 (m, 4H), 1.34 (s, 9H). ^{13}C NMR (101 MHz, $CDCl_3$) δ 167.66, 158.34, 149.96, 141.41, 132.82, 130.75, 129.11, 128.92, 128.70, 128.21, 127.73, 125.83, 125.49, 116.24, 35.33, 31.00. HRMS (ESI) *m/z* calculated for $C_{26}H_{25}N_2O$ $[M+H]^+$ 381.1961, found 381.1958.

(2,4-diphenyl-1H-imidazol-1-yl)(4-propylphenyl)methanone(B10): White solid; Yield: 40.2%; ^{1}H NMR (400 MHz, $CDCl_3$) δ 7.66 (d, *J* = 8.0 Hz, 2H), 7.49 (d, *J* = 7.6 Hz, 2H), 7.36 (d, *J* = 8.9 Hz, 3H), 7.16 (t, *J* = 7.7 Hz, 2H), 7.11 – 7.01 (m, 4H), 6.98 (d, *J* = 8.3 Hz, 2H), 2.38 (t, *J* = 7.6 Hz, 2H), 1.39 (q, *J* = 7.5 Hz, 2H), 0.68 (t, *J* = 7.

3 Hz, 3H). ^{13}C NMR (101 MHz, $CDCl_3$) δ 167.89, 150.18, 149.93, 141.49, 132.97, 131.01, 130.79, 129.52, 129.22, 129.07, 129.00, 128.82, 128.35, 127.82, 125.56, 116.43, 38.15, 24.29, 13.84. HRMS (ESI) *m/z* calculated for $C_{25}H_{23}N_2O$ $[M+H]^+$ 367.1805, found 367.1806.

(4-methoxyphenyl)(4-methyl-2-phenyl-1H-imidazol-1-yl)methanone(B11): White solid; Yield: 5.3%; ^1H NMR (400 MHz, $CDCl_3$) δ 7.72 (d, *J* = 6.6 Hz, 2H), 7.47 (d, *J* = 3.2 Hz, 2H), 7.23 (s, 3H), 6.99 (s, 1H), 6.84 (d, *J* = 6.6 Hz, 2H), 3.77 (s, 3H), 2.26 (s, 3H). ^{13}C NMR (101 MHz, $CDCl_3$) δ 167.16, 164.35, 148.88, 137.88, 133.12, 132.79, 130.67, 128.86, 128.56, 128.17, 124.25, 117.62, 114.06, 55.57, 13.46. HRMS (ESI) *m/z* calculated for $C_{18}H_{17}N_2O_2$ $[M+H]^+$ 293.1285, found 293.1284.

(4-methoxyphenyl)(2-(pyridin-4-yl)-1H-imidazol-1-yl)methanone(B12): White solid; Yield: 12.6%; ^1H NMR (400 MHz, $CDCl_3$) δ 8.55 (s, 2H), 7.77 (d, *J* = 6.8 Hz, 2H), 7.43 (d, *J* = 4.5 Hz, 2H), 7.33 (t, *J* = 1.8 Hz, 1H), 7.25 – 7.17 (m, 1H), 6.92 (dd, *J* = 8.9, 2.2 Hz, 2H), 3.85 (s, 3H). ^{13}C NMR (101 MHz, $CDCl_3$) δ 166.79, 165.00, 149.79, 146.71, 137.88, 133.36, 129.54, 123.40, 122.58, 122.41, 114.44, 55.74. HRMS (ESI) *m/z* calculated for $C_{16}H_{14}N_3O_2$ $[M+H]^+$ 280.1081, found 280.1083.

1-benzyl 4-ethyl 1H-imidazole-1,4-dicarboxylate(B13): White solid; Yield: 8.4%; ^1H NMR (400 MHz, $CDCl_3$) δ 7.78 (s, 1H), 7.61 (s, 1H), 7.38 – 7.27 (m, 3H), 7.15 (d, *J* = 6.6 Hz, 2H), 5.51 (s, 2H), 4.26 (q, *J* = 7.1 Hz, 2H), 1.30 (t, *J* = 7.1 Hz, 3H). ^{13}C NMR (101 MHz, $CDCl_3$) δ 160.30, 138.02, 136.28, 128.88, 128.09, 127.27, 60.51, 50.08, 14.25. HRMS (ESI) *m/z* calculated for $C_{14}H_{15}N_2O_4$ $[M+H]^+$ 275.1026, found 275.0738.

ethyl1-(2-naphthoyl)-1H-imidazole-4-carboxylate(B14): White solid; Yield: 34.0%; ^1H NMR (400 MHz, $CDCl_3$) δ 8.27 (s, 1H), 8.21 (s, 1H), 8.16 (d, *J* = 1.2 Hz, 1H), 7.96 – 7.87 (m, 3H), 7.81 – 7.78 (m, 1H), 4.36 (q, *J* = 7.2 Hz, 2H), 1.36 (t, *J* = 7.1 Hz, 3H). ^{13}C NMR (101 MHz, $CDCl_3$) δ 165.75, 162.15, 138.61, 135.80, 135.18, 132.10, 129.70, 129.52, 128.10, 127.87, 127.80, 124.99, 123.73, 61.35, 14.43. HRMS (ESI) *m/z* calculated for $C_{17}H_{14}N_2O_3Na$ $[M+Na]^+$ 317.0897, found 317.0893.

ethyl1-(4-propylbenzoyl)-1H-imidazole-4-carboxylate(B15): White solid; Yield: 34.9%; ^1H NMR (400 MHz, $CDCl_3$) δ 8.18 (d, *J* = 1.4 Hz, 1H), 8.00 (d, *J* = 7.9 Hz,

1H), 7.72 (d, *J* = 8.0 Hz, 2H), 7.37 (d, *J* = 8.0 Hz, 2H), 4.38 (q, *J* = 7.2 Hz, 2H), 2.68 (t, *J* = 7.7 Hz, 2H), 1.68 – 1.64 (m, 2H), 1.37 (t, *J* = 7.1 Hz, 3H), 0.95 (t, *J* = 6.7 Hz, 3 H). ^{13}C NMR (101 MHz, $CDCl_3$) δ 165.59, 162.22, 150.48, 138.51, 135.03, 130.76, 13 0.35, 130.16, 129.47, 129.11, 128.59, 128.07, 123.72, 61.36, 60.78, 38.17, 24.27, 14.4 2, 13.89. HRMS (ESI) *m/z* calculated for $C_{16}H_{18}N_2O_3Na$ $[M+Na]^+$ 309.1210, found 30 9.1205.

phenyl1-(4-methoxybenzoyl)-4-methyl-2-propyl-1H-benzo[d]imidazole-6-carboxylate(B16): White solid; Yield: 65%; ^1H NMR (400 MHz, $CDCl_3$) δ 8.14 (d, *J* = 9.0 Hz, 2H), 8.00 (s, 1H), 7.81 (d, *J* = 8.9 Hz, 2H), 7.65 (s, 1H), 7.48 – 7.40 (m, 2H), 7.29 (d, *J* = 9.2 Hz, 1H), 7.22 – 7.15 (m, 2H), 7.05 – 7.00 (m, 4H), 3.93 (s, 6H), 3.17 – 3.07 (m, 2H), 2.78 (s, 3H), 1.90 (h, *J* = 7.4 Hz, 2H), 1.04 (t, *J* = 7.4 Hz, 3H). ^{13}C NM R (101 MHz, $CDCl_3$) δ 167.65, 165.38, 164.98, 164.60, 162.31, 159.08, 151.02, 145.5 6, 133.80, 133.06, 132.84, 129.58, 129.42, 125.78, 125.73, 124.63, 124.25, 121.69, 12 1.28, 114.56, 114.15, 113.07, 55.70, 55.61, 31.62, 21.91, 16.80, 13.97. HRMS (ESI) *m /z* calculated for $C_{26}H_{25}N_2O_4$ $[M+H]^+$ 429.1809, found 429.1806.

2,4-diphenyl-1-(3-(pyrrolidin-1-yl)propyl)-1H-imidazole(C1): White solid; Yi eld: 47.5%; ^1H NMR (400 MHz, $CDCl_3$) δ 7.86 – 7.80 (m, 2H), 7.62 (dd, *J* = 7.7, 1.8 Hz, 2H), 7.47 – 7.40 (m, 3H), 7.38 – 7.32 (m, 3H), 7.23 (d, *J* = 7.3 Hz, 1H), 4.06 (t, *J* = 7.2 Hz, 2H), 2.44 (dt, *J* = 20.3, 6.9 Hz, 6H), 1.95 (p, *J* = 7.1 Hz, 2H), 1.78 – 1.71 (m, 4H). ^{13}C NMR (101 MHz, $CDCl_3$) δ 148.21, 141.17, 134.25, 130.87, 129.17, 128.99, 128.72, 128.65, 126.80, 54.01, 52.67, 44.90, 29.92, 23.47. HRMS(ESI) *m/z* calculated for $C_{22}H_{26}N_3$ $[M+H]^+$ 332.2121,found 332.2118.

1-(3-(2,4-diphenyl-1H-imidazol-1-yl)propyl)piperidine(C2): White solid; Yiel d: 42.6%; ^1H NMR (400 MHz, $CDCl_3$) δ 7.87 – 7.81 (m, 2H), 7.66 – 7.62 (m, 2H), 7.4 6 – 7.34 (m, 5H), 7.31 (s, 1H), 7.22 (t, *J* = 7.4 Hz, 1H), 4.02 (t, *J* = 7.2 Hz, 2H), 2.39 – 2.13 (m, 6H), 1.88 (p, *J* = 7.1 Hz, 2H), 1.51 (p, *J* = 5.6 Hz, 4H), 1.42 – 1.34 (m, 2H). ^{13}C NMR (101 MHz, $CDCl_3$) δ 148.20, 141.08, 134.38, 130.98, 129.19, 128.89, 128.6 7, 128.64, 126.72, 124.91, 116.54, 55.41, 54.52, 44.92, 28.30, 26.02, 24.47. HRMS (E SI) *m/z* calculated for $C_{23}H_{28}N_3$ $[M+H]^+$ 346.2278, found 346.2271.

4-(3-(2,4-diphenyl-1H-imidazol-1-yl)propyl)morpholine(C3): White solid; Yield: 37.5%; ^{1}H NMR (400 MHz, $CDCl_3$) δ 7.83 (d, *J* = 7.7 Hz, 2H), 7.63 (d, *J* = 7.3 Hz, 2H), 7.43 (q, *J* = 8.0, 7.6 Hz, 3H), 7.36 (t, *J* = 7.6 Hz, 2H), 7.29 (s, 1H), 7.22 (t, *J* = 7.4 Hz, 1H), 4.07 (t, *J* = 7.3 Hz, 2H), 3.59 (t, *J* = 4.6 Hz, 4H), 2.27 (s, 4H), 2.22 (t, *J* = 6.8 Hz, 2H), 1.85 (p, *J* = 7.1 Hz, 2H). ^{13}C NMR (101 MHz, $CDCl_3$) δ 148.25, 141.19, 134.28, 130.97, 129.18, 128.94, 128.68, 126.80, 124.89, 116.39, 66.97, 54.88, 53.52, 44.61, 27.82. HRMS (ESI) *m/z* calculated for $C_{22}H_{26}N_3O$ $[M+H]^+$ 348.2070, found 348.2067.

4-(3-(2,4-diphenyl-1H-imidazol-1-yl)propoxy)phenol(C4): White solid; Yield: 16.4%; ^{1}H NMR (400 MHz, $CDCl_3$) δ 7.79 (d, *J* = 7.7 Hz, 2H), 7.33 (d, *J* = 6.1 Hz, 5H), 7.30 (s, 1H), 7.22 (t, *J* = 7.4 Hz, 1H), 6.61 (d, *J* = 8.4 Hz, 2H), 6.51 (d, *J* = 8.4 Hz, 2H), 4.20 (t, *J* = 6.9 Hz, 2H), 3.76 (t, *J* = 5.5 Hz, 2H), 2.13 (t, *J* = 6.3 Hz, 2H), 2.06 (s, 1H). ^{13}C NMR (101 MHz, $CDCl_3$) δ 151.56, 151.12, 148.71, 141.16, 133.61, 129.86, 129.29, 129.26, 128.78, 128.71, 127.10, 125.15, 116.49, 116.40, 115.24, 64.23, 43.69, 30.74, 29.84, 22.84, 14.29. HRMS (ESI) *m/z* calculated for $C_{24}H_{23}N_2O_2$ $[M+H]^+$ 371.1754, found 371.1749.

2,4-diphenyl-1-(2-(2-(pyrrolidin-1-yl)ethoxy)ethyl)-1H-imidazole(C5): White solid; Yield:41.3%; ^{1}H NMR (400 MHz, $CDCl_3$) δ 7.83 (d, *J* = 7.6 Hz, 2H), 7.64 (d, *J* = 7.0 Hz, 2H), 7.44 (q, *J* = 7.9, 7.0 Hz, 4H), 7.36 (t, *J* = 7.6 Hz, 2H), 7.22 (t, *J* = 7.2 Hz, 1H), 4.17 (t, *J* = 4.9 Hz, 2H), 3.72 (t, *J* = 5.0 Hz, 2H), 3.58 (t, *J* = 5.4 Hz, 2H), 2.69 (t, *J* = 5.4 Hz, 2H), 2.55 (s, 4H), 1.75 (s, 4H). ^{13}C NMR (101 MHz, $CDCl_3$) δ 148.41, 141.17, 134.27, 130.72, 129.45, 128.98, 128.69, 128.61, 126.76, 124.96, 116.90, 70.47, 70.35, 55.50, 54.78, 46.75, 23.46. HRMS (ESI) *m/z* calculated for $C_{23}H_{28}N_3O$ $[M+H]^+$ 362.2227, found 362.2220.

1-(2-(2-(2,4-diphenyl-1H-imidazol-1-yl)ethoxy)ethyl)piperidine(C6): White solid; Yield: 41.9%; ^{1}H NMR (400 MHz, $CDCl_3$) δ 7.81 (d, *J* = 7.3 Hz, 2H), 7.64 – 7.60 (m, 2H), 7.40 (d, *J* = 7.1 Hz, 4H), 7.33 (t, *J* = 7.7 Hz, 2H), 7.19 (t, *J* = 7.4 Hz, 1H), 4.09 (t, *J* = 5.2 Hz, 2H), 3.65 (t, *J* = 5.2 Hz, 2H), 3.52 (t, *J* = 5.6 Hz, 2H), 2.50 (t, *J* = 5.6 Hz, 2H), 2.37 (s, 4H), 1.52 (p, *J* = 5.6 Hz, 4H), 1.39 – 1.31 (m, 2H). ^{13}C NMR (101 MHz, $CDCl_3$) δ 148.40, 141.08, 134.33, 130.72, 129.43, 128.95, 128.67, 128.59, 12

6.73, 124.94, 116.87, 70.32, 69.18, 58.38, 54.97, 46.71, 25.67, 24.02.HRMS (ESI) *m/z* calculated for $C_{24}H_{30}N_3O$ $[M+H]^+$ 376.2383, found 376.2379.

4-(2-(2-(2,4-diphenyl-1H-imidazol-1-yl)ethoxy)ethyl)morpholine(C7): White solid; Yield: 26.5%; ^{1}H NMR (400 MHz, $CDCl_3$) δ 7.81 (d, *J* = 7.4 Hz, 2H), 7.66 – 7.60 (m, 2H), 7.41 (dd, *J* = 15.3, 7.9 Hz, 4H), 7.34 (t, *J* = 7.7 Hz, 2H), 7.20 (t, *J* = 7.3 Hz, 1H), 4.10 (t, *J* = 5.1 Hz, 2H), 3.65 (t, *J* = 5.2 Hz, 2H), 3.63 – 3.55 (m, 4H), 3.48 (t, *J* = 5.4 Hz, 2H), 2.48 (t, *J* = 5.4 Hz, 2H), 2.38 (s, 4H).^{13}C NMR (101 MHz, $CDCl_3$) δ 148.41, 141.11, 134.27, 130.73, 129.44, 128.97, 128.68, 128.63, 126.79, 124.90, 116.79, 70.39, 69.30, 66.92, 58.25, 54.15, 46.75. HRMS (ESI) *m/z* calculated for $C_{23}H_{28}N_3O_2$ $[M+H]^+$ 378.2176, found 378.2172.

^{1}H NMR of **A1**

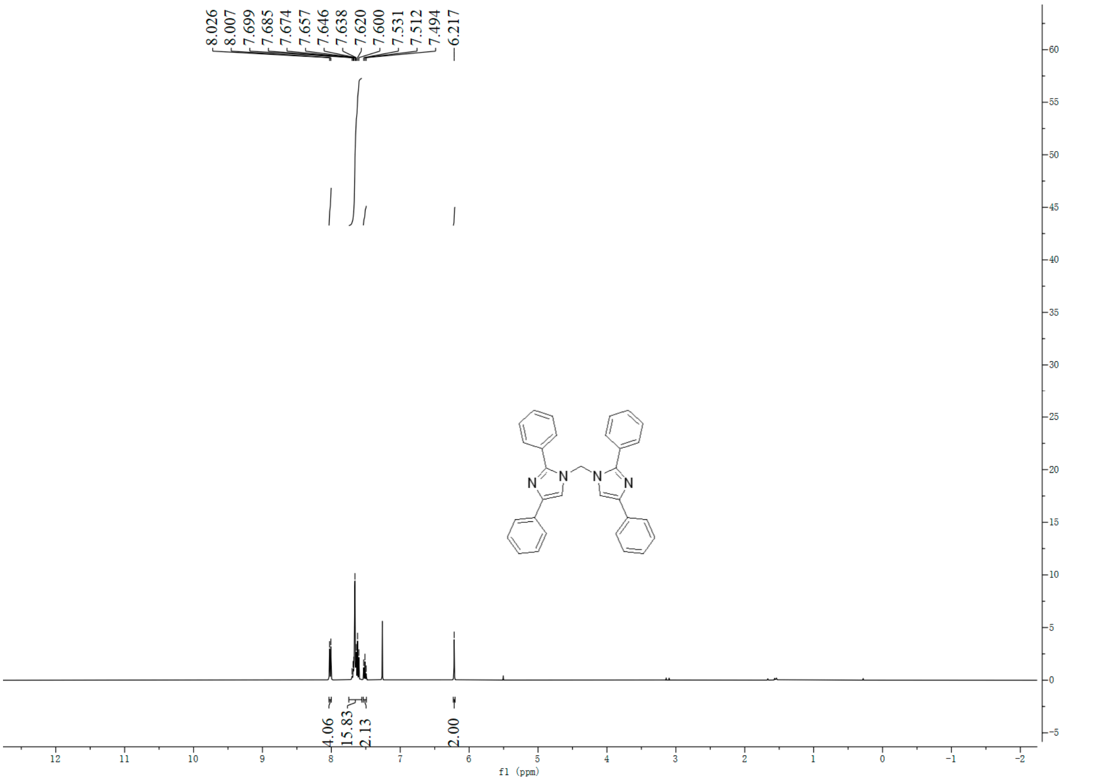

^{13}C NMR of **A1**

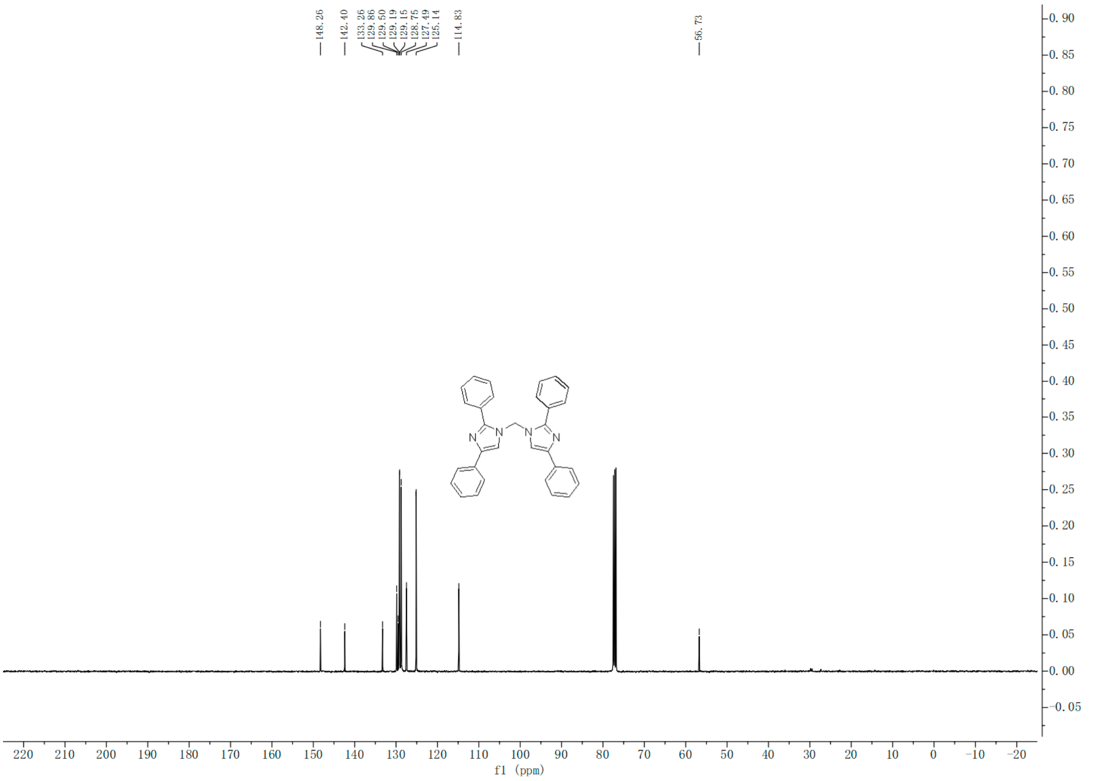

^{1}H NMR of **A2**

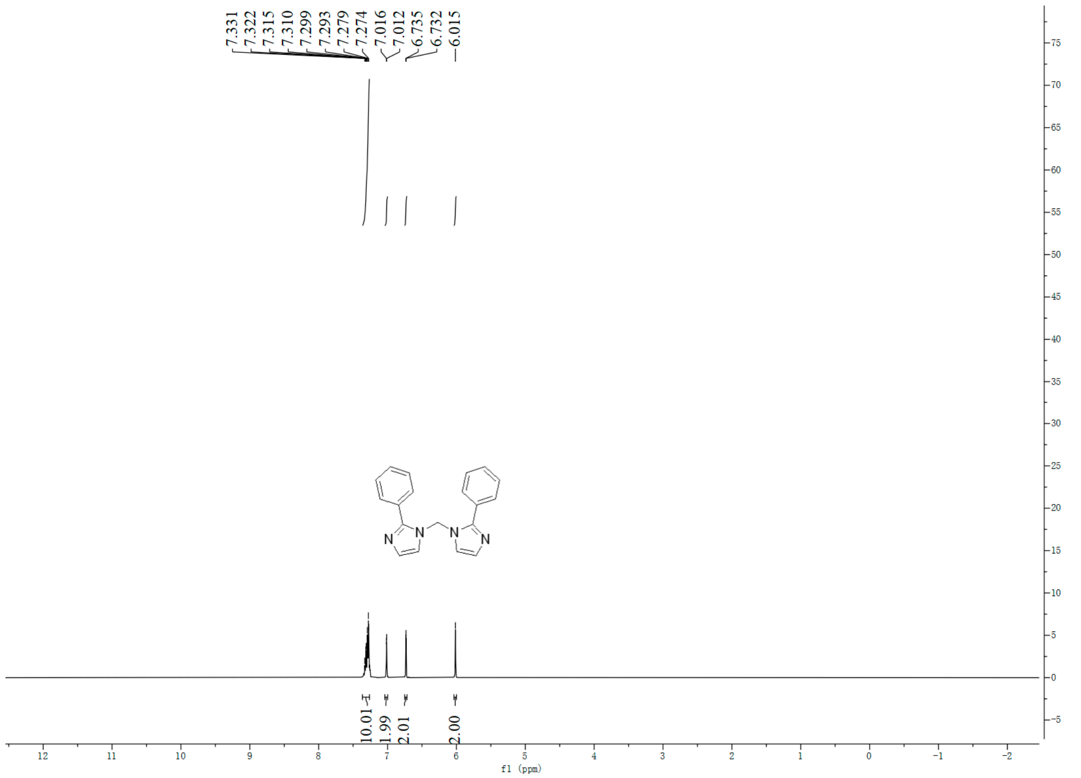

^{13}C NMR of **A2**

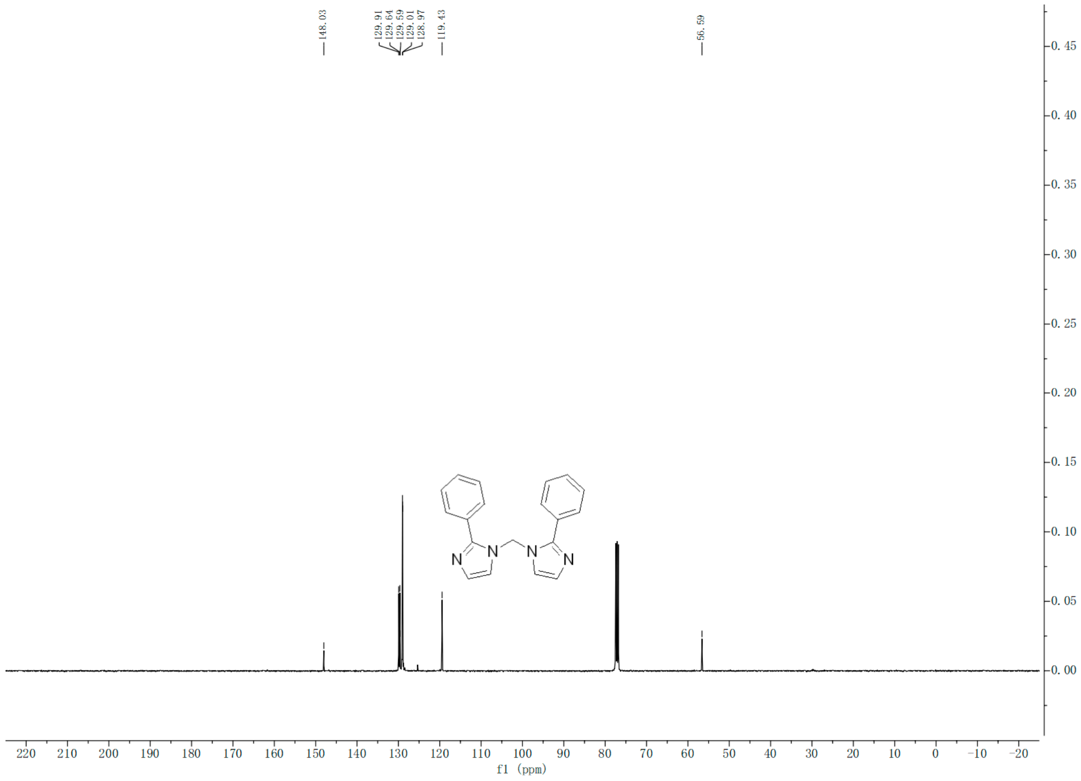

^{1}H NMR of **A3**

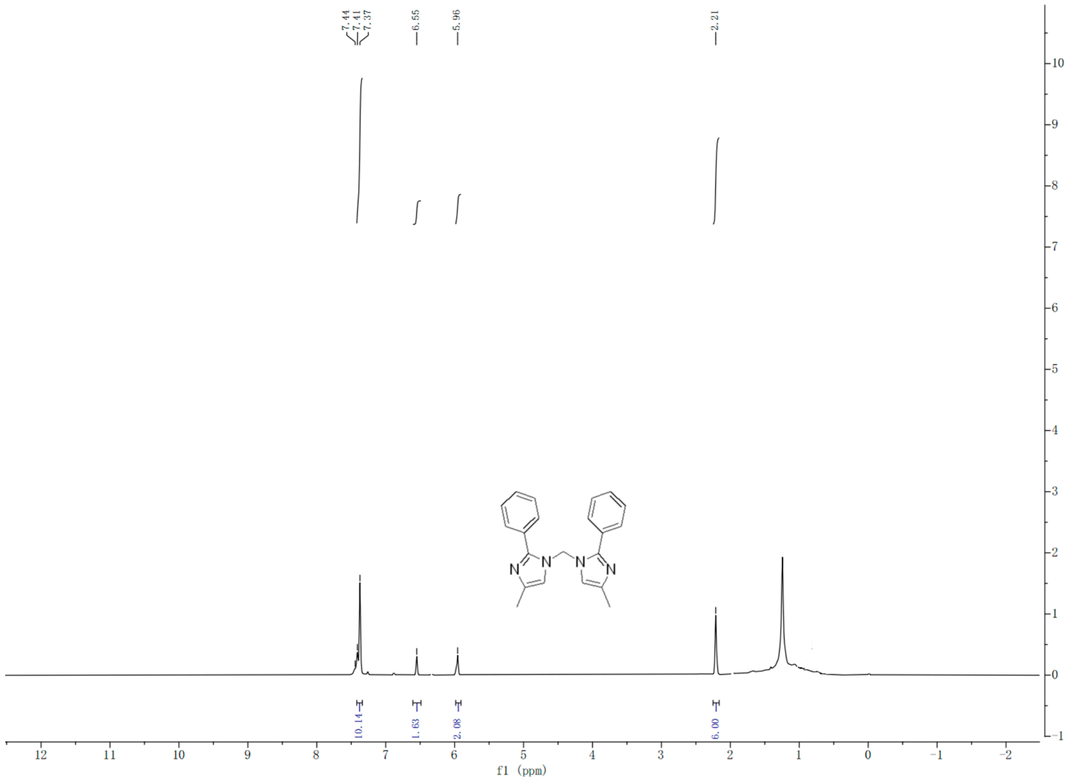

^{13}C NMR of **A3**

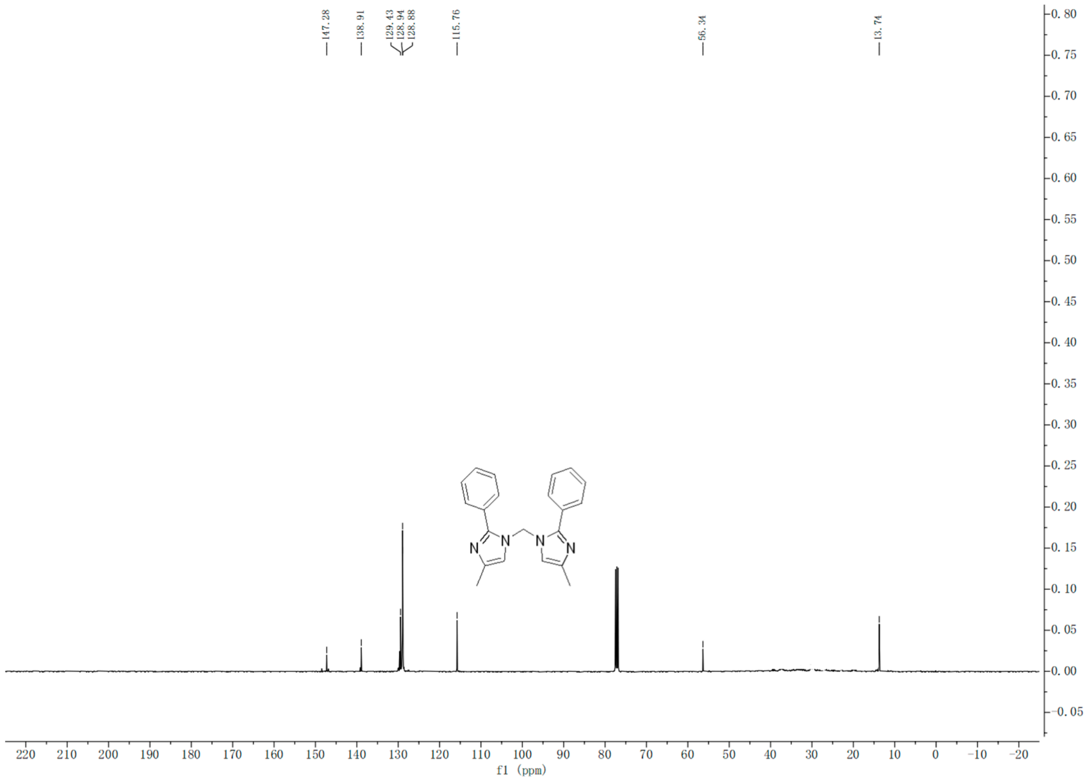

^{1}H NMR of **A4**

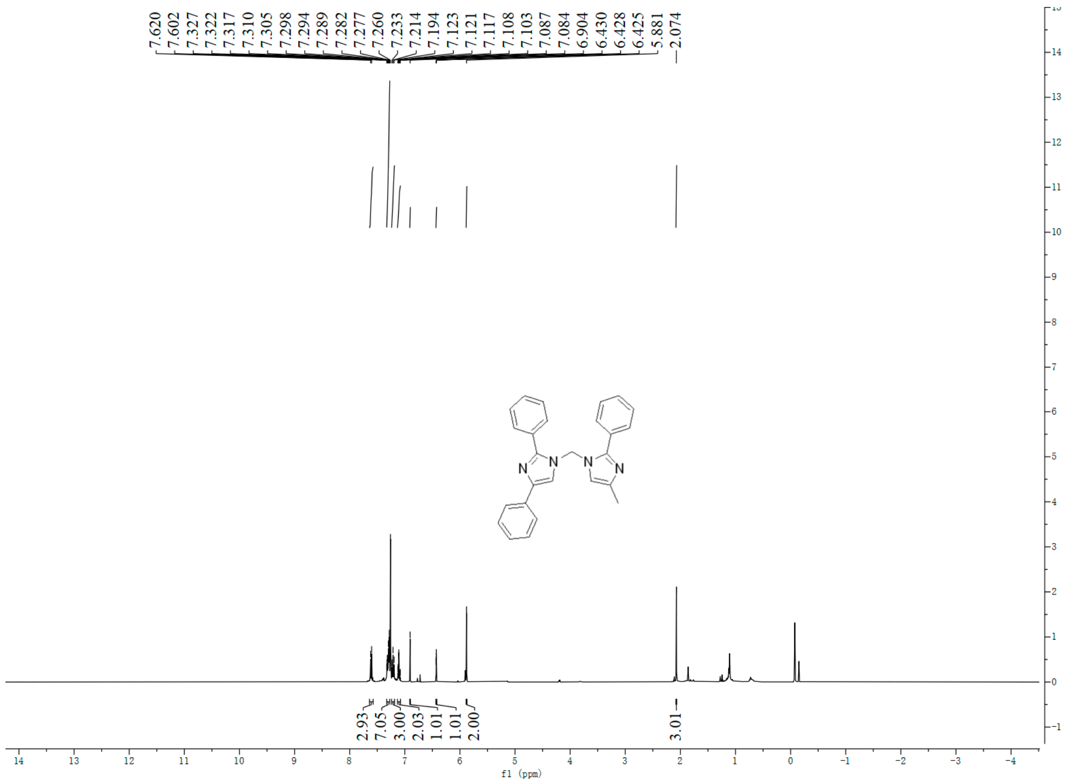

^{13}C NMR of **A4**

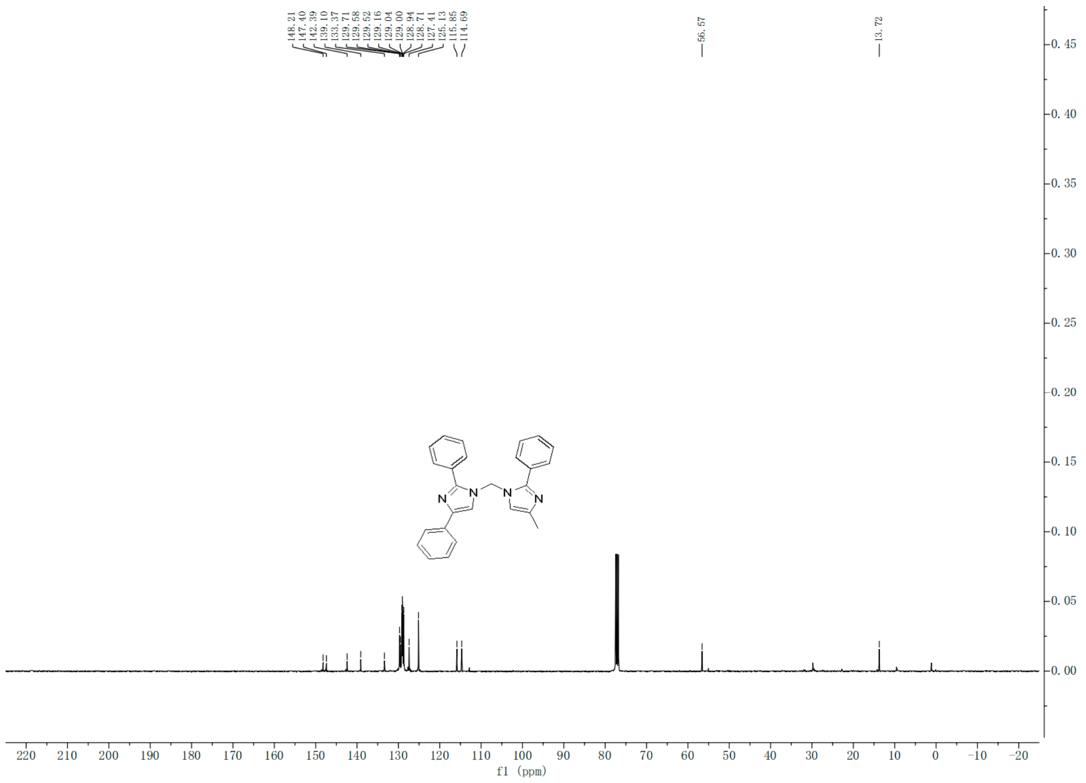

^{1}H NMR of **A5**

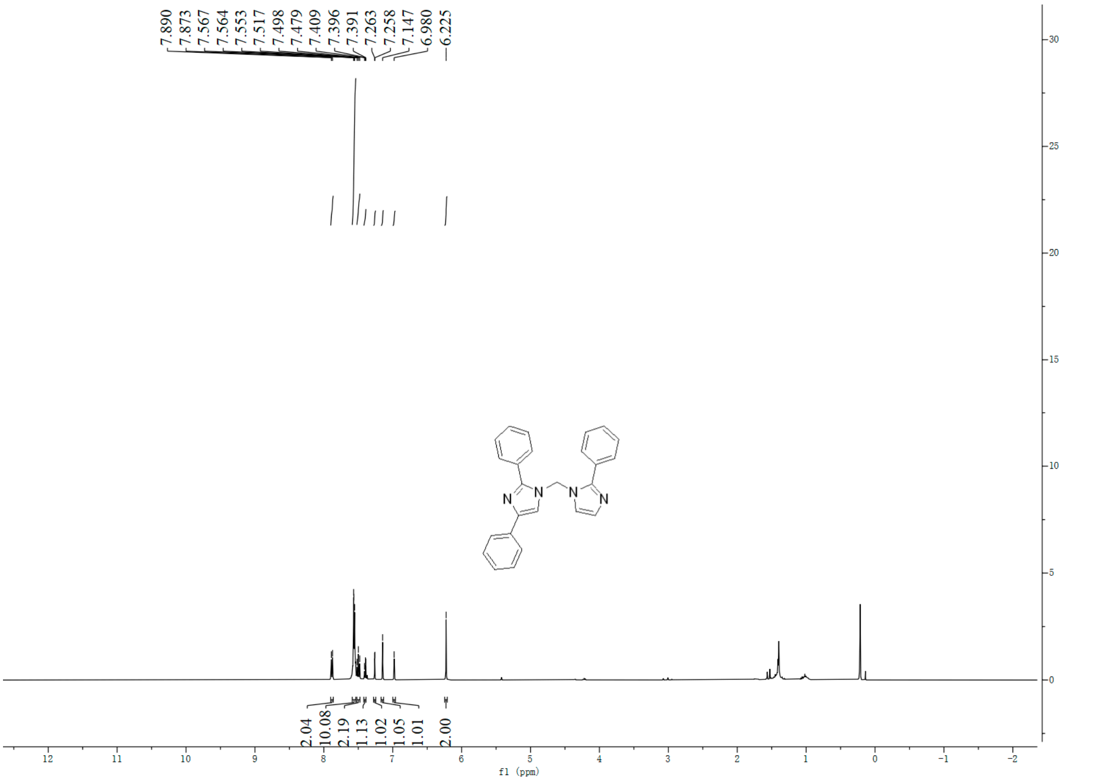

^{13}C NMR of **A5**

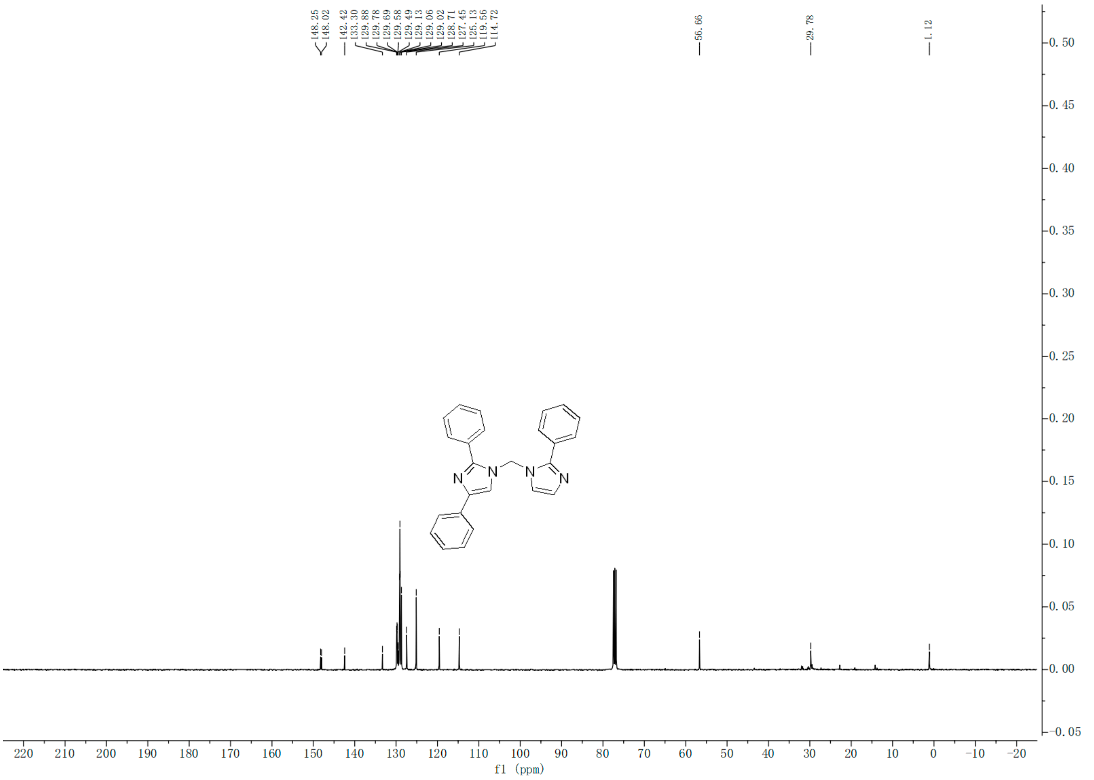

^{1}H NMR of **A6**

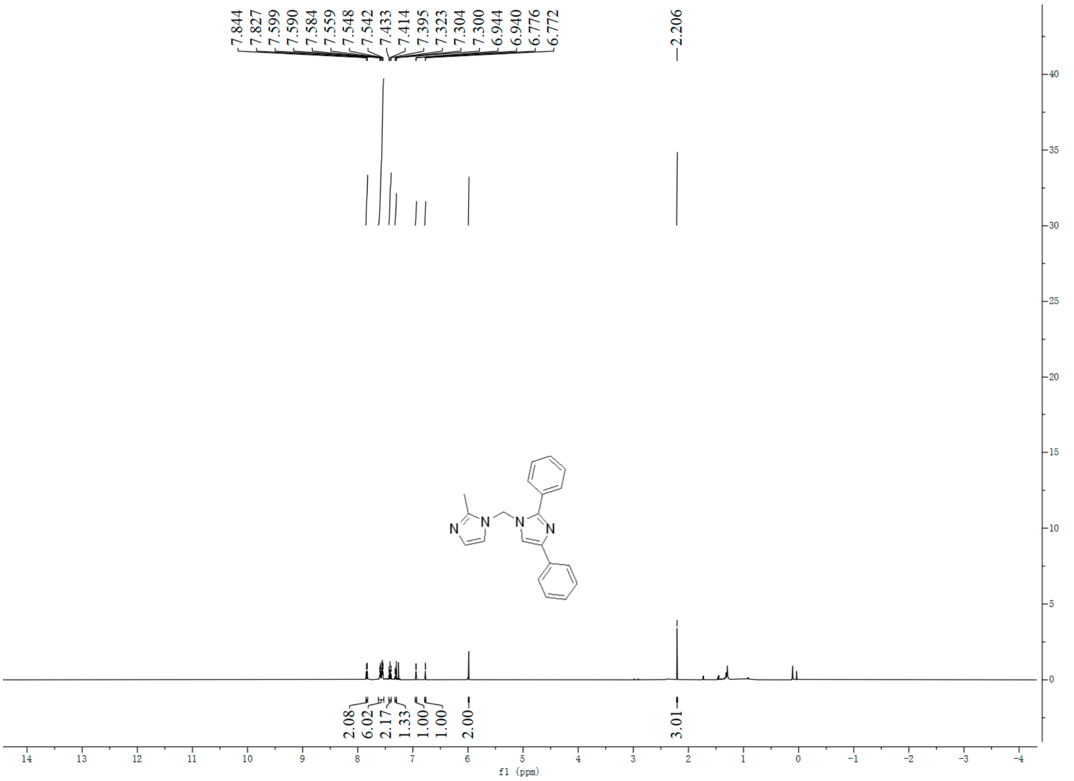

^{13}C NMR of **A6**

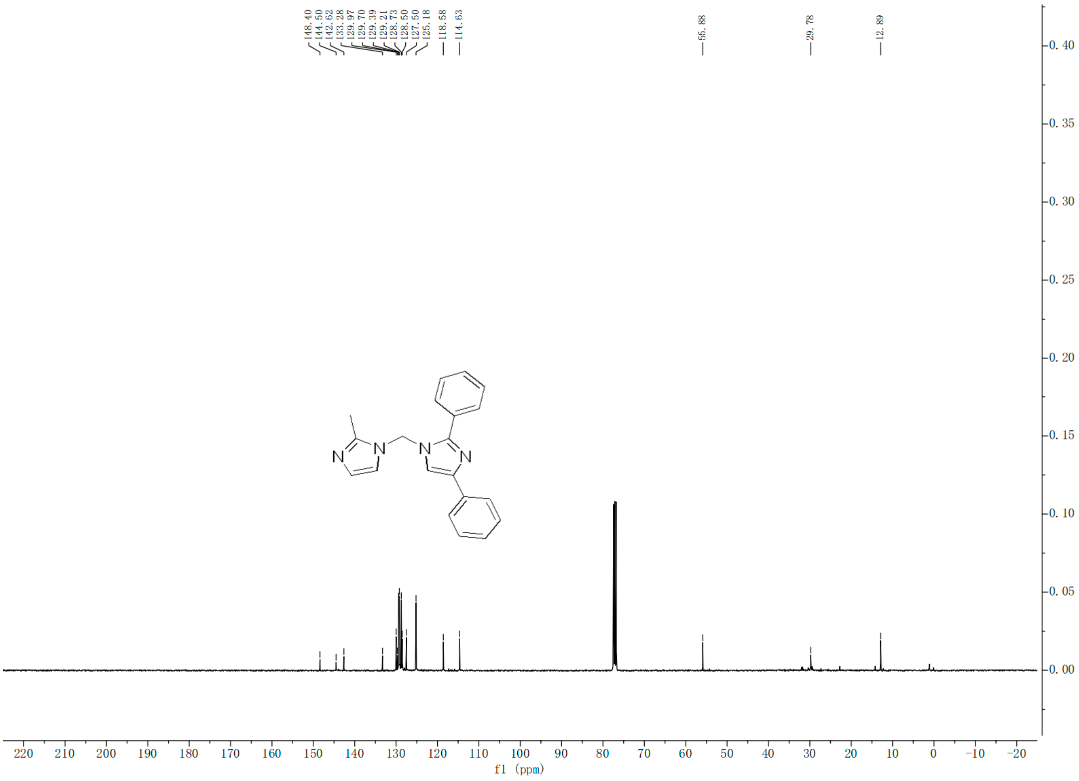

^{1}H NMR of **A7**

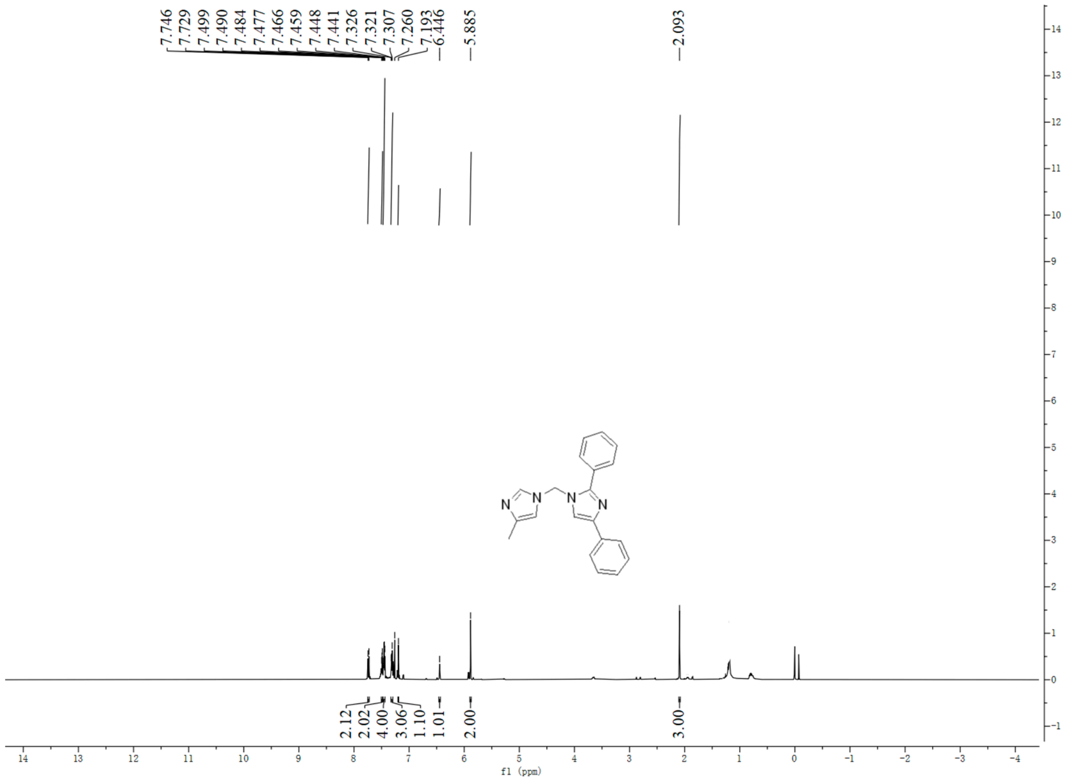

^{13}C NMR of **A7**

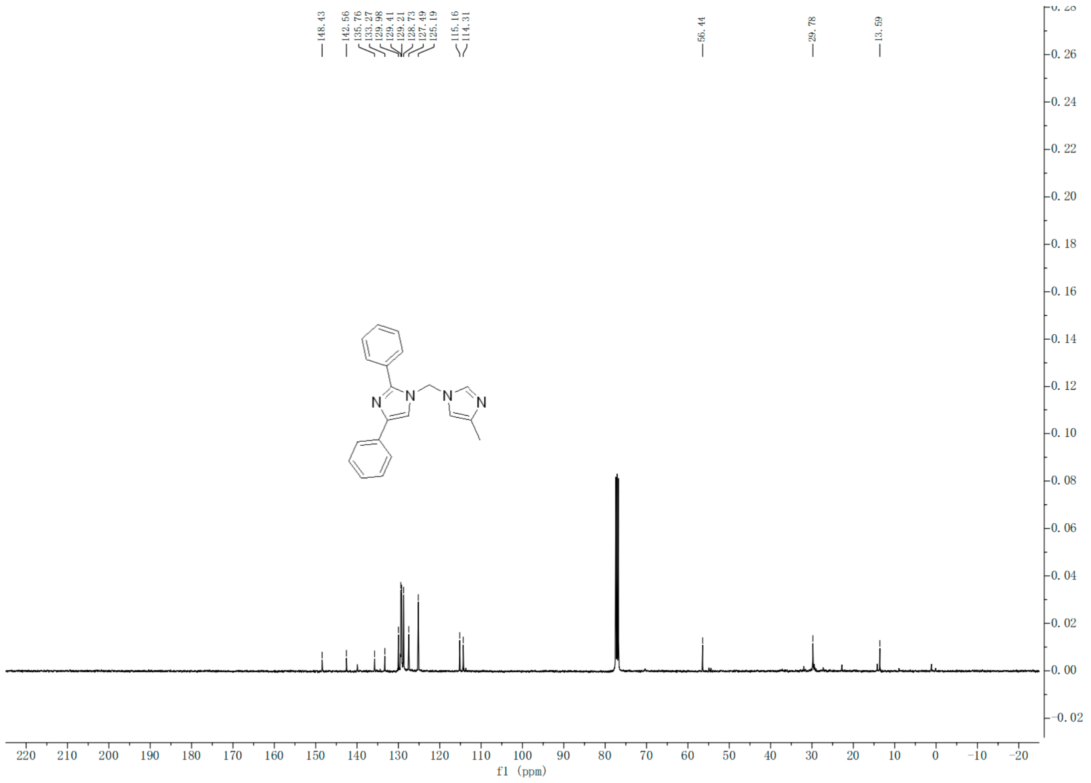

^{1}H NMR of **A8**

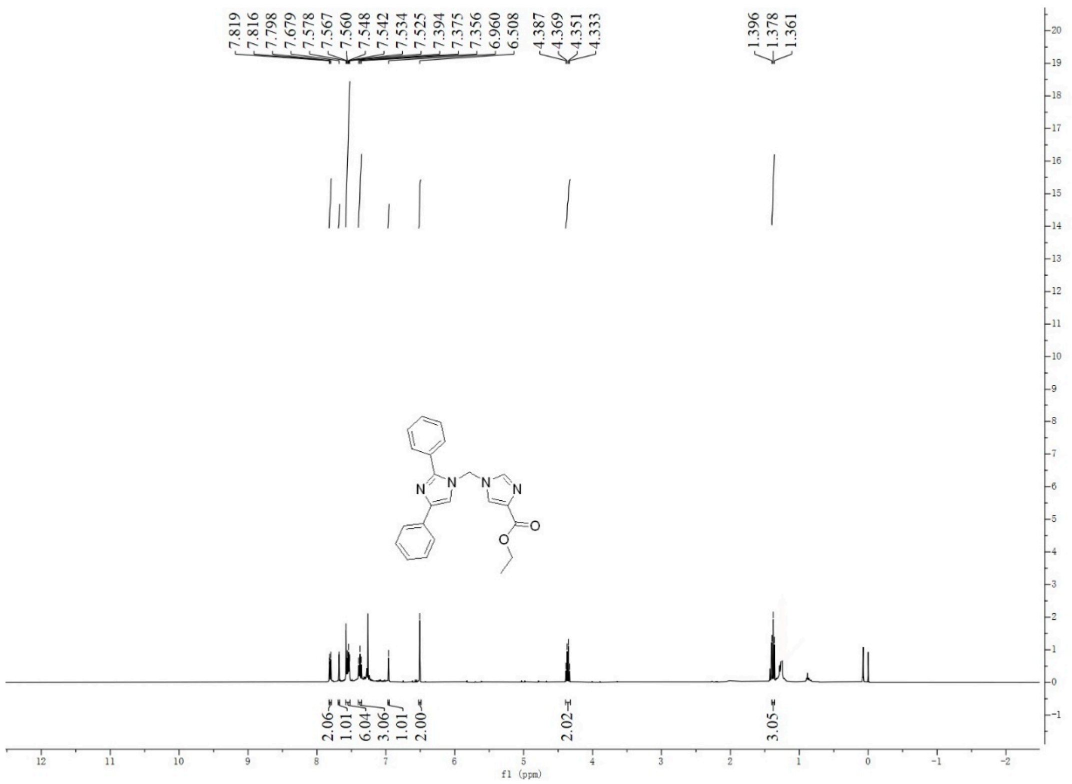

^{13}C NMR of **A8**

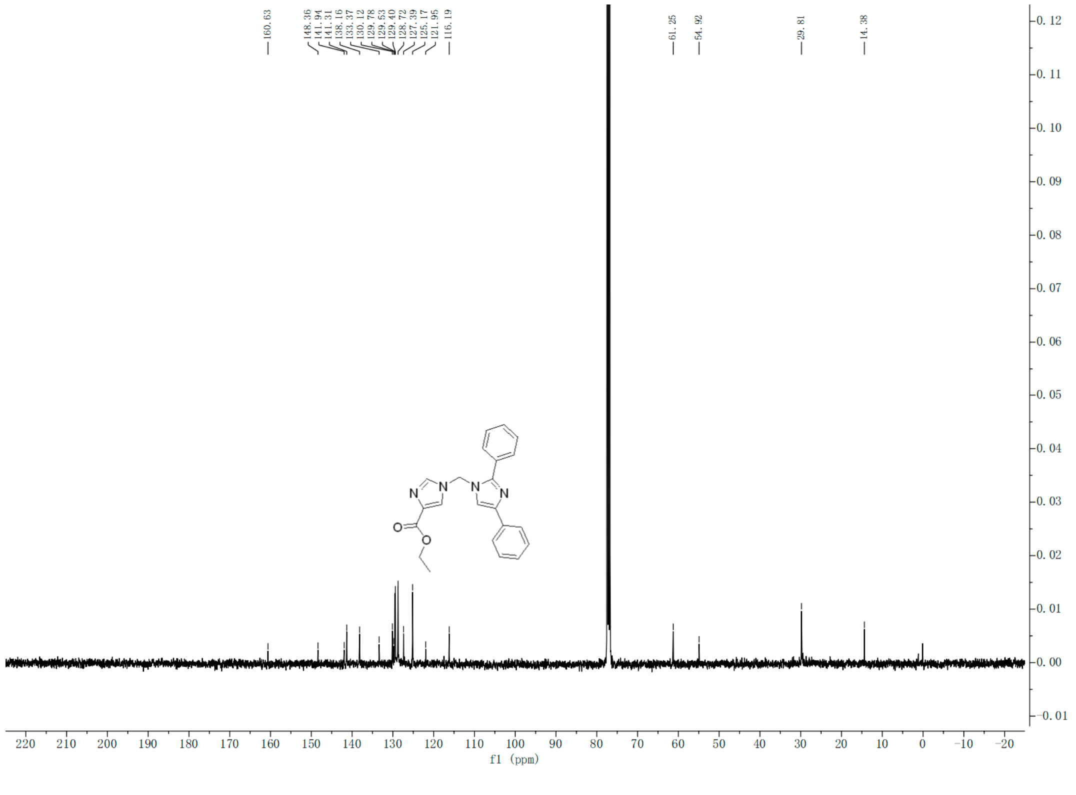

^{1}H NMR of **A9**

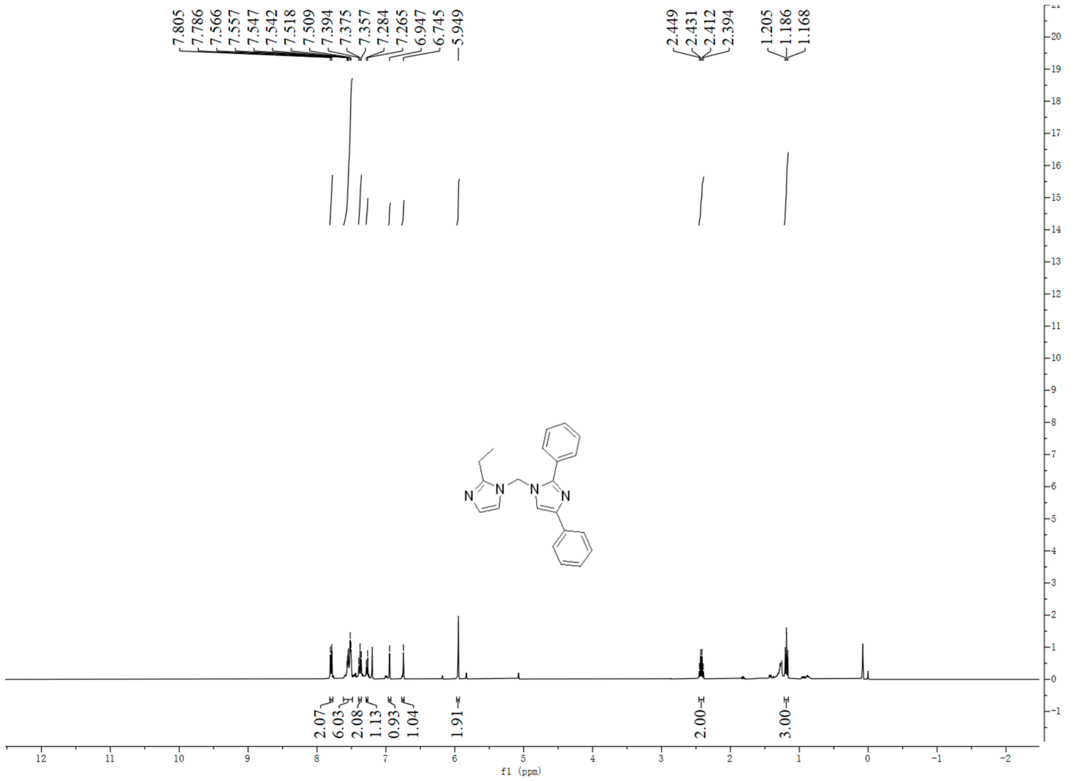

^{13}C NMR of **A9**

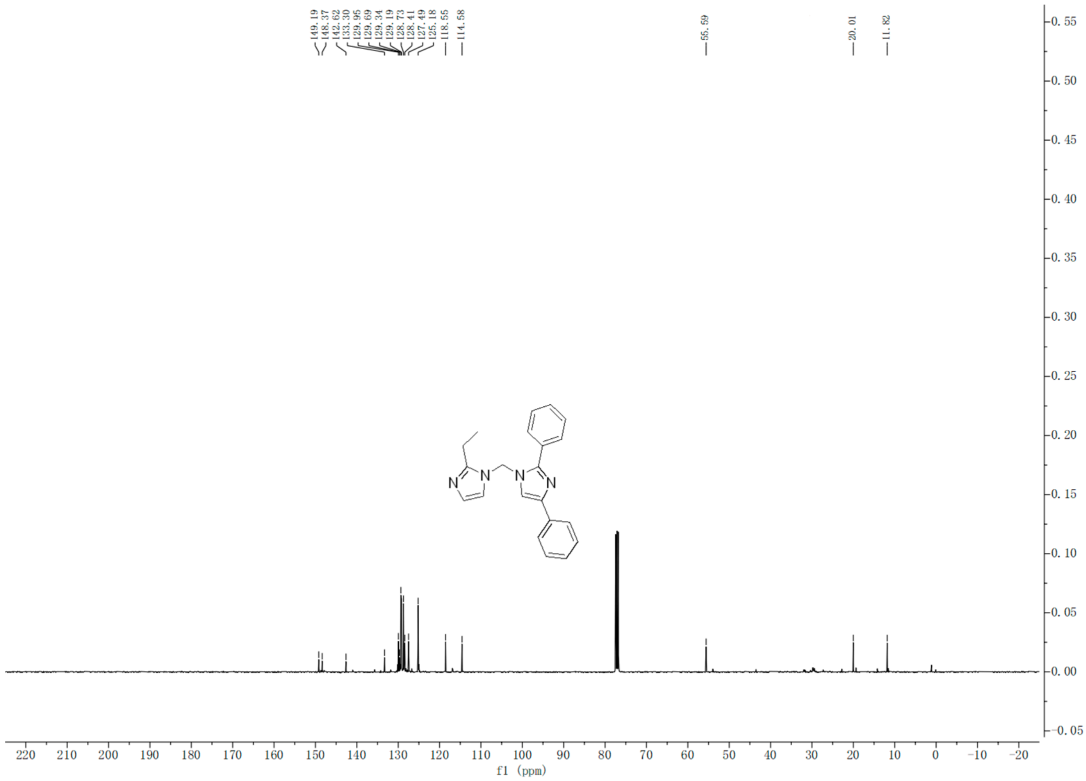

^{1}H NMR of **A10**

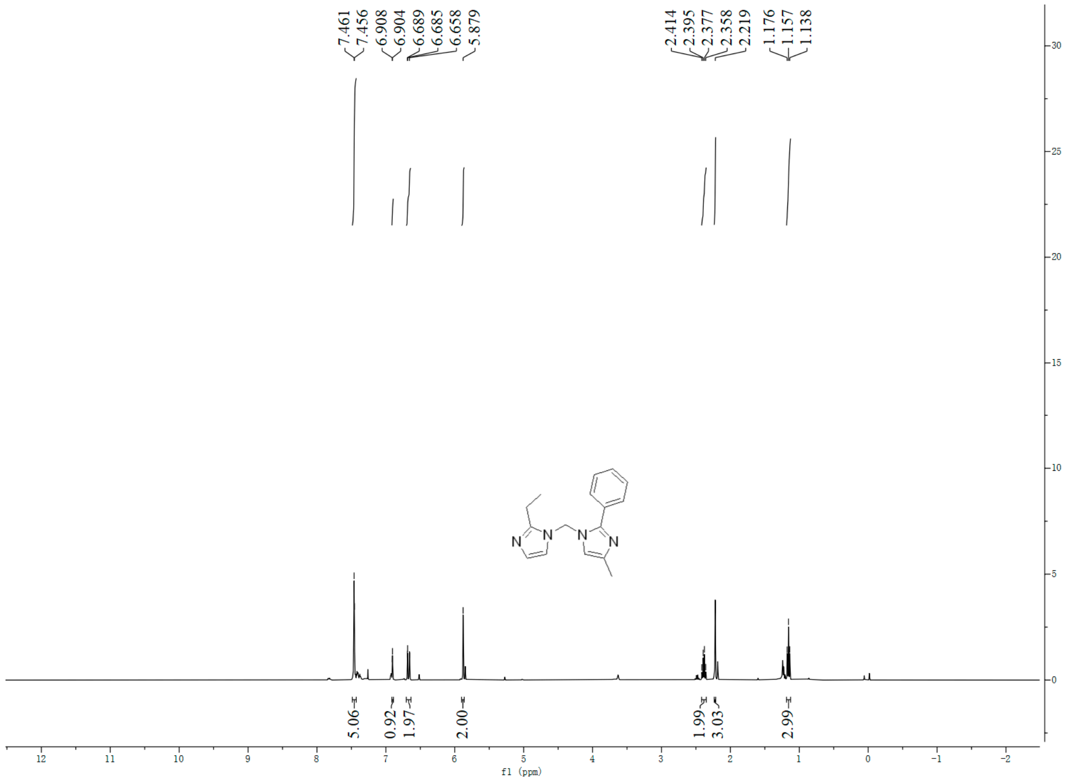

^{13}C NMR of **A10**

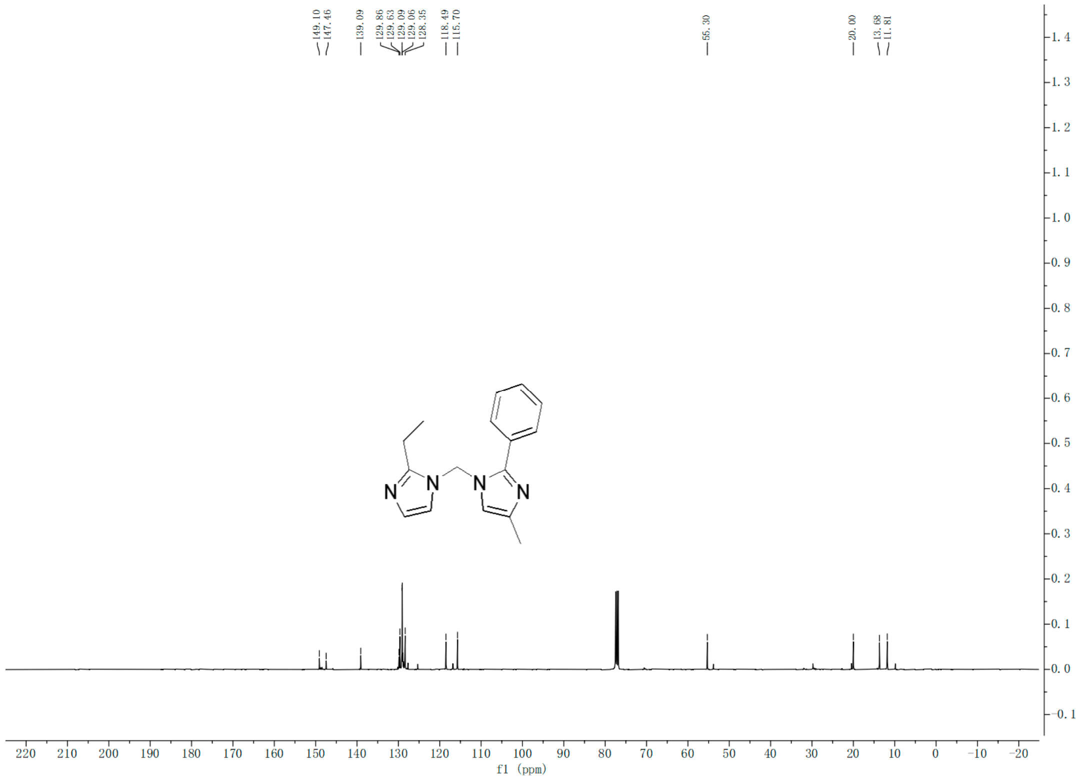

^{1}H NMR of **A11**

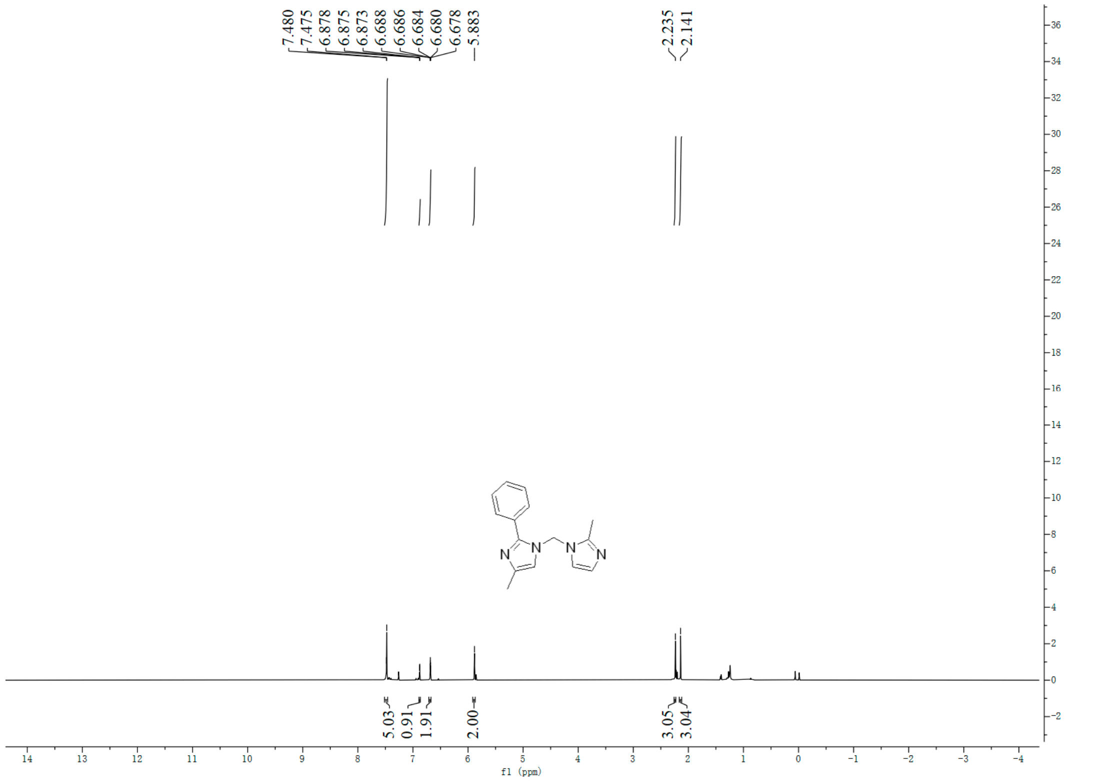

^{13}C NMR of **A11**

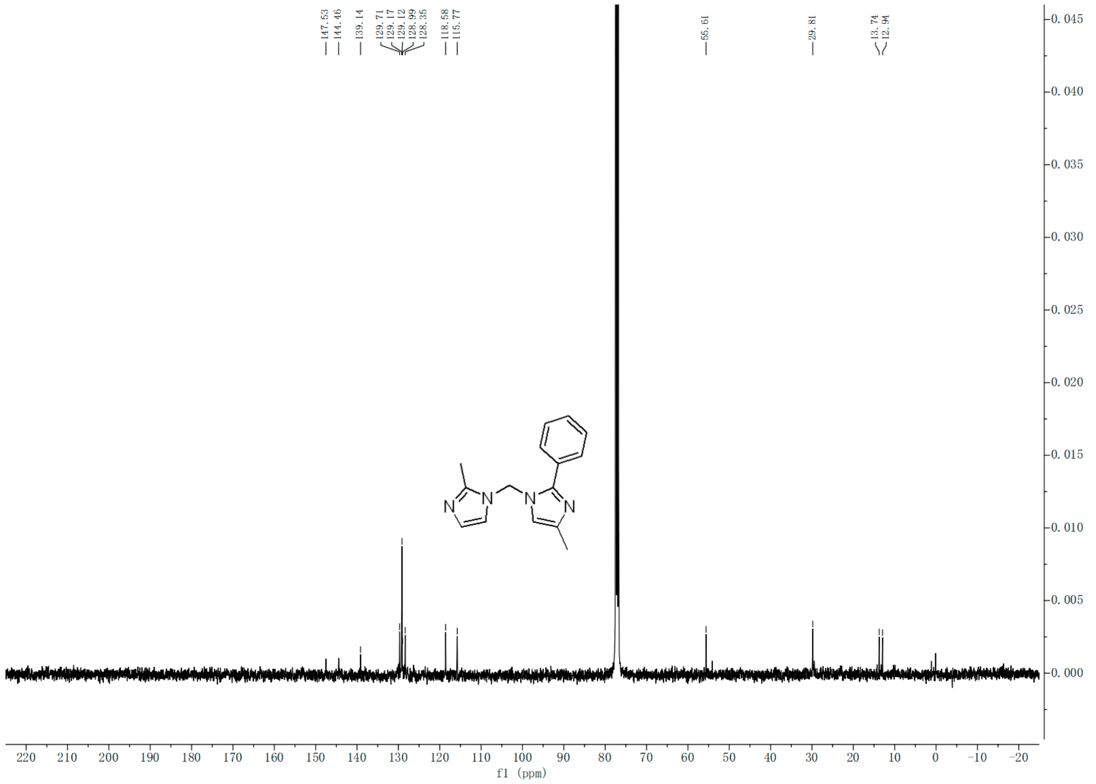

^{1}H NMR of **A12**

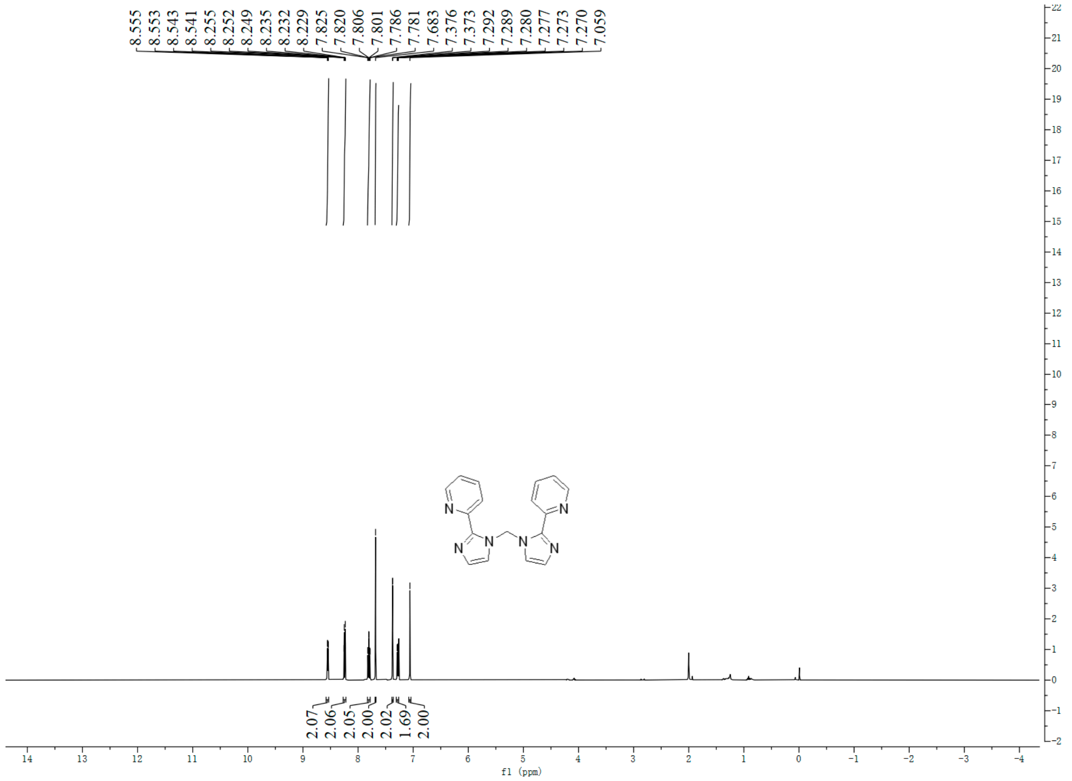

^{13}C NMR of **A12**

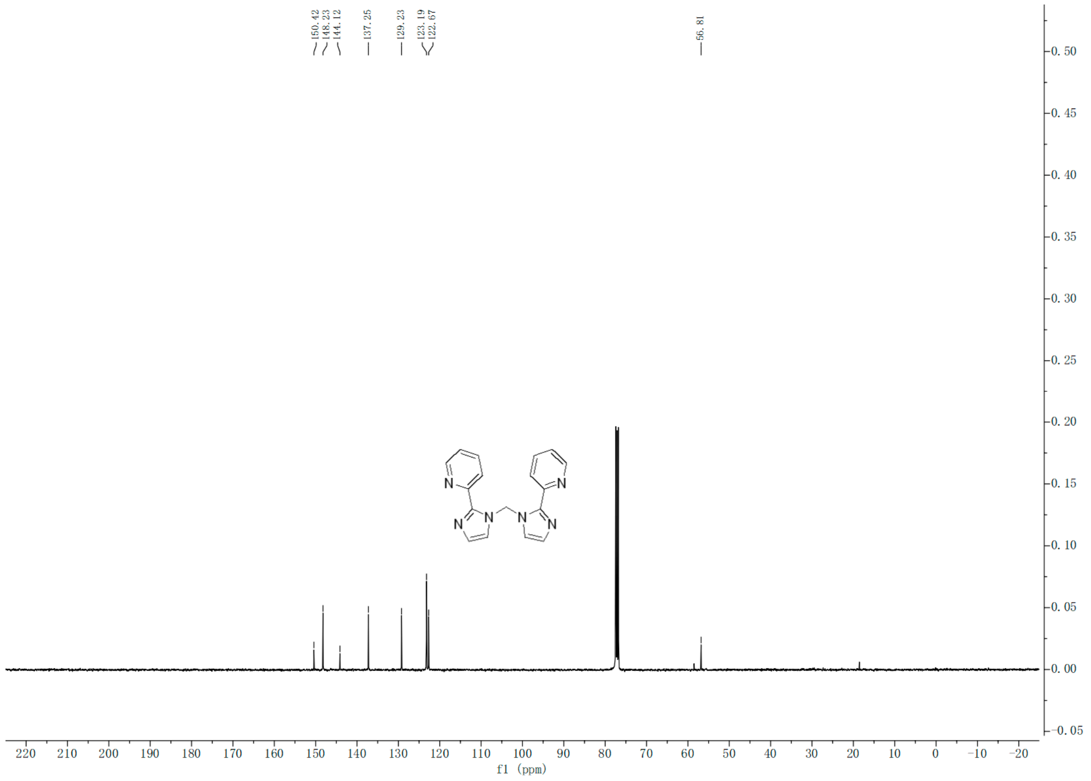

^{1}H NMR of **A13**

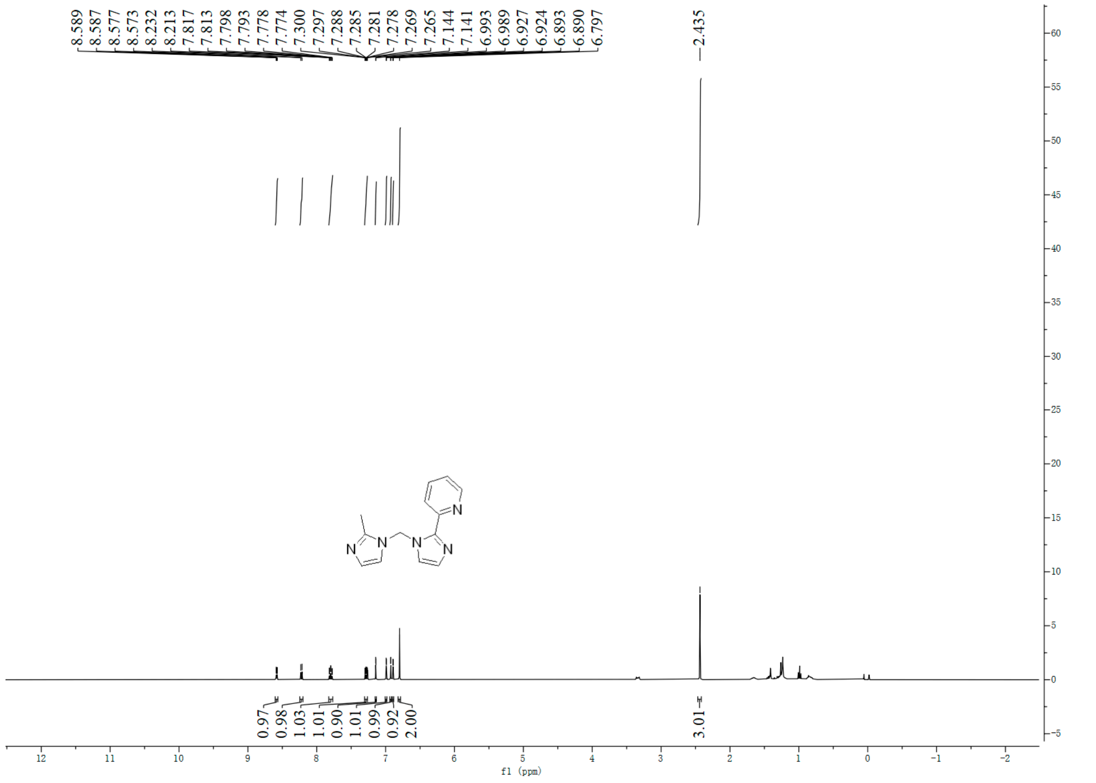

^{13}C NMR of **A13**

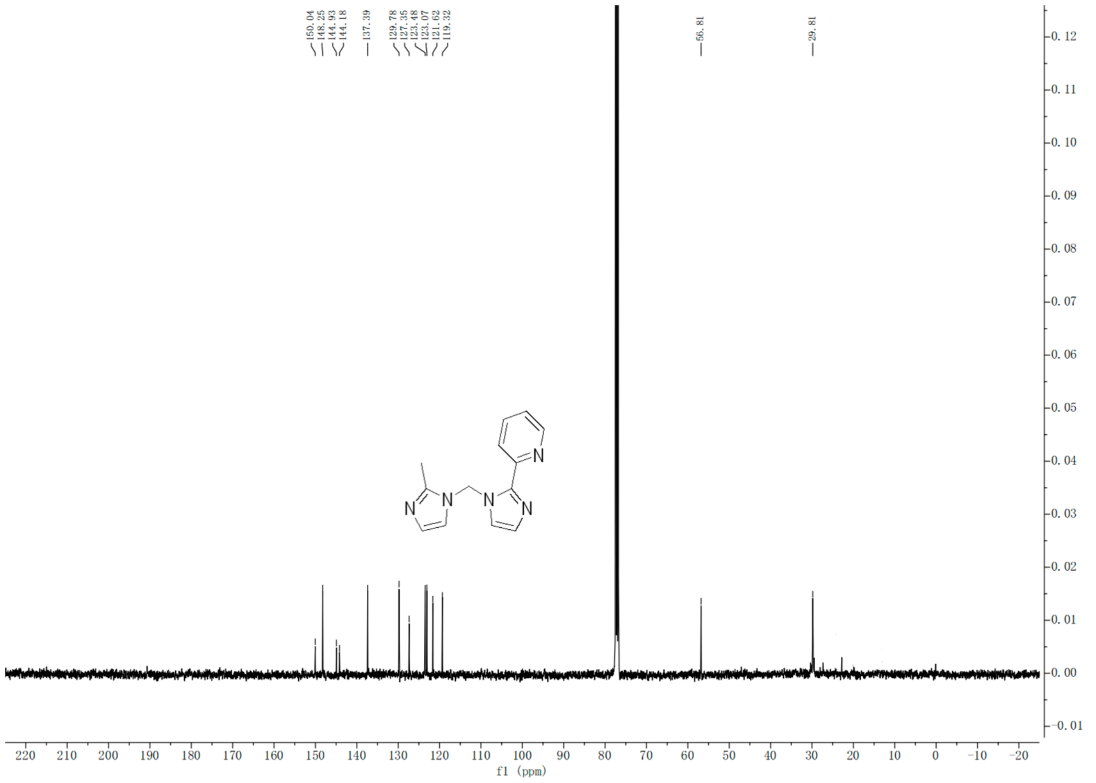

^{1}H NMR of **A14**

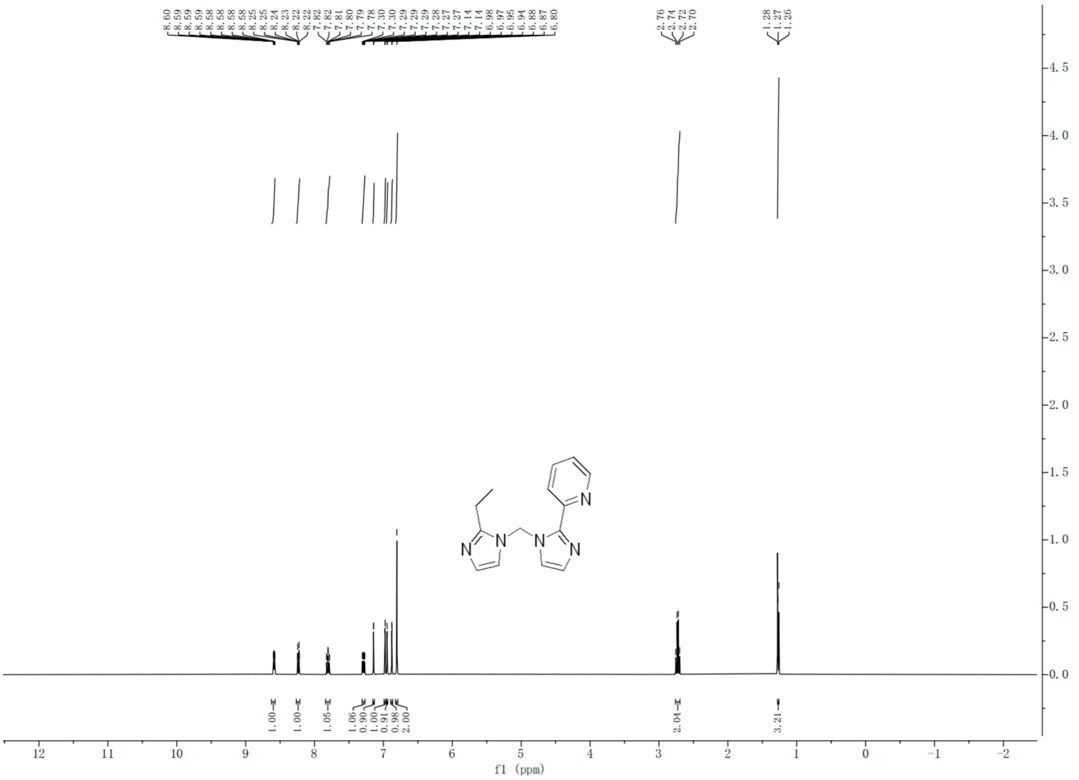

^{13}C NMR of **A14**

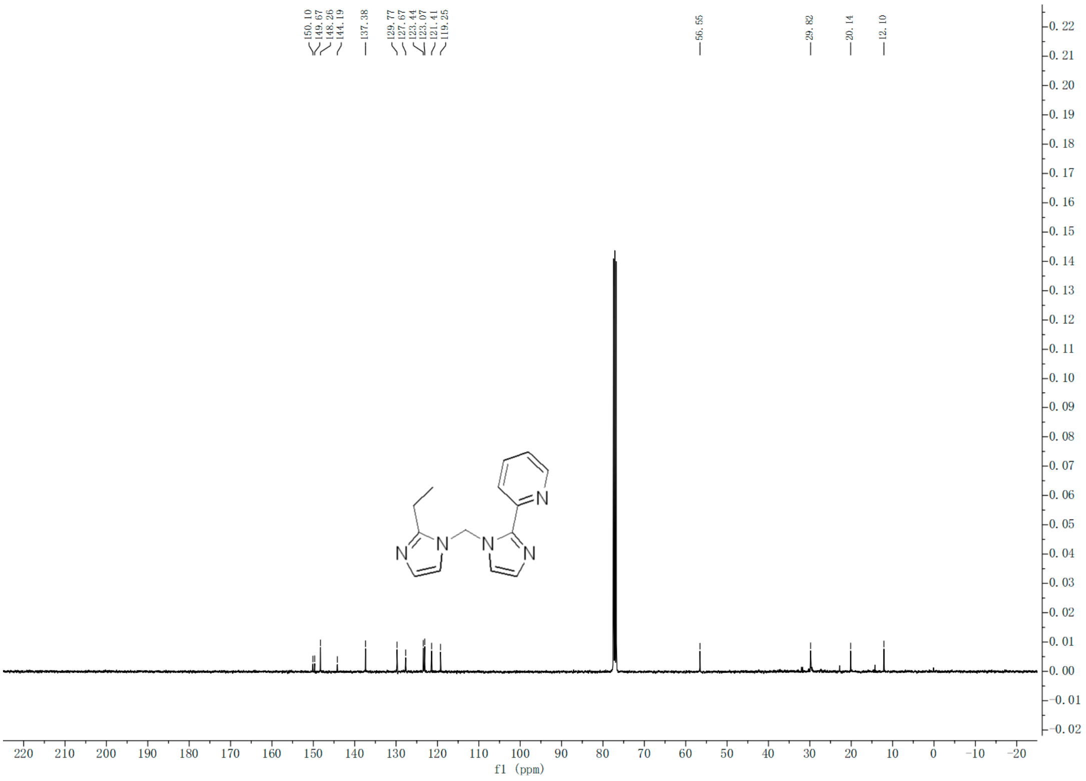

^{1}H NMR of **A15**

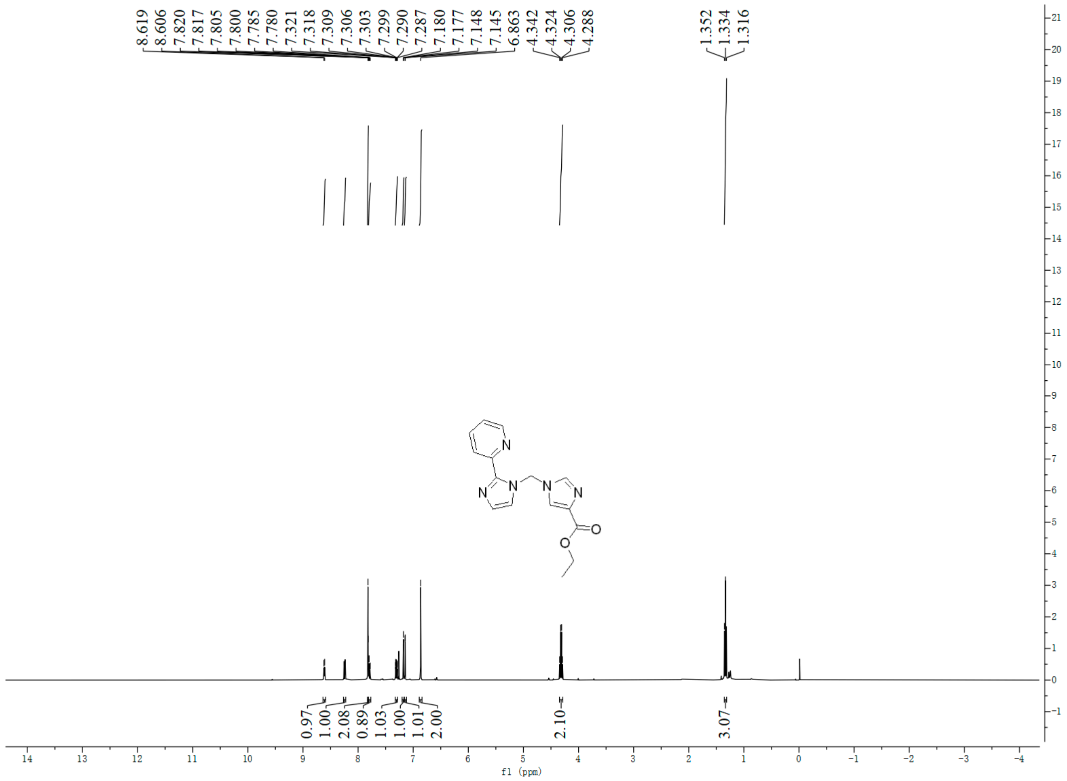

^{13}C NMR of **A15**

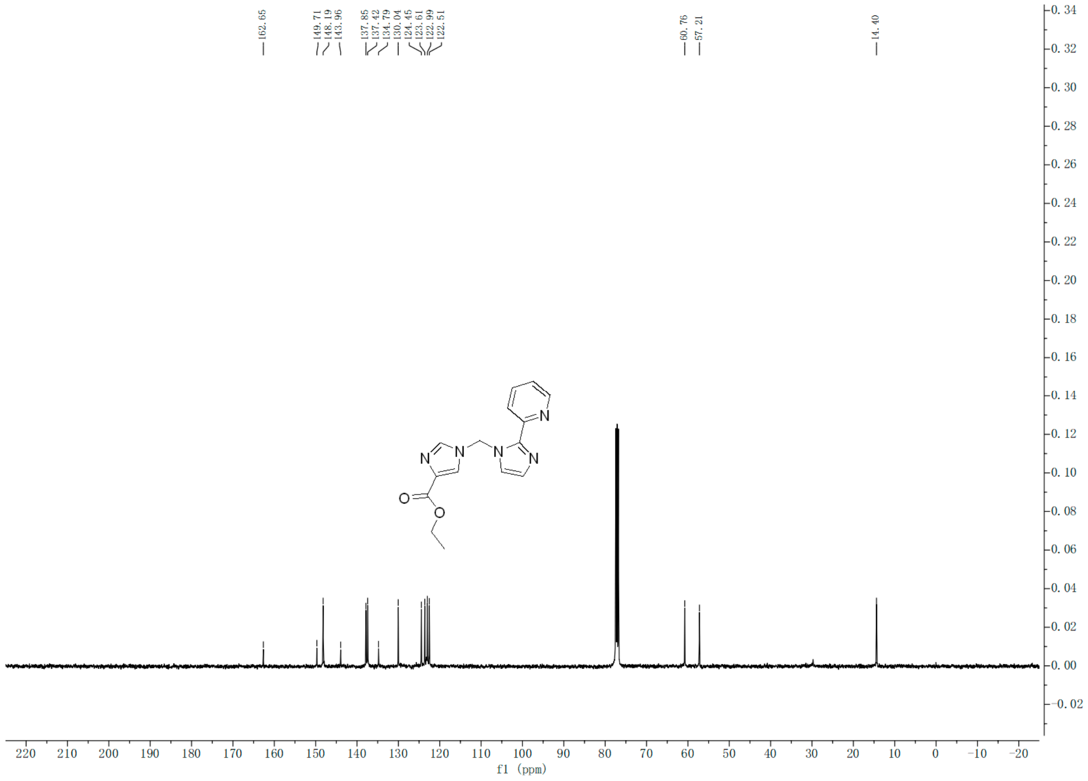

^{1}H NMR of **A16**

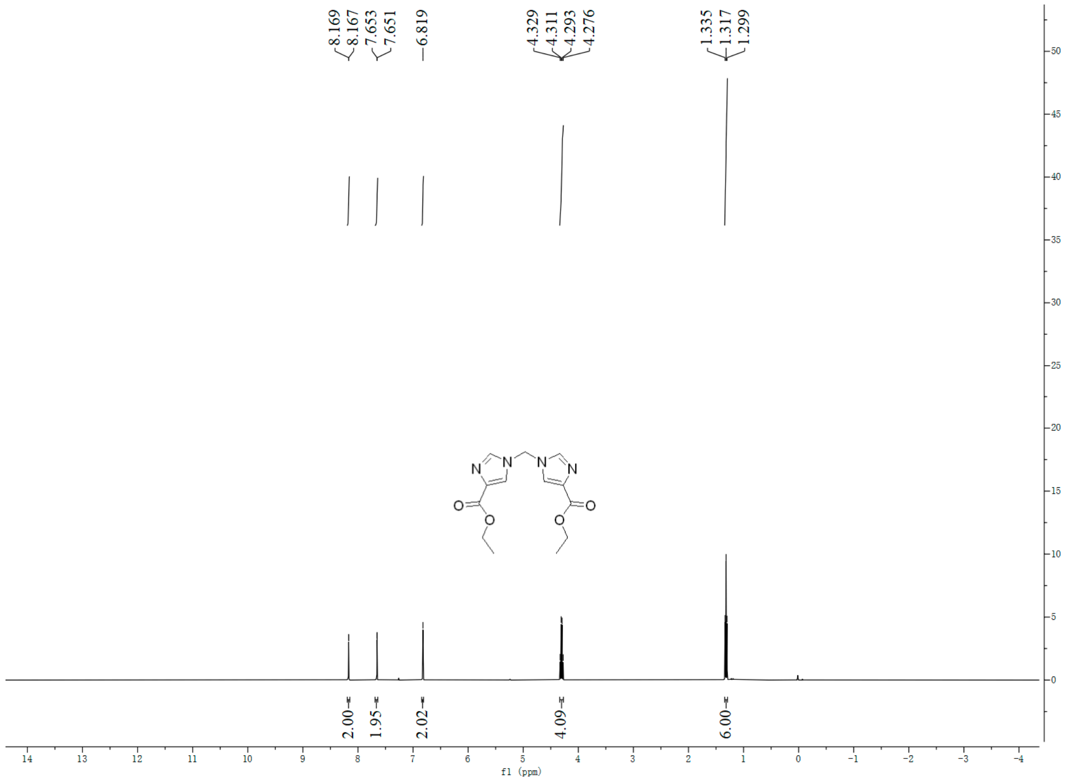

^{13}C NMR of **A16**

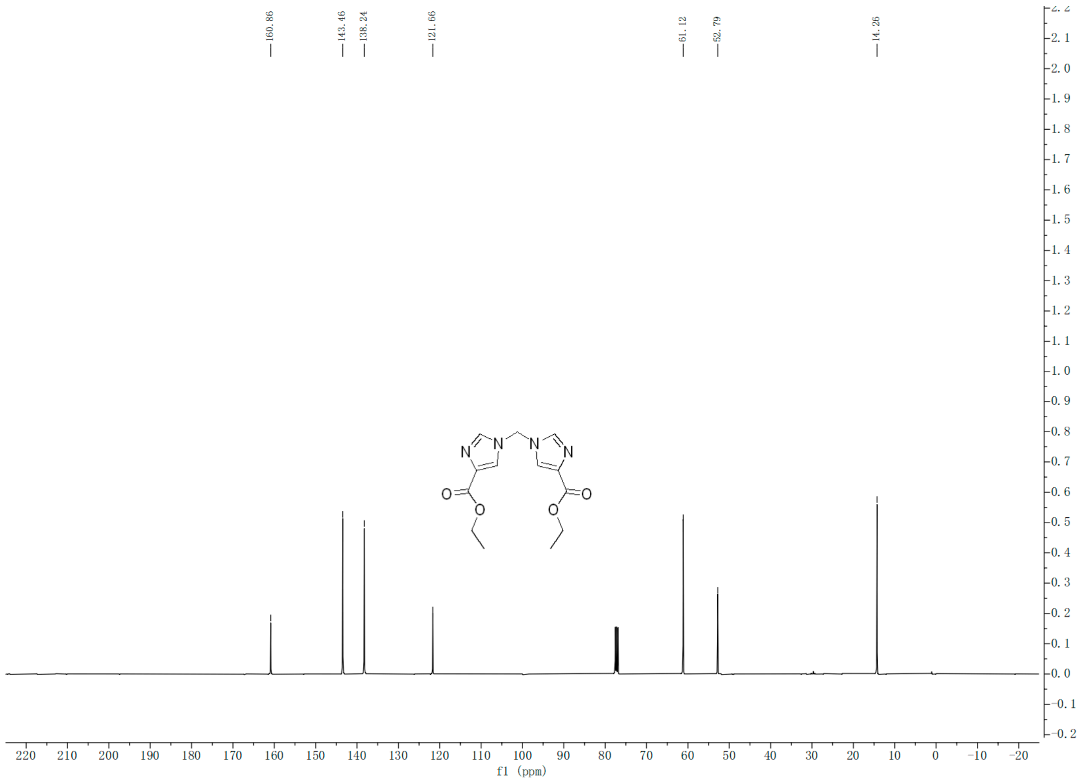

^{1}H NMR of **A17**

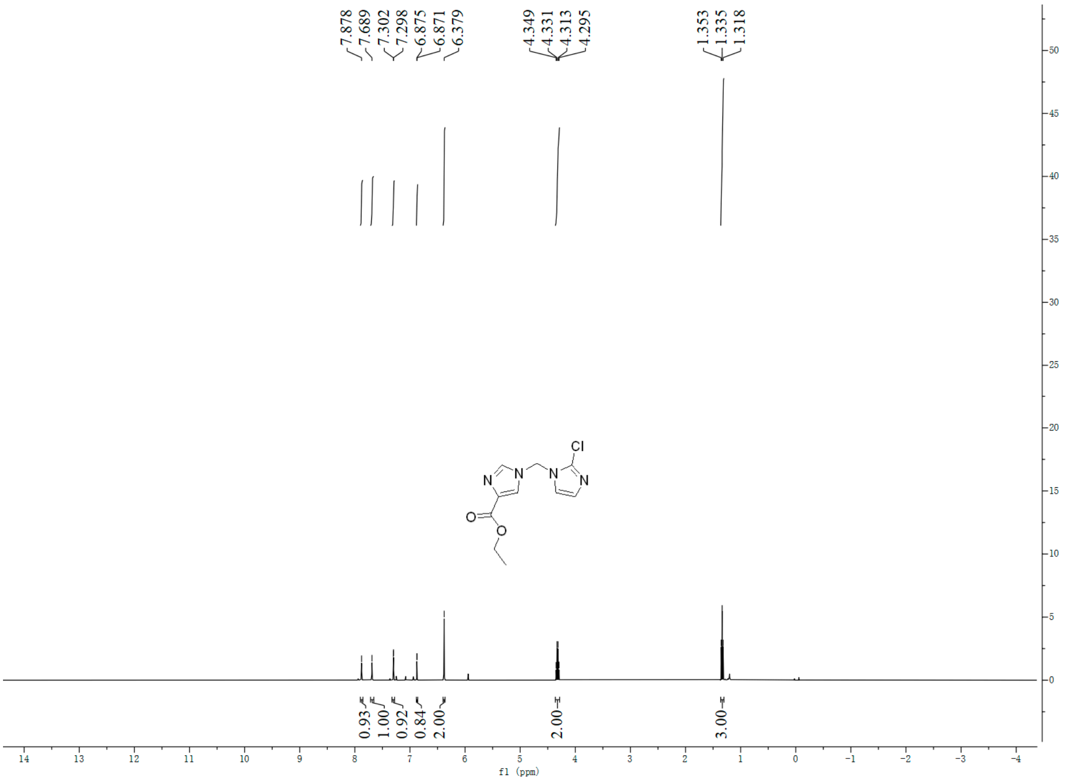

^{13}C NMR of **A17**

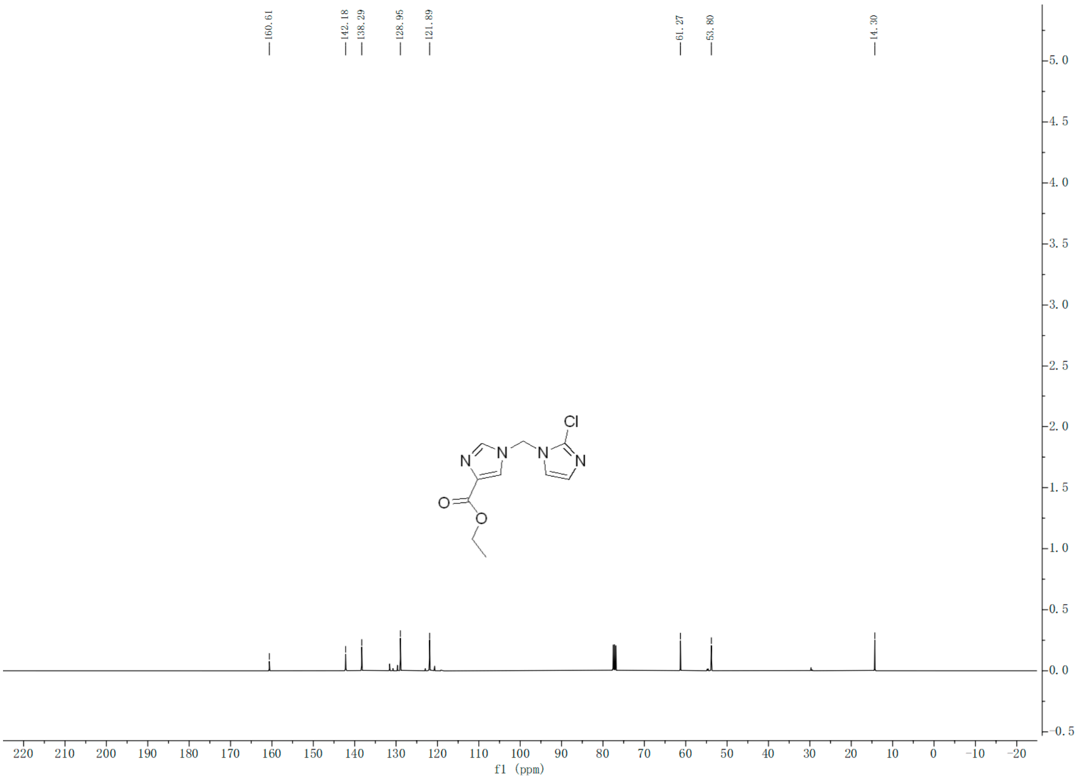

^{1}H NMR of **B1**

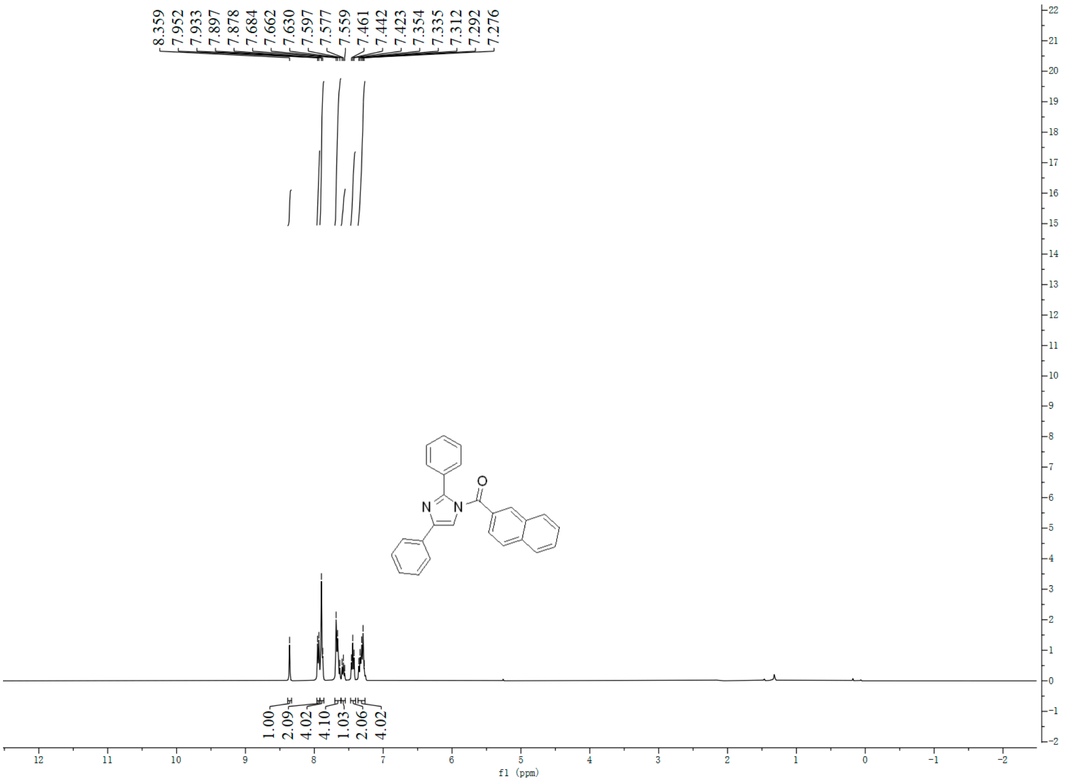

^{13}C NMR of **B1**

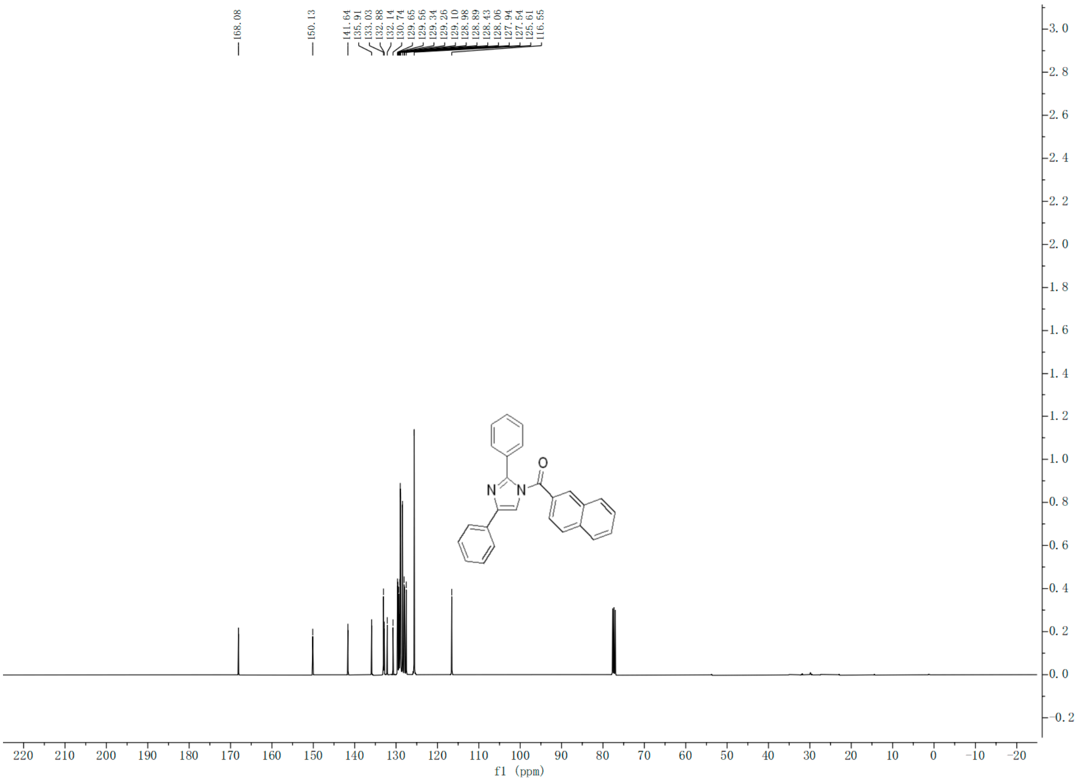

^{1}H NMR of **B2**

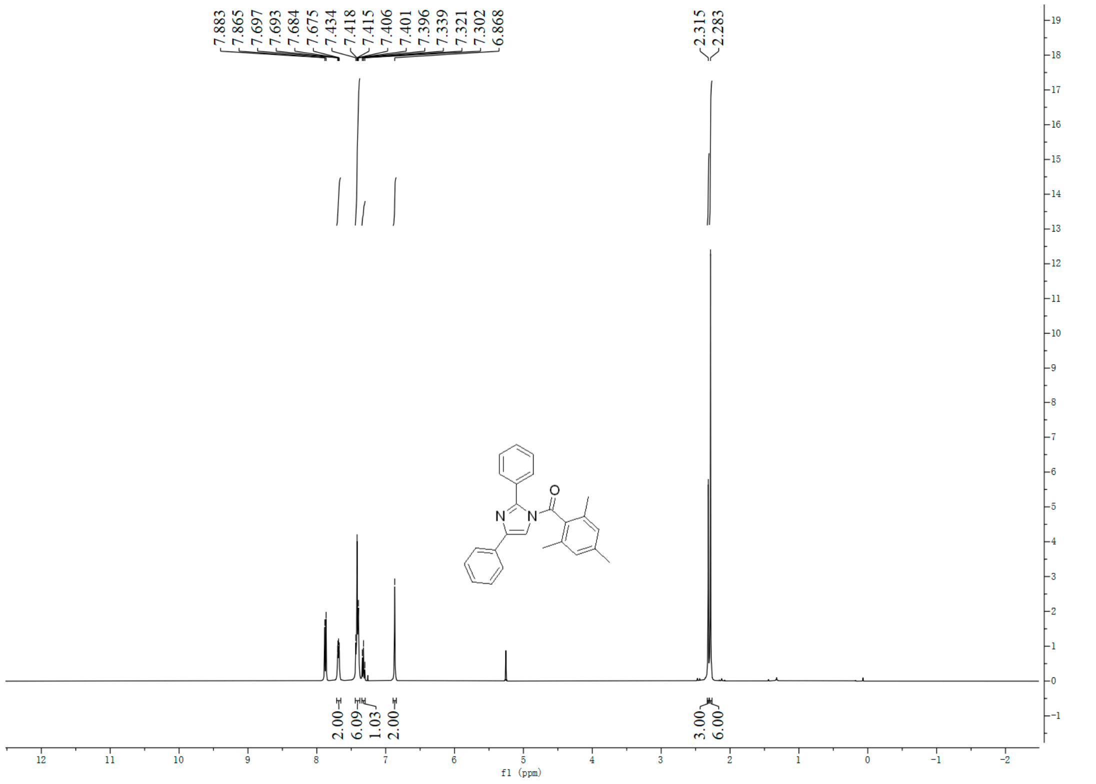

^{13}C NMR of **B2**

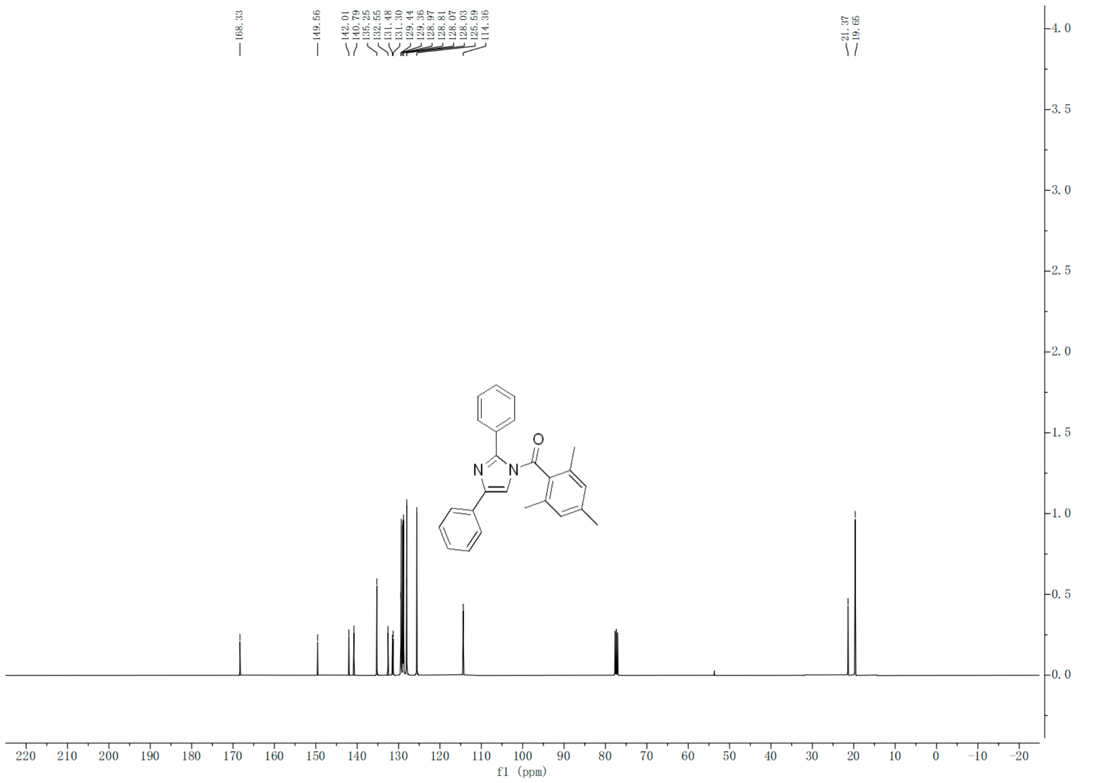

^{1}H NMR of **B3**

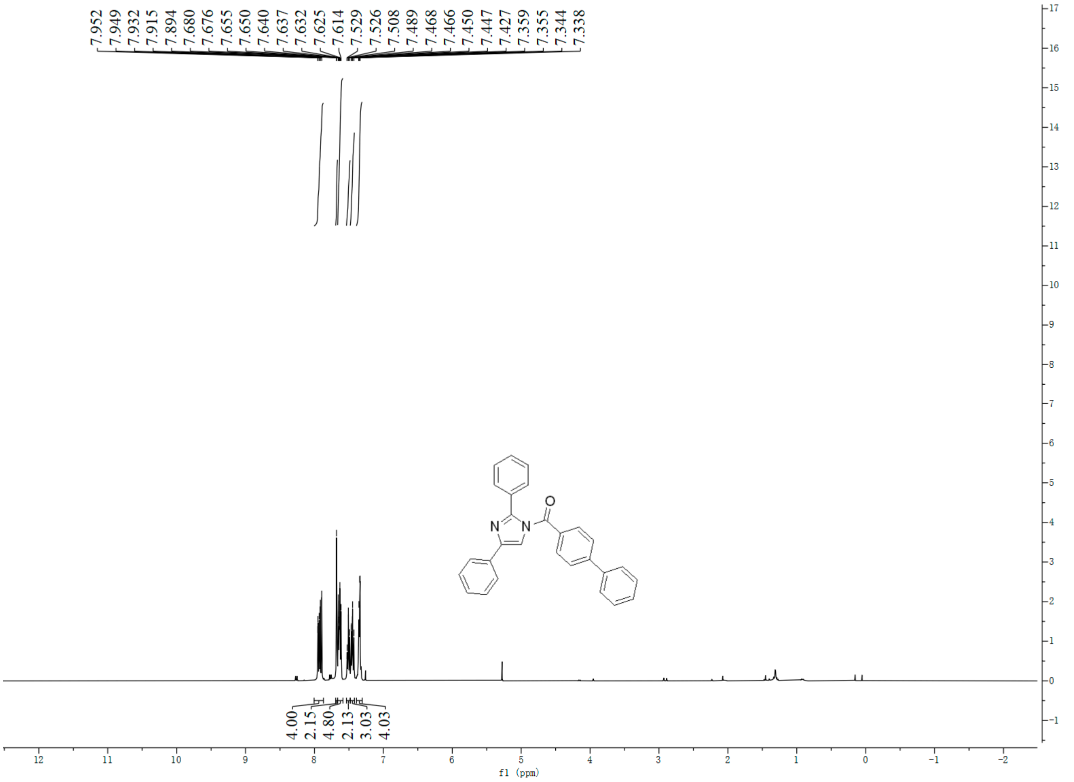

^{13}C NMR of **B3**

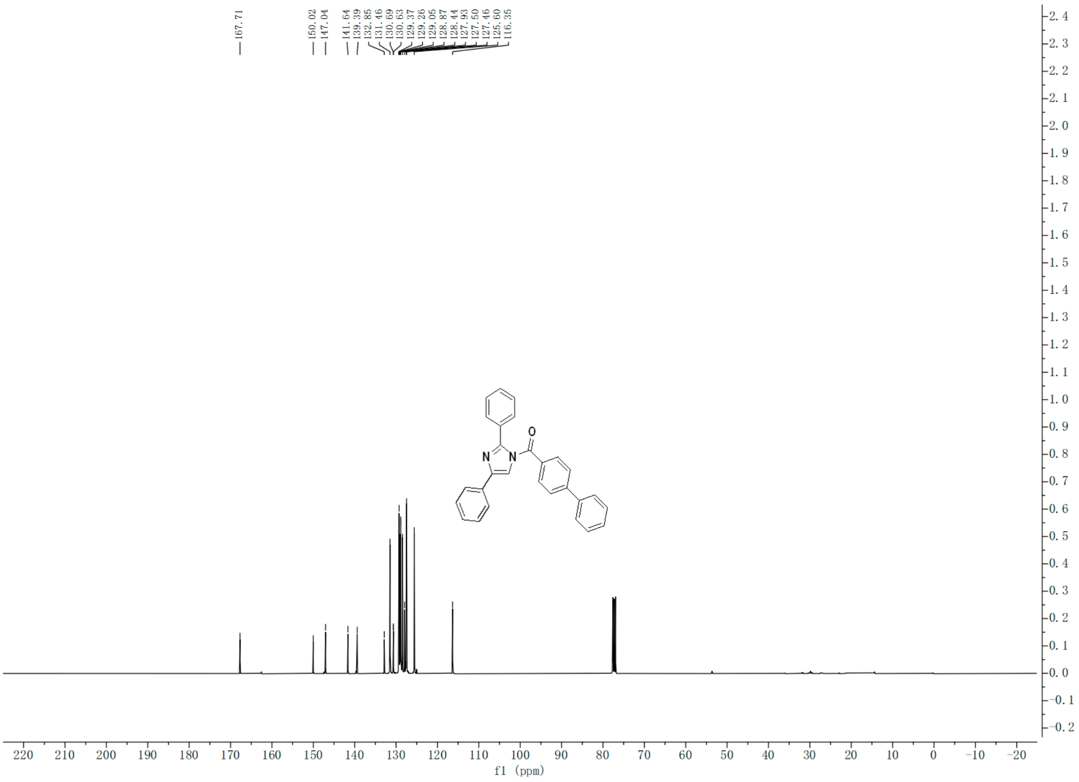

^{1}H NMR of **B4**

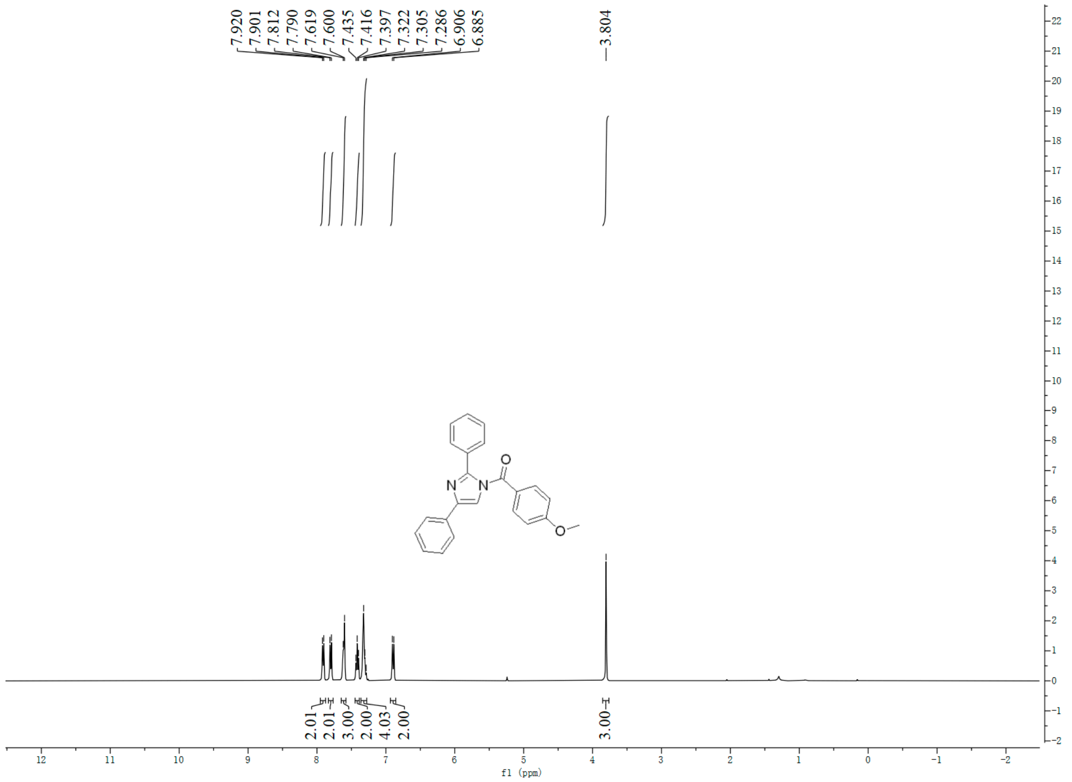

^{13}C NMR of **B4**

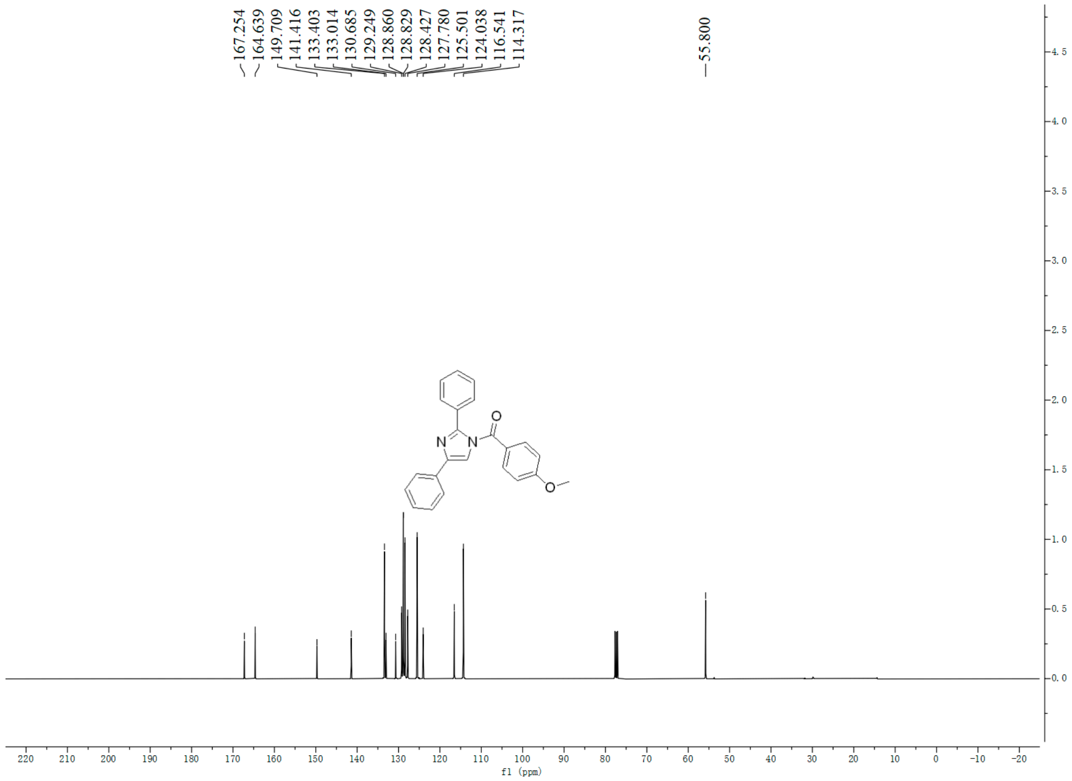

^{1}H NMR of **B5**

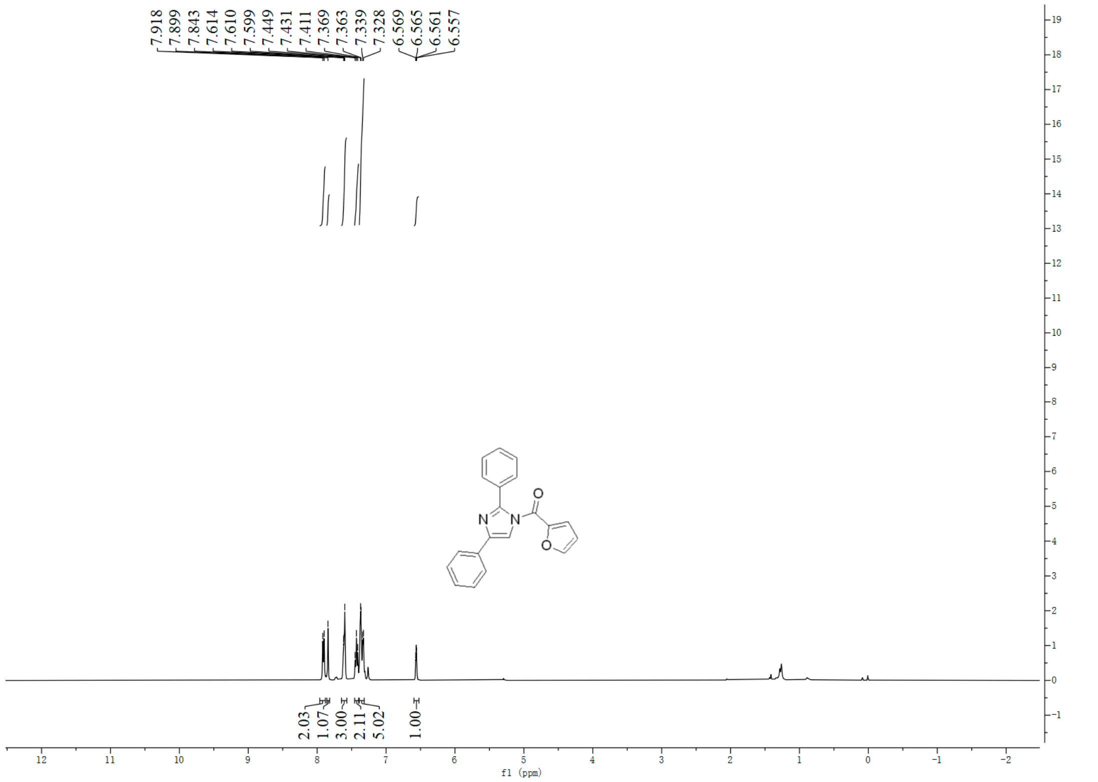

^{13}C NMR of **B5**

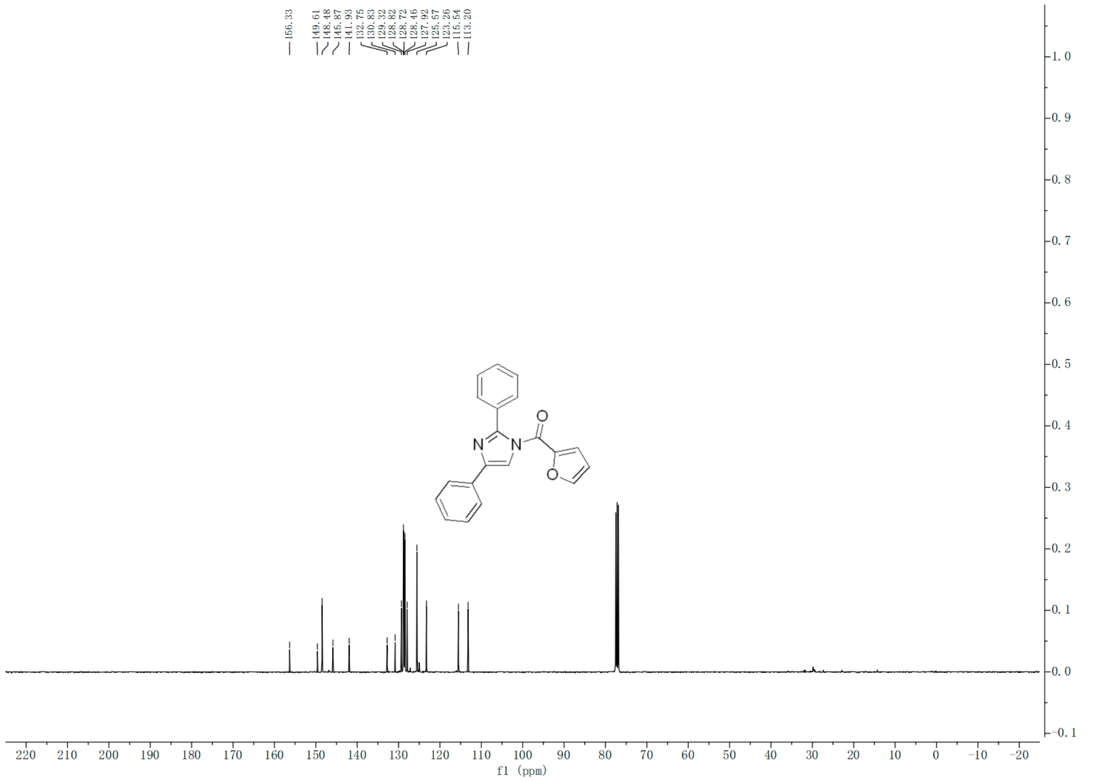

^{1}H NMR of **B6**

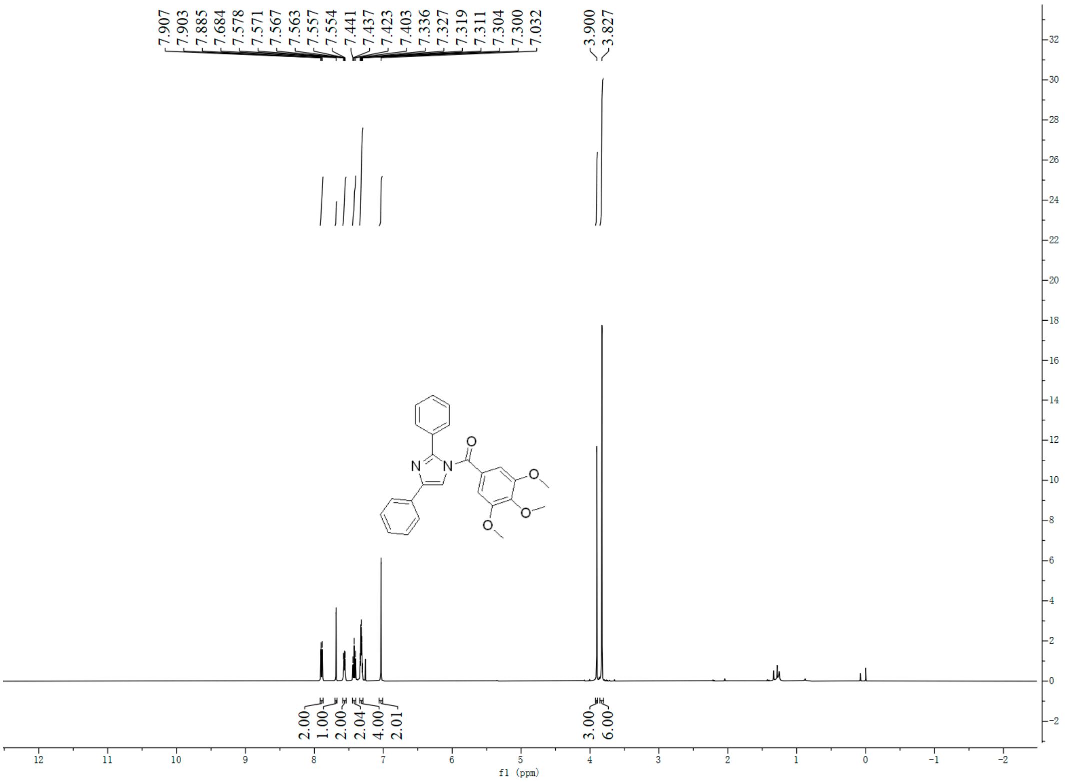

^{13}C NMR of **B6**

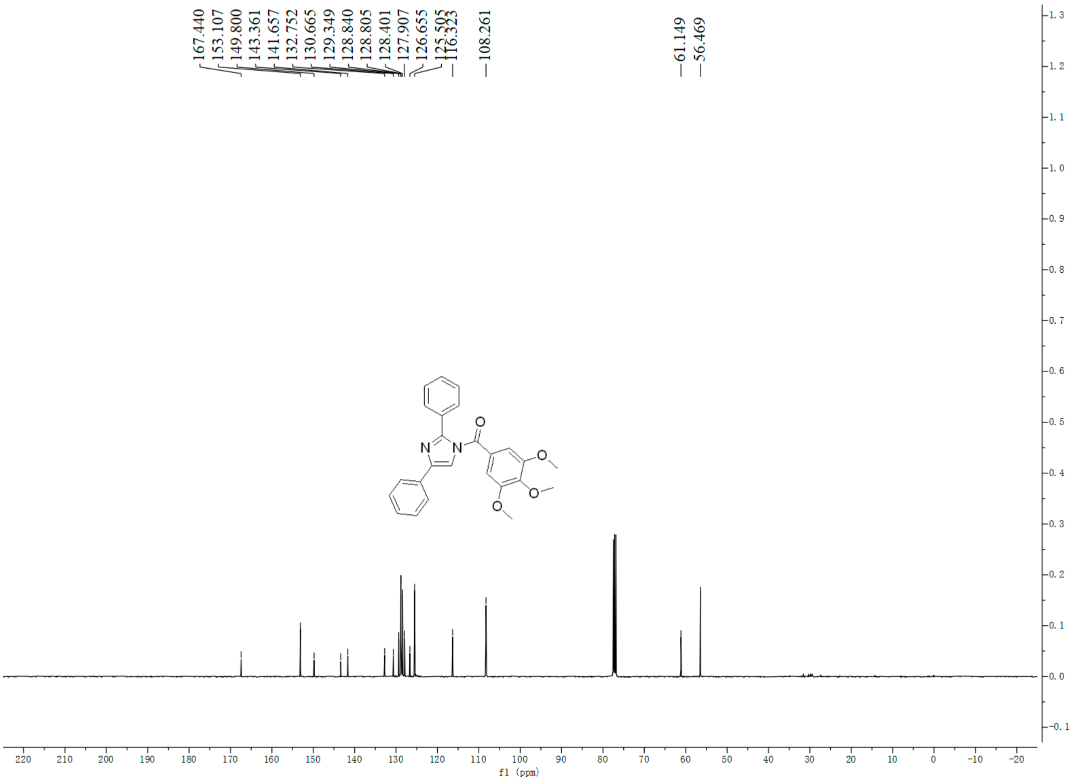

^{1}H NMR of **B7**

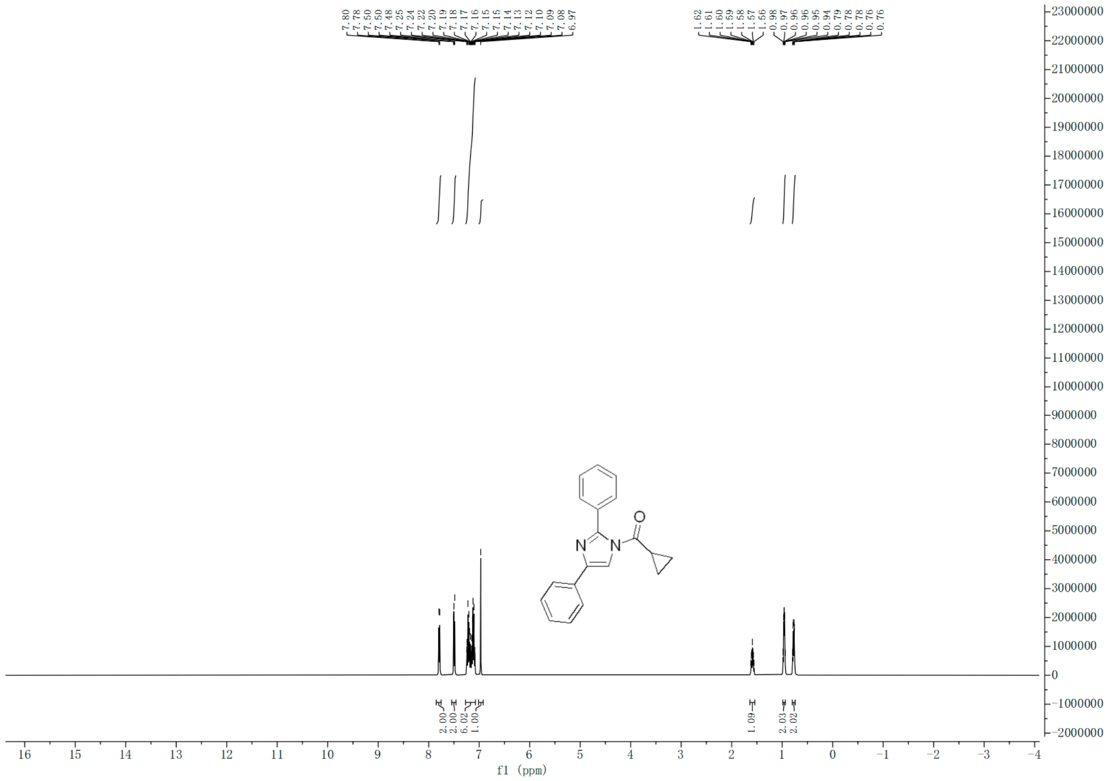

^{13}C NMR of **B7**

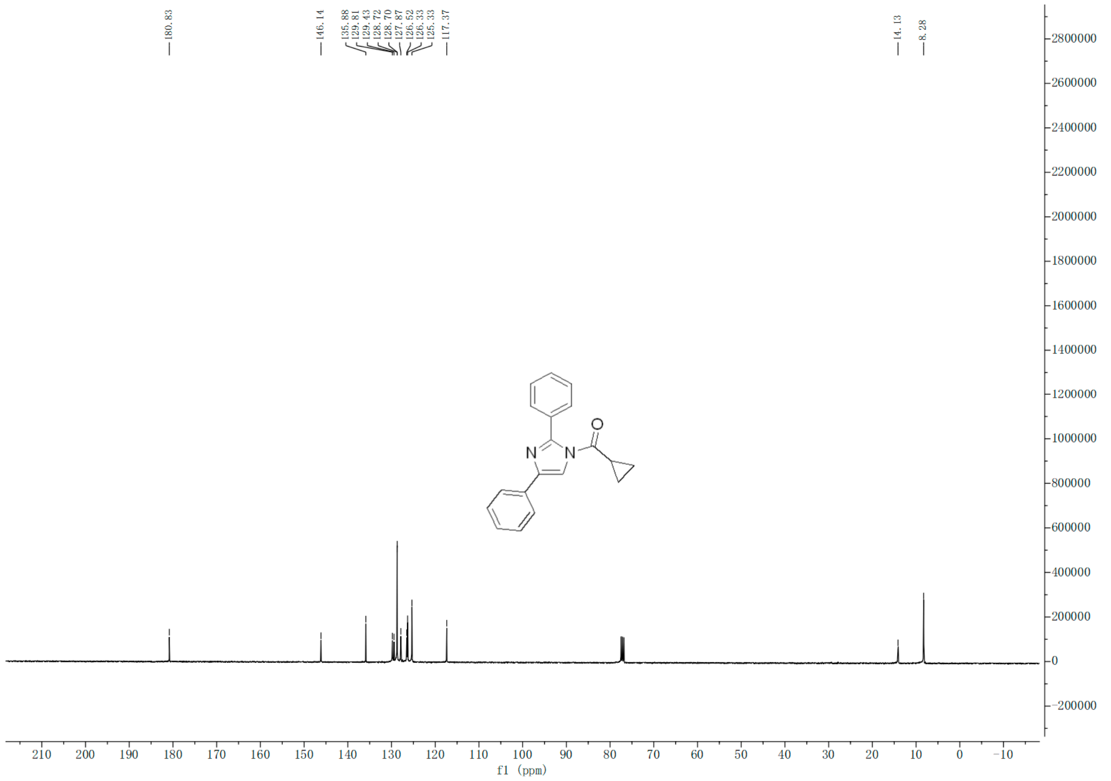

^{1}H NMR of **B8**

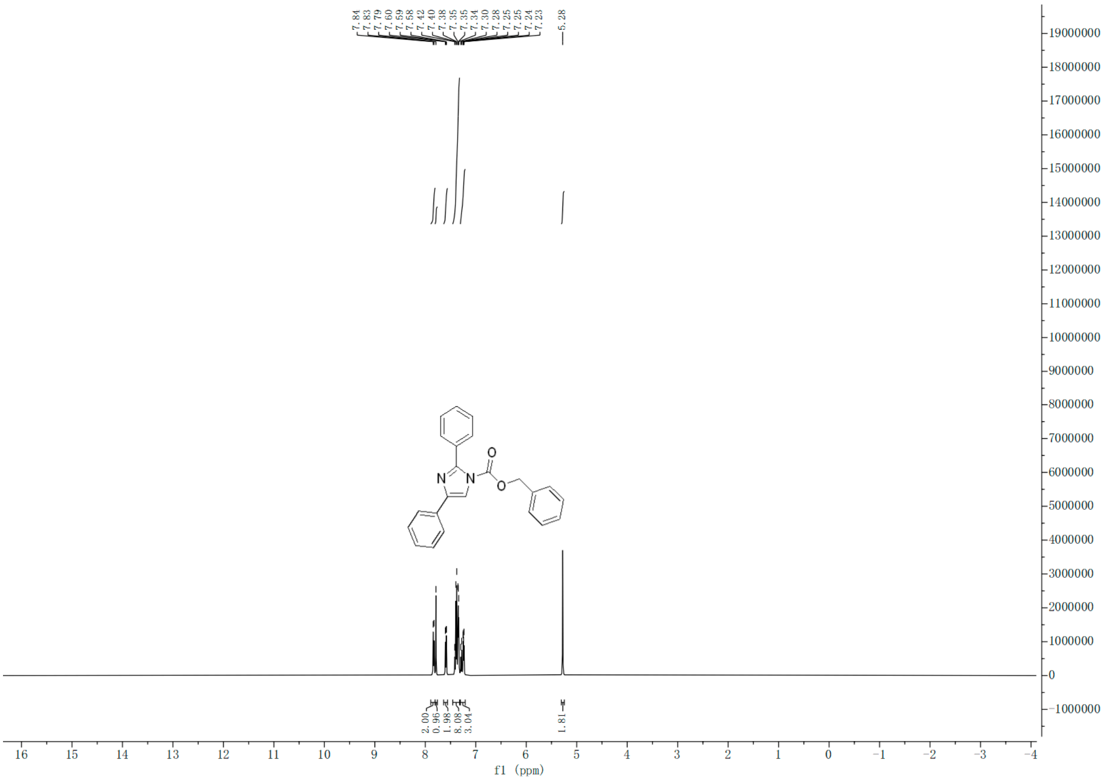

^{13}C NMR of **B8**

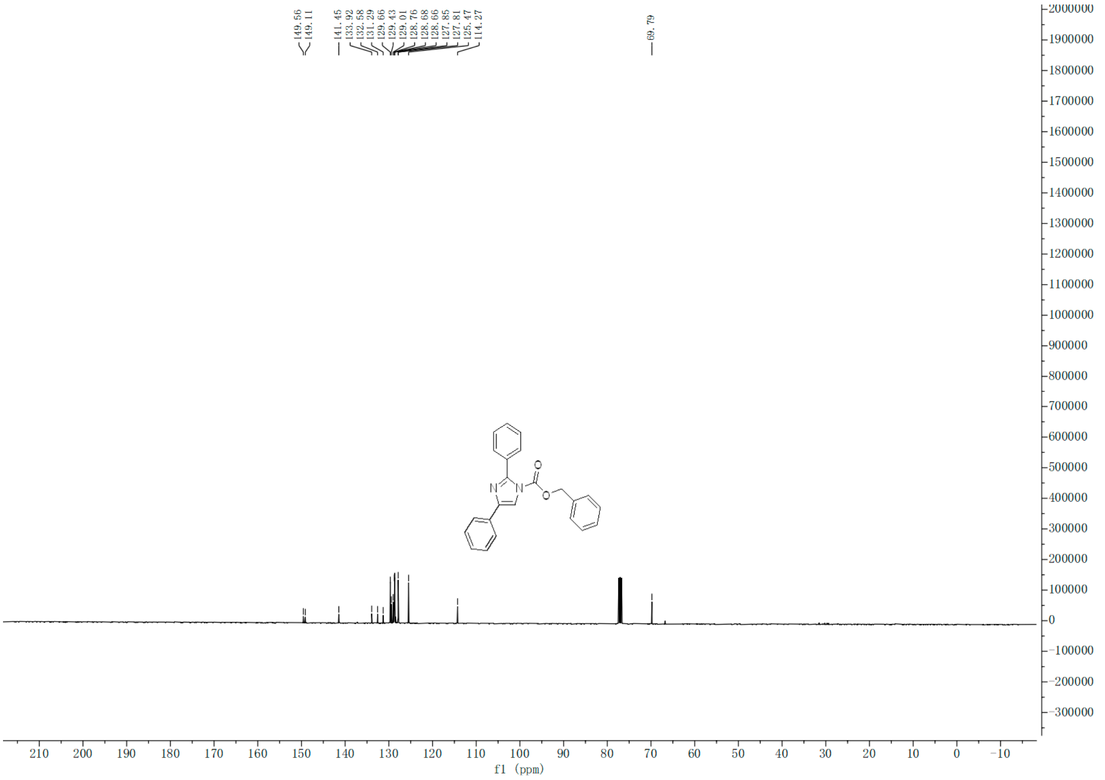

Supplement: Supplementary file 1 [file molecules-29-02301-s001.zip › molecules-2986837-supplementary.pdf]
